# The Vascular Flora of Sirente-Velino Regional Park (Abruzzo, Central Italy)

**DOI:** 10.3390/biology15131093

**Published:** 2026-07-07

**Authors:** Fabio Conti, Igino Chiuchiarelli, Marinella Miglio, Bruno Petriccione, Bruno Santucci, Fabrizio Bartolucci

**Affiliations:** 1 Scuola di Bioscienze e Medicina Veterinaria, Università di Camerino-Centro Ricerche Floristiche dell’Appennino, Parco Nazionale del Gran Sasso e Monti della Laga, San Colombo, 67021 Barisciano, Italy; fabio.conti@unicam.it; 2 Ente Parco Naturale Regionale Sirente Velino, Viale XXIV Maggio snc, 67048 Rocca di Mezzo, Italy; direzione@sirentevelino.it; 3 Via dei Merletti 8, 67100 L’Aquila, Italy; marinella.miglio3@alice.it; 4 Carabinieri, Forest and Environmental Protection Department, 00187 Roma, Italy; b.petriccione@gmail.com; 5 Via Fonte di Là 2, 67062 Magliano de’ Marsi, Italy; bruno1.san@alice.it; 6 Dipartimento di Biologia Ambientale, Sapienza Università di Roma, Piazzale Aldo 4 Moro 5, 00185 Roma, Italy

**Keywords:** Abruzzo, Apennine, biodiversity, conservation, endemics, taxonomy, vascular flora

## Abstract

We present the first vascular flora of Sirente-Velino Regional Park, a protected area located in central Apennine (Abruzzo, Central Italy), covering 550 km^2^. The floristic inventory was compiled based on extensive field surveys conducted from 1990 to 2025. Thanks to specific funding secured for the final three years (2023–2025), field trips became systematic, aimed at covering the entire territory of the park, including its diverse habitats across different seasons. Furthermore, an analysis of the relevant literature was performed, alongside a comprehensive review of herbarium specimens housed at APP, FI, NAP, and RO. The park’s flora comprises 2066 taxa (1370 species and 696 subspecies, including 11 hybrids), belonging to 117 families and 671 genera, with 122 classified as alien. Notably, 169 taxa are endemic to Italy. Our study reports 234 taxa for the first time within the park, while 52 remained unconfirmed in recent times and 22 are of doubtful occurrence. Five taxa are newly recorded for the Abruzzo region: *Alchemilla alpinula*, *A. obtusa*, *A. vulgaris*, *Hyacinthoides non-scripta*, and *Salsola tragus*. Additionally, the revision of APP herbarium specimens led to the exclusion of *Euphorbia tommasiniana* from the regional flora, reassigning the material to the alien *E. saratoi*. The species richness of the native flora exceeds expectations by 50.8%, a finding likely driven by the area’s significant geographical, geological, and microclimatic diversity, which results in high environmental heterogeneity. Conversely, alien taxa are 5.6% lower than expected, probably due to the high ecological integrity of the park, a predominantly mountainous territory with low population density and minimal anthropogenic disturbance. Representing 20.6% of the entire Italian flora, the Sirente-Velino Regional Park stands out as one of Europe’s protected areas with the greatest diversity of vascular plants.

## Simple Summary

This study aimed to create the first comprehensive, updated inventory of vascular plants of Sirente-Velino Regional Park (Abruzzo, Central Italy) to understand its diversity, unique species, and conservation threats. By combining historical records with extensive field surveys from 1990 to 2025, we identified 2066 species and subspecies (including 11 hybrids), which represents over 20% of all Italian plant taxa. This remarkable richness makes the park one of Europe’s most diverse protected areas. Ultimately, this research provides park authorities with a vital scientific baseline. It serves society by giving decision-makers the exact tools needed to prioritise conservation efforts, safeguard fragile ecosystems, and protect natural heritage against ongoing environmental changes.

## 1. Introduction

Comprehensive knowledge of the flora within a given geographical area (i.e., floristic inventory or checklist) provides a foundational source of information for biogeographical, ecological, and evolutionary studies and is paramount to combating biodiversity loss [1,2]. In the current era of rapid global environmental change, local floristic studies represent crucial baselines for monitoring ecological transformations and prioritising conservation efforts. Rather than being simple localised catalogues, fine-scale inventories serve as the essential building blocks for broader macroecological models and global biodiversity assessments. They enable the identification of specialised micro-habitats, localised endemics, and peripheral populations that large-scale global grids frequently overlook. Consequently, downscaling our understanding of plant diversity to the local level is imperative, as it provides the empirical resolution needed to safeguard global genetic lineages and preserve evolutionary potential under shifting climatic conditions. Specifically, floristic inventories serve as indispensable tools for effective ecosystem management and strategic conservation planning. When coupled with concrete in situ and ex situ actions, these inventories become crucial for safeguarding the most vulnerable plant species and habitats [3,4,5]. To ensure the effectiveness of protection measures, knowledge of flora (e.g., species distribution) must be constantly updated through periodic monitoring, with particular reference to the most endangered species and plant communities [6,7,8]. Furthermore, floristic inventories are essential tools for monitoring alien flora in urbanised (e.g., [9,10,11,12]) and in natural areas. They enable the early interception of potentially invasive species while they are still confined to urban zones, thereby facilitating eradication with relatively low cost and effort [13,14]. Within this framework, evaluating alien plant dynamics requires a multi-scale approach that goes beyond classical national boundaries. Biological invasions are increasingly addressed at finer spatial resolutions by distinguishing “regional aliens” (taxa native to a country but introduced by human activity into an administrative region outside of their natural range) and “local aliens” (taxa native to a region but alien to the specific study area or protected territory). Tracking these internal range shifts is crucial for modern conservation, as local and regional introductions often represent insidious, overlooked drivers of ecological homogenisation and native community displacement [15].

The mountainous landscapes of Abruzzo are characterised by a remarkable botanical heritage. According to the most recent updates, the vascular flora of the region comprises 3634 native and alien taxa [16,17], ranking Abruzzo, considering only native species, third in Italy for floristic richness after Liguria and Friuli Venezia Giulia [18]. This diversity is primarily concentrated within the region’s major mountain ranges. For instance, the Gran Sasso and Monti della Laga National Park hosts 2678 taxa (species and subspecies) [19,20,21,22], followed by the Maiella National Park with 2334 [23,24,25], and the Abruzzo, Lazio, and Molise National Park with 2116 taxa [21,22,26,27,28].

Within this context, the Sirente-Velino Regional Park (hereafter PRSV) has consistently documented a high degree of biological diversity. Botanical investigations in the area date back to the pre-Linnaean era, when Luigi Anguillara visited Abruzzo in 1545 and reported the first two species for the park area (*Conium maculatum* L. and *Daphne sericea* Vahl). However, the first significant floristic contribution was provided by the Venetian naturalist Gian Battista Brocchi [29,30,31], who documented approximately one hundred plants from Mt. Velino and Gran Sasso. Other noteworthy historical milestones include the studies of Michele Tenore [32,33] on Mt. Velino and Enrico Groves [34] on Mt. Sirente, followed in more recent times by the fundamental contributions of Giuliano Montelucci, who recorded 599 taxa for Mt. Velino [35]. The most comprehensive past reports for the PRSV are credited to Lucchese and Lattanzi [36], who reported 576 taxa for Mt. Velino (including 512 new records compared to previous studies), bringing the total known flora of the massif to 1028 taxa. Furthermore, Bruno Petriccione reported 639 taxa (species or subspecies) for the Velino massif; however, this inventory also included the Duchessa Mountains, which are located in the Lazio region and outside the Sirente-Velino Regional Park [37]. Finally, Paolo Guarrera and Fernando Tammaro studied the flora of Mt. Sirente and its neighbouring areas, documenting 1292 taxa [38].

Despite this long history of exploration, certain areas of the Park remain insufficiently investigated; these include the tectonic-karstic plateaus—such as Altopiano delle Rocche, Piani di Pezza, Campo di Rovere, and Piana di Ovindoli—as well as the Aterno Valley, Monti della Magnola, and various sectors of Mt. Velino. While the most recent data estimate the PRSV flora at 1650 taxa [39], a formal, comprehensive floristic list has never been published; instead, the recent literature has focused primarily on single species of phytogeographical or conservation interest [22,27].

Accordingly, the main objective of this study is to provide an updated and comprehensive analysis of the vascular flora of the PRSV. Specifically, we address the following four key questions: (1) What is the total floristic diversity of the park in relation to its territory? (2) Which endemic and phytogeographically significant species are present in the area? (3) What is the current composition and status of the alien flora? and (4) What are the main conservation priorities and threats facing the park’s flora? These questions were addressed by synthesising data from the published literature, conducting extensive field investigations, and reviewing historical herbarium specimens.

## 2. Materials and Methods

### 2.1. Study Area

#### 2.1.1. Geography and Geomorphology

**Figure 1:**
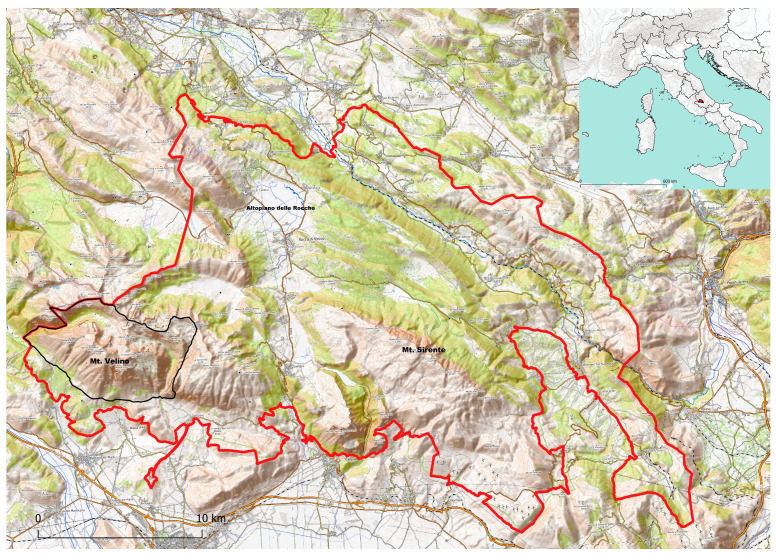
Map of Sirente-Velino Regional Park (red borders) and Monte Velino Nature Reserve (black borders); map of Italy showing the location of the Park.

**Figure 2:**
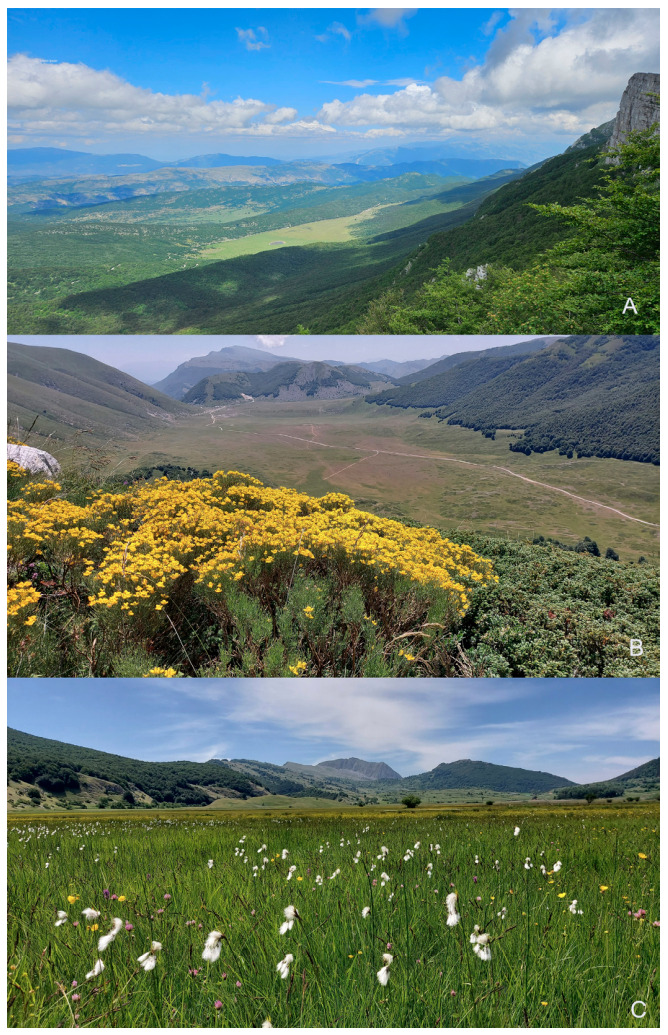
Karst plateau habitats of exceptional natural value. (**A**) Grasslands of Sirente (Prati del Sirente); (**B**) grasslands of Pezza (Piani di Pezza), with the rare *Genista radiata* in the foreground, and associated shrublands; (**C**) wetlands of Ovindoli (Piana di Ovindoli), with *Eriophorum latifolium* (Photos by F. Bartolucci).

**Figure 3:**
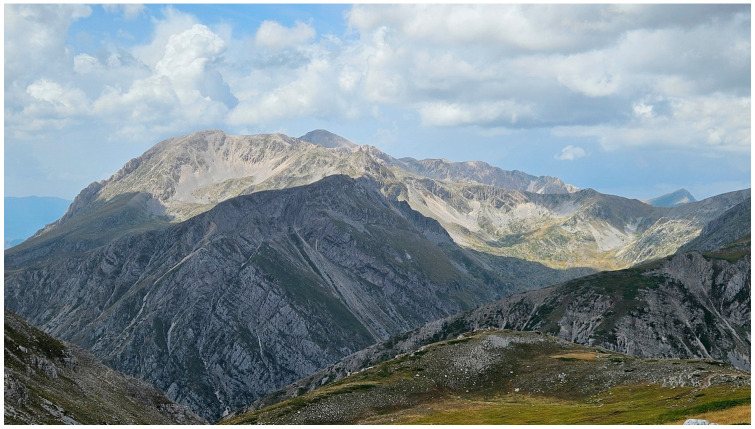
Velino Massif (photo by F. Bartolucci).

The Sirente-Velino Regional Nature Park, established by the Abruzzo Region with Regional Law No. 54 of 13 July 1989, covers an area of approximately 55,000 hectares and is one of the most significant natural areas in the Central Apennines (Figure 1). Two years earlier, the Monte Velino Nature Reserve (Ministerial Decree no. 427 of 21 July 1987) had already been established, covering an area of approximately 4000 hectares, entirely within the park. The park’s territory included three areas of the Natura 2000 network: the Special Areas of Conservation IT7110206 “Monte Sirente and Monte Velino” and IT7110075 “Serra and Gole di Celano—Val d’Arano”, and the Special Protection Area IT7110130 “Sirente-Velino”. Geographically, the park is bordered to the northeast by the Aterno river valley, to the west by the Campo Felice plain and the Duchessa mountains, and to the south by the Fucino plain. The territory is home to a remarkable variety of geomorphological and environmental features, such as valuable tectonic-karst plateaus (Campo Felice, Prati del Sirente, Altopiano delle Rocche, Piani di Pezza, Campo di Rovere, Piana di Ovindoli, Figure 2), important rock habitats (e.g., Gole di Celano, Vallone di Teve) and high mountain ranges exceeding 2000 m above sea level (Monte Velino 2486 m, Monte Sirente 2348 m, Monti della Magnola 2220 m, Figure 3), in contrast to landscapes with a gentler morphology such as the Aterno Valley. We included, in the study area, the Gole di San Venanzio Nature Reserve, adjacent to the PRSV territory, and a continuation of the Aterno Valley, where the lowest altitude (300 m above sea level) of the investigated area is recorded.

#### 2.1.2. Geology

Geologically, the Sirente-Velino paleogeographic-structural unit belongs to the Lazio-Abruzzo carbonate platform, characterised by limestone–dolomite and limestone sequences [40]. The structure appears as a NW–SE monoclinal dip towards the southwest, overridden to the northeast on Miocene–Pliocene flysch deposits. During the Pliocene–Pleistocene, the general uplift of the Apennine chain led to the establishment of continental lithofacies, with lacustrine, alluvial, and red earth sequences derived from carbonate dissolution. The mountain range has a composite structural configuration, with monoclinal units disarticulated by Mesozoic tectonics and overlapped towards the Adriatic by Alpine orogenesis. The predominant lithologies are Cretaceous limestones of the internal platform with plates of detrital-organogenic limestones more frequent in the eastern margins, with the presence of turbiditic sequences based on sandstone–clay alternations in the slopes. The southwestern margin is structured by the overlap of carbonate lithofacies on sandstone–pelitic successions related to the Gran Sasso succession [41].

#### 2.1.3. Climate

The territory of the Sirente-Velino Regional Park has two very different climates. The southern part of the Sirente-Velino massif and the Fucino Plain is characterised by low average annual rainfall (700 to 1000 mm) and a rather pronounced summer drought period. The highest rainfall is recorded in the winter months (winter solstice rainfall pattern) and the minimum daily temperature remains above zero continuously for three to four months of the year (from June to September): the climate is therefore arid Mediterranean, with a tendency towards a certain continentality (low rainfall and high annual temperature ranges). The northern part, i.e., the Altopiano delle Rocche plateau and the northern slope of Mount Sirente, enjoys much higher rainfall (from 1200 to 1500 mm) and no periods of drought, even in the height of summer. The better seasonal distribution of rainfall (also high in spring and autumn) makes the environment much more favourable for plants, and the period of the year without frost is longer (four to five months). The climate in the latter case is closer to a “Mediterranean-mountain” climate, characterised by equinoctial rainfall and a tendency towards a certain oceanicity, widespread across almost all the massifs of the Central Apennines.

#### 2.1.4. Vegetation

The vegetation of PRSV can be divided into five altitude zones, corresponding to fundamental climatic differences.

The Mediterranean zone of evergreen forest, characterised by higher temperatures and lower rainfall, is represented only by a few remnants of rocky *Quercus ilex* L. forest, always found on the steepest and rockiest southern slopes.

The Central European belt of deciduous oak forests generally extends from approximately 500 to 1000 m, reaching up to 1500 m in the southern part of the Mt. Velino due to very particular climatic and geomorphological characteristics. Thermophilic oak forests with *Quercus pubescens* Willd. subsp. *pubescens* are the most representative formation; only locally, on poorly permeable soils, are there mesophilic oak forests dominated by *Quercus cerris* L. The multi-species tree layer is dominated by *Quercus pubescens* subsp. *pubescens*, *Fraxinus ornus* L. subsp. *ornus,* and *Ostrya carpinifolia* Scop. Along the course of the Aterno River and the few other watercourses in the park, there are hygrophilous thickets with a shrubby or arboreal structure, differentiated according to altitude with *Salix alba* L., *S. purpurea* L. subsp. *purpurea* and *S. eleagnos* Scop., sometimes accompanied by *S. apennina* A.K.Skvortsov and *Populus nigra* L.

Starting at an altitude of 1000–1200 m, the vegetation is clearly characterised by the deciduous beech forest of the sub-Atlantic belt. The dominant, if not exclusive, forest community is represented by *Fagus sylvatica* L., which is almost always found in pure stands. The only other forest associations in this belt consist of patches of evergreen coniferous forest, which are undoubtedly man-made (and therefore foreign to the natural landscape).

In the southern part of the Mt. Velino and Mt. Sirente, beech forests have mostly been replaced by shrublands dominated by *Arctostaphylos uva-ursi* (L.) Spreng. and *Juniperus communis* L. var. *saxatilis* Pall. [42] due to mesoclimatic peculiarities.

A special case is represented by the large plateaus located between 1000 and 1600 m above sea level, mostly tectonic depressions with karst formations and sinkholes, widely distributed in the central part of the park, characterised for the most part by vast semi-natural meso-hygrophilous grasslands of great ecological value [43,44], habitats often with exclusive, protected, and threatened plant species as *Allium angulosum* L., *Juncus atratus* Krock., *Klasea lycopifolia* (Vill.) Á.Löve & D.Löve, *Lathyrus pannonicus* subsp. *asphodeloides* (Gouan) Bässler, *Serratula tinctoria* L., *Tulipa pumila* Moench, and *Sesleria uliginosa* Opiz.

Above 2000 m, the herbaceous communities of the high peaks give the summit area (Mediterranean high-mountain) an “alpine” feel. However, between the beech forest belt and the high-altitude belt, there are often dense prostrate shrublands of dwarf juniper (*Juniperus communis* var. *saxatilis*). The vegetation of the belt above the tree line [45], characterised by natural herbaceous communities, is basically represented by a particular type of open high-altitude xerophytic grassland (*Sesleria* grassland), dominated by clumps of grasses (*Sesleria juncifolia* Suffren) and sedges (*Carex kitaibeliana* Degen ex Bech.).

The high altitude (alpine) belt, found only on Mt. Velino between 2300 and 2500 m, is home to patchy vegetation consisting of grassy cushions. These are truly “alpine” communities, found in the Apennines only at the summits of the highest massifs. This is the vegetation of the alpine tundra with *Silene acaulis* subsp. *bryoides* (Jord.) Nyman and *Saxifraga speciosa* Dörfl. & Hayek and the discontinuous grasslands with *Sesleria juncifolia* and *Carex kitaibeliana*. Finally, the most extreme mountain environments, represented by cliffs and loose debris, are home to highly specialised vegetation and flora. This is always “pioneer” and azonal vegetation. The cliffs are colonised by few species (mostly belonging to the *Potentilla* L. and *Saxifraga* Tourn. ex L. genera). The scree slopes allow the presence of at least three main types of vegetation, which alternate depending on the altitude and physical characteristics of the limestone scree: one association (the most common) is dominated by tall clumps of *Leucopoa dimorpha* (Guss.) H.Scholz & Foggi, while the other communities are distinguished by sparse vegetation cover, one characterised by *Isatis apennina* Ten. ex Grande and *Heracleum orsinii* Guss. and the other by *Drypis spinosa* L. subsp. *spinosa*.

### 2.2. Floristic Inventory

The floristic inventory is based on both field and bibliographic surveys. Field investigations were carried out from 1990 to 2025, following the established methodology for floristic research through 297 targeted explorations across the entire study area. Notably, during the last three years of this period (2023–2025), the field sampling became highly systematic thanks to dedicated funding provided by the park administration. Fieldwork also extended to areas immediately adjacent to the PRSV, based either on natural geographical limits or, in rare instances, driven by the presence of plants of considerable phytogeographical interest. To ensure a comprehensive checklist, the surveys systematically covered all macro- and micro-habitats present in the territory (including woodlands, grasslands, rocky outcrops, wetlands, and disturbed areas) across all characterising altitudinal belts. Furthermore, field sampling was repeated continuously (period 2023–2025) throughout all seasons to properly intercept the seasonal phenology of early-blooming, spring, summer, and late-autumn species.

Several herbaria were also consulted: APP, FI, NAP, RO (codes follow Thiers [46]), and Paolo Guarrera’s private herbarium. The specimens collected during our field investigations are kept at APP.

The collected plants were identified by the authors (FC and FB) using standard floras [47,48,49,50,51,52]. To identify taxa within critical groups, we consulted specific taxonomic studies, e.g., [53,54,55,56,57,58,59,60,61,62,63]. Some herbarium specimens belonging to critical genera were sent to specialists for revision: *Alchemilla* L. (F. Festi, Trento, B. Pierini, Pisa and G. Tondi, Rome), *Leucanthemum* Mill. (C. Oberprieler, Regensburg), *Orobanche* L. (G. Domina, Palermo), *Hieracium* L. and *Pilosella* Hill (G. Gottschlich, Tübingen).

A total of 11,150 floristic records from 250 scientific publications and 4136 herbarium specimens collected in the park’s territory have been included in a geo-database created ad hoc with File Maker Pro 8.5 (Claris International: Santa Clara, CA, USA) [64] integrated with QGIS (QGIS Association: Zurich, Switzerland) [65]. The selected literature encompasses all available and published scientific papers, monographs, and reports regarding floristic, phytosociological, taxonomic, and systematic studies explicitly carried out within or concerning the study area. This comprehensive bibliographic screening was performed to ensure that no historical or modern data regarding the local vascular flora were omitted from the geo-database.

The expected number of taxa (S) for both native and alien flora within the study area was calculated based on its total surface area (A = 550 km^2^) using the Arrhenius power function for species–area relationships: S = c A^Z^, where “c” and “z” represent coefficients empirically derived for the floristic richness of Italy by D’Antraccoli et al. [18]. Specifically, the values used were c = 245.2 and z = 0.263 for native taxa and c = 10.1 and z = 0.404 for alien taxa.

Nomenclature and taxonomic treatments were aligned with the two recently published checklists of the native [16] and alien [17] vascular flora of Italy, along with their subsequent updates [66,67,68,69,70] made available through the Portal to the Flora of Italy (https://dryades.units.it/floritaly/, accessed on 1 March 2026). Our delimitation of species and subspecies concepts is largely consistent with major international taxonomic databases, specifically Plants of the World Online [71], World Flora Online [72], and the Euro + Med PlantBase [73]. The Italian checklists deviate only marginally from these global databases; they represent the output of an extensive collaborative effort widely endorsed by both the national and international scientific communities. Consequently, they address fine-scale, nation-specific taxonomic issues, such as regional endemisms, with a higher degree of geographic and biological precision than global databases can typically provide.

Families are grouped into Lycophytes, ferns and fern-allied, Gymnosperms, and Angiosperms, and within these four groups, they are listed alphabetically.

The species and subspecies in the floristic list may be preceded by the following symbols:

E: Endemic to Italy (including Malta and Corsica) [16].

C: Cryptogenic (taxon doubtfully native) [16]: “C”.

Alien taxa [17,74]: A: archaeophyte; N: neophyte; AR: regional alien (native to Italy but alien to the administrative region (i.e., Abruzzo); AL: local alien (native to the administrative region (i.e., Abruzzo) but alien to the studied territory). Alien taxa are categorised as casual (CAS), naturalised (NAT), and invasive (INV).

#: Taxa occurring in the Monte Velino Nature Reserve.

*: New taxa (species and subspecies) for the PRSV’s flora.

Taxa doubtfully occurring (D, i.e., taxa reported in past literature but lacking herbarium specimens) and no longer recorded (NC, i.e., reliable historical record before 1965, usually based on herbarium specimens) follow the categorisation proposed by [16,75] and are indicated in the floristic list only in italics. The rarest species and subspecies or those of conservation interest are followed by distribution or nomenclature notes. For the common or less common species within the Park’s territory, we have added a note regarding the altitudinal belts in which the plant is distributed [hill and submontane belt (from 400 up to 1000/1100 m); montane belt (from 1000/1100 up to 1800/1900 m); subalpine belt (from 1800/1900 up to 2100/2200 m); alpine belt (above 2100/2200 m up to the peaks)].

The floristic list also includes conservation information relating to the inclusion of taxa in the Habitats Directive 92/43/EEC or in the Red List of Italian Flora [76,77,78].

Invasive alien species of Union concern are also indicated according to Regulation (EU) No 1143/2014 and Commission Implementing Regulations (EU) 2016/1141, 2017/1263, 2019/1262, and 2022/1203.

## 3. Results

### 3.1. Floristic Richness and Taxonomic Diversity

The complete floristic inventory of Sirente-Velino Regional Park is reported in Supplementary File S1. Based on the Arrhenius species–area relationship, the expected number of native and alien taxa is 1289 and 129, respectively (the values used were c = 245.2 and z = 0.263 for native taxa, and c = 10.1 and z = 0.404 for alien taxa [18]). The park’s flora consists of 2066 taxa (1370 species and 696 subspecies, including 11 hybrids), of which 2001 are established flora (i.e., native and cryptogenic + naturalised and invasive alien taxa), belonging to 117 families and 671 genera (Table 1).

**Figure 4:**
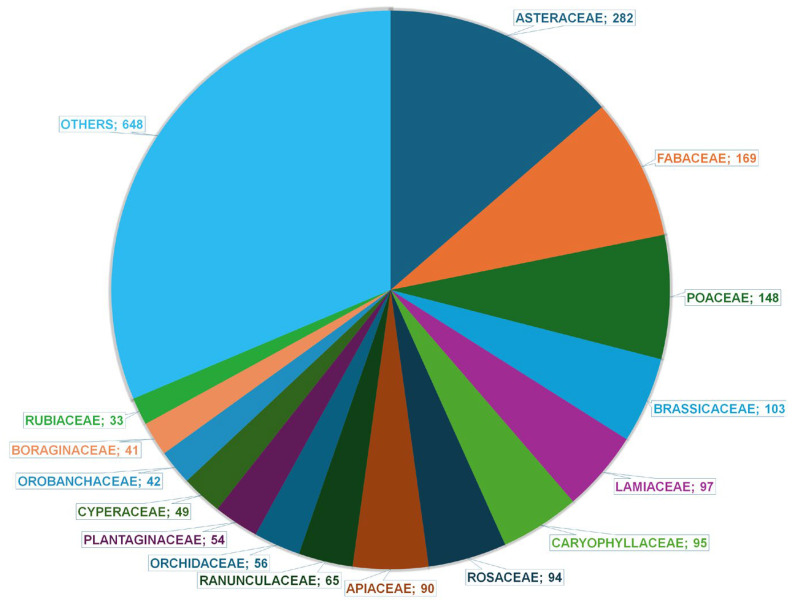
Taxonomic diversity within the flora of the Sirente-Velino Regional Park. Numbers indicate the count of identified taxa. Distribution by taxonomic family.

**Figure 5:**
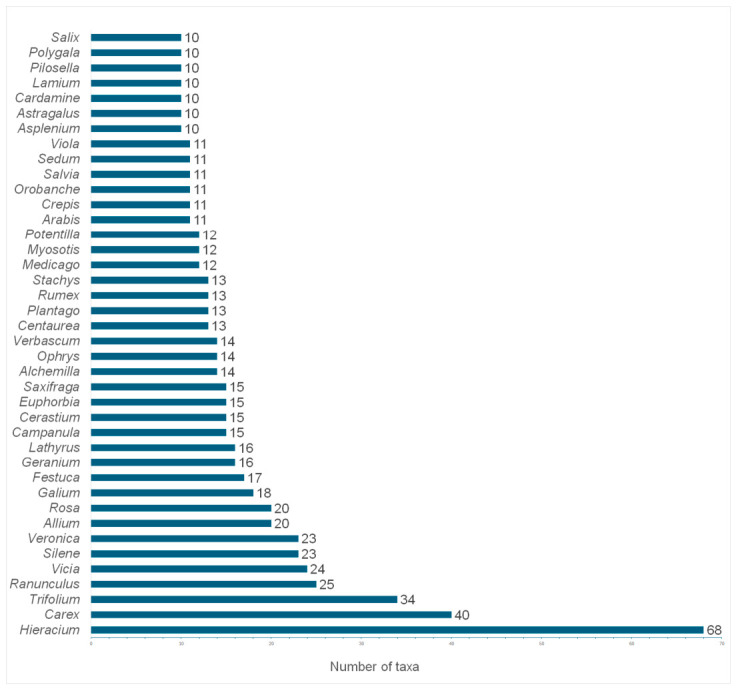
Taxonomic diversity within the flora of the Sirente-Velino Regional Park. Numbers indicate the count of identified taxa. Most species-rich genera (≥10 species and subspecies).

The ferns and fern allies are represented by 11 families, 13 genera and 35 species and subspecies. The gymnosperms are represented by four families, 11 genera and 18 species and subspecies. The angiosperms consist of 2013 species and subspecies grouped in 104 families and 647 genera. The most represented families (>100 species and subspecies) are Asteraceae (282), Fabaceae (169), Poaceae (148), and Brassicaceae (103) (Figure 4). The Orchidaceae family has 56 taxa (42 species and 14 subspecies), excluding hybrids, which have been deliberately excluded from this list. The richest genera (≥14 species and subspecies) are *Hieracium* L. (68), *Carex* L. (40), *Trifolium* Tourn. ex L. (34), *Ranunculus* L. (25), *Vicia* L. (24), *Silene* L. (23), *Veronica* L. (23), *Allium* L. (20), *Rosa* L. (19), *Festuca* Tourn. ex L. (17), *Galium* L. (17), *Geranium* Tourn. ex L. (16), *Lathyrus* L. (16), *Campanula* L. (15), *Cerastium* Tourn. ex L. (15), *Euphorbia* L. (15), and *Saxifraga* Tourn. ex L. (14) (Figure 5).

### 3.2. Endemic and Phytogeographically Significant Taxa

The Italian endemics in the study area are 169 taxa (Table 2). The taxa historically reported in the study area and not confirmed in recent times (i.e., not recorded after 1965) are 52, while 22 are doubtfully occurring.

Thanks to fieldwork, it has been possible to record 234 species and subspecies for the first time in the park, including five species, new or confirmed, to the regional flora: *Salsola tragus* L., *Hyacinthoides non-scripta* (L.) Chouard ex Rothm., *Alchemilla alpinula* S.E.Fröhner, *A. obtusa* Buser, and *A. vulgaris* L. Furthermore, *Euphorbia tommasiniana* Bertol. should be excluded from the flora of Abruzzo, as the revision of APP herbarium specimens indicates that the previous record of this species is actually attributable to the alien *E. saratoi* Ardoino.

Furthermore, after a critical review of the literature, careful analysis of some herbarium samples, and ad hoc field surveys, we can exclude 35 taxa from the Park’s flora that had been erroneously reported by various authors in the past (Supplementary Table S1).

### 3.3. Composition and Status of the Alien Flora

The list includes 13 cryptogenic and 122 alien plants (69 neophytes, 42 archaeophytes, five local aliens, and six regional aliens), of which 63 are casual, 48 are naturalised, and 11 are invasive, including one of EU relevance (*Ailanthus altissima* (Mill.) Swingle). In addition, two cultivated plants are used for reforestation: *Pinus mugo* Turra and *Alnus cordata* (Loisel.) Duby.

### 3.4. Conservation Status and Threats

A significant portion of the PRSV flora holds remarkable conservation value. Specifically, six taxa are listed in Annexes II and IV of the Habitats Directive 92/43/EEC, including two priority species. Additionally, 189 taxa are included in the Red Lists of the Italian Flora, with 18 classified into strictly threatened categories: four are Critically Endangered and 11 are Endangered. The primary contemporary pressures and threats identified across these vulnerable taxa include water abstraction and overgrazing for wetland species, climate change for high-altitude and rocky-inhabiting plants, and the encroachment of non-indigenous conifers from historical afforestation for continental steppe species.

## 4. Discussion

Italy ranks first in Europe for the number of vascular plant taxa [16,17,18]. Within the entire Mediterranean Basin, recognised as one of the world’s leading biodiversity hotspots, only Turkey hosts a higher number of species [79]. In this framework, the protected areas with the highest floristic diversity are located in the Central Apennines (i.e., Gran Sasso and Monti della Laga National Park, Maiella National Park, Abruzzo, Lazio and Molise National Park and PRSV), as a result of a complex interplay between historical biogeography, structural geology, and contemporary ecological gradients. Situated at the very heart of the Mediterranean, these territories serve as contact zones for eastern and western floras. The Sirente-Velino Regional Park stands out as one of the most species-rich protected areas in Europe (Table 3), hosting 20.6% of the entire Italian flora.

Our results demonstrate that native species richness in the studied area is 50.8% higher than modelled expectations, while alien species richness is 5.6% lower [18]. This divergence can be attributed to substantial environmental variability; the park’s complex geographical layout, diverse geological substrates, and microclimatic gradients create numerous specialised ecological niches. Concurrently, the high ecological integrity of the area—maintained by minimal human impact, a mountainous landscape, and a sparse human population—limits the establishment of alien taxa.

**Figure 6:**
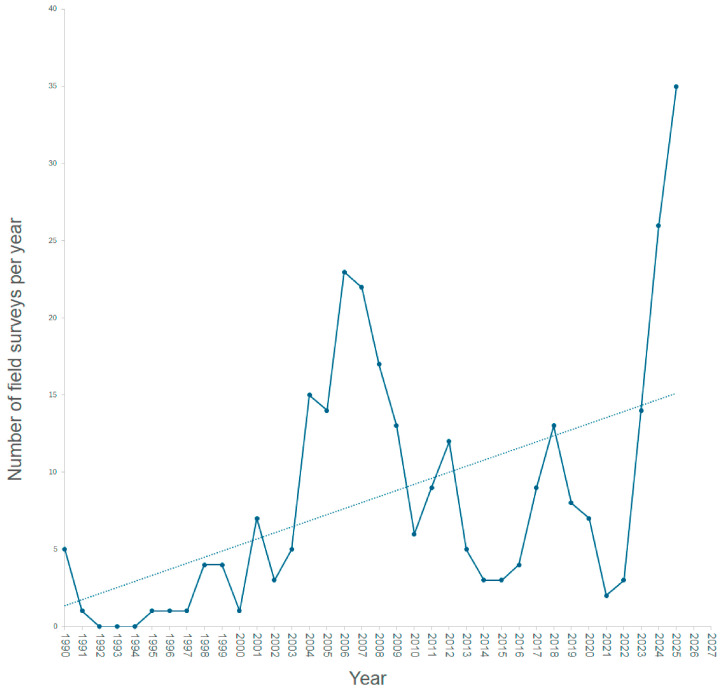
Temporal distribution of floristic field surveys carried out in the Sirente-Velino Regional Park from 1990 to 2025 (N = 297). The solid line with markers indicates the annual number of field surveys, while the dashed line represents the overall linear trend.

Over the entire study period (1990–2025), a total of 297 field surveys were carried out within the regional park. The temporal distribution of these surveys shows a dynamic and overall positive trend, as highlighted by the linear regression analysis (Figure 6).

In the first decade (1990–2000), floristic exploration remained sporadic, characterised by a low baseline of activity with fewer than five surveys per year and a complete absence of fieldwork between 1992 and 1994. A first significant peak of field activity occurred between 2004 and 2008, reaching its maximum in 2006 with 23 surveys, which corresponds to specific taxonomic and mapping projects.

Following a sharp decline and a subsequent fluctuating plateau during the 2010s, likely due to localised sampling gaps, field exploration experienced an unprecedented and exponential surge in the last three years (2023–2025). This recent effort culminated in 2025 with 35 surveys, representing the highest annual field intensity ever recorded for the study area. This strong final acceleration heavily drives the overall upward trend, reflecting a renewed and systematic effort to finalise the modern vascular flora inventory of the park.

All the floristic and nomenclatural information gathered during these intensive field campaigns, alongside historical literature and herbarium data, has been systematically integrated into an ad hoc-created geo-database. This digital repository does not merely represent a static inventory but serves as a dynamic tool designed to support long-term floristic monitoring, biogeographical analyses, and concrete conservation planning within the park. By making these comprehensive datasets available upon reasonable request, this work establishes a baseline framework intended to grow continuously as new records emerge, providing a solid foundation for a future, open-access public repository of the park’s vascular flora.

Over years of extensive field explorations within the PRSV, numerous floristic discoveries of outstanding phytogeographical significance have been made, underscoring the park’s critical role as a biodiversity hotspot. Notably, these investigations led to the identification of several taxa new to the Italian flora, including *Gagea luberonensis* J.-M.Tison [106], *Jacobaea vulgaris* Gaertn. subsp. *gotlandica* (Neuman) B.Nord. [107], and *Klasea lycopifolia* (Vill.) Á.Löve & D.Löve [108]. Furthermore, *Allium angulosum* L. [109] and *Gagea ramulosa* A.Terracc. [106] were recorded for the first time on the Italian peninsula. Furthermore, the park yielded significant records for the Apennines and central Italy, such as *Artemisia atrata* Lam. [110], *Carex hartmaniorum* A.Cajander [66], and *Myosotis minutiflora* Boiss. & Reut. subsp. *minutiflora* [27], as new to the Apennines, alongside *Barbarea sicula* C.Presl [27], new to the central Apennines, and *Galeopsis bifida* Boenn. [22], new to central Italy. In terms of chorological boundaries and rare occurrences, field surveys redefined the distribution of *Seseli pallasii* Besser, marking its new southern range limit in Italy [66], and the first record for the central Apennines of *Rumex thyrsiflorus* Fingerh., confirmed as native in Italy, exclusively to the Abruzzo region [22]. Similarly, *Juncus atratus* Krock. represents a remarkable finding, having been previously confirmed in Italy only within the Umbria region [27,111]. Finally, the park shelters highly localised relics such as *Spiraea hypericifolia* L. subsp. *hypericifolia*, a steppe plant of exceptional phytogeographical interest known in Italy only from restricted areas around the Fucino plain and in the L’Aquila basin (Abruzzo), with historical reports from Umbria remaining unconfirmed since the nineteenth century [112].

Additional records have recently been published for the Abruzzo region [113]: *Hedera algeriensis* Rantonnet ex C.Morren (casual alien), *Leucanthemum* × *superbum* (Bergmans ex J.W.Ingram) D.H.Kent (casual alien), *Nymphaea* × *marliacea* Lat.-Marl. (casual alien), *Triticum* × *requienii* Ces., Pass. & Gibelli nothosubsp. *requienii* (casual alien), and *Yucca filamentosa* (casual alien). Thanks to the review of herbarium material, it was also possible to correctly identify the Apennine populations of *Hypericum richeri* Vill., which were attributed to *H. richeri* subsp. *grisebachii* (Boiss.) Nyman, new to Italy [113].

In addition, six taxa reported for the PRSV territory have recently been excluded from the Abruzzo region [113]: *Aquilegia atrata* W.D.J.Koch, *Corydalis intermedia* (L.) Mérat, *Euphorbia seguieriana* Neck. subsp. *seguieriana*, *Onosma pseudoarenaria* Schur subsp. *helvetica* (Nyman) Rauschert, *Potentilla grandiflora* L., and *Ranunculus circinatus* Sibth.

Furthermore, a taxonomic revision of the herbarium specimens collected within the PRSV and deposited at APP, following the taxonomic information by [57], indicates that *Euphorbia tommasiniana* Bertol., originally reported for only a single locality in the region [114], must be excluded from the Abruzzo flora. These specimens are instead attributable to the alien species *E. saratoi* Ardoino. *Euphorbia tommasiniana* is strictly endemic to NE Italy [57], and the records of this taxon for the Italian Peninsula should be revised and are probably, in part, referable to *E. saratoi* [66].

**Figure 7:**
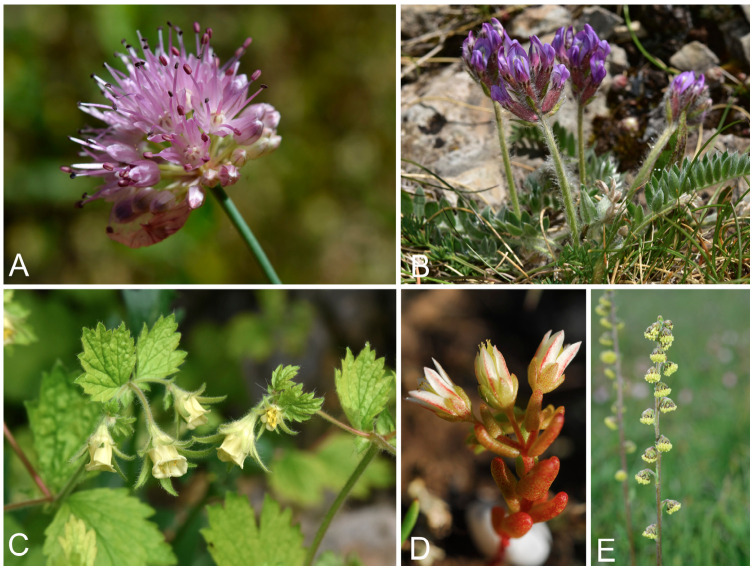
(**A**): *Allium ducissae* Bartolucci, Iocchi & F.Conti (photo by F. Bartolucci); (**B**): *Oxytropis ocrensis* F.Conti & Bartolucci (photo by F. Bartolucci); (**C**): *Geum heterocarpum* Boiss. from Mt. Briccialone (photo by F. Bartolucci); (**D**): *Sedum aquilanum* L.Gallo & F.Conti & Bartolucci (photo by F. Conti); (**E**): *Artemisia atrata* Lam. (photo by F. Conti).

Among the Italian endemics, 45 taxa are restricted to central Italy (Apennine), 24 taxa to the Abruzzo region, and four taxa to the PRSV and surrounding areas (Table 2). Three species have recently been described based on material collected in the PRSV: *Allium ducissae* Bartolucci, Iocchi & F.Conti from Mt. Rozza (Figure 7A) [115], *Oxytropis ocrensis* F.Conti & Bartolucci from Mt. Ocre (Figure 7B) [116], and *Sedum aquilanum* L.Gallo & F.Conti from Campo Felice (Figure 7D) [117].

From a phytogeographical perspective, the PRSV hosts 15 specific and subspecific taxa with a disjointed distribution range, with the only known Italian localities of occurrence in central Apennine (*Allium permixtum* Guss., *Androsace maxima* L., *Colchicum bulbocodium* subsp. *versicolor* (Ker Gawl.) K.Perss., *Juncus atratus* Krock., *Klasea lycopifolia* (Vill.) Á.Löve & D.Löve, *Malcolmia orsiniana* (Ten.) Ten. subsp. *orsiniana*, *Spiraea hypericifolia* L. subsp. *hypericifolia*), in the Abruzzo region (*Adonis vernalis* L., *Jacobaea vulgaris* subsp. *gotlandica* (Neuman) B.Nord., *Orlaya daucorlaya* Murb., *Geum heterocarpum* Boiss. (Figure 7C), *Pedicularis friderici-augusti* Tomm., *Thymus zygiformis* Heinr.Braun), or in the PRSV and surrounding areas (*Artemisia atrata* Lam. (Figure 7E) and *Gagea ramulosa* A.Terracc. [considering peninsular Italy]). The only Italian population of *Geum heterocarpum* (about 50 specimens) was recently re-discovered in the study area [118]. The conservation of taxa with disjunct distribution ranges is crucial, as these isolated populations not only represent unique evolutionary lineages shaped by restricted gene flow, but also provide vital phytogeographical insights into past climatic shifts and glacial refugia dynamics [119,120].

A total of 52 taxa historically reported in the study area could not be confirmed in recent times. Although targeted searches were conducted, some of these taxa may have been overlooked, given their documented presence in neighbouring areas of the Central Apennines. Consequently, further dedicated surveys are warranted to clarify their status.

Among the flora of conservation interest, six taxa are listed in Annexes II and IV of the Habitats Directive 92/43/EEC: *Adonis distorta* Ten., *Astragalus aquilanus* Anzal. (priority), *Himantoglossum adriaticum* H.Baumann, *Iris marsica* I.Ricci & Colas., *Jacobaea vulgaris* subsp. *gotlandica*, and *Klasea lycopifolia* (priority). Field monitoring data for these target taxa were collected during our surveys and integrated with the outcomes of the LIFE15 NAT/IT/000946 FLORANET project, which aimed to enhance their conservation status via in situ and ex situ actions. These data directly supported the drafting of both the fourth [121] and the recently finalised fifth reporting cycles under Article 17 of the Habitats Directive.

**Figure 8:**
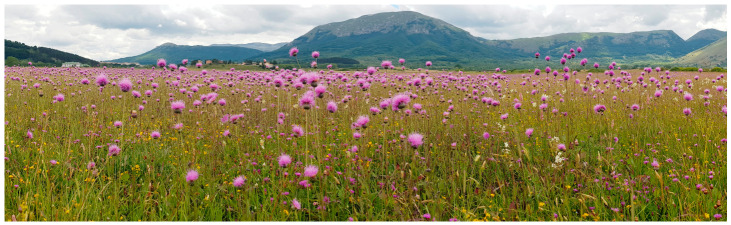
*Klasea lycopifolia* blooming on the Altopiano delle Rocche. This plant community is referable to the *Lathyro asphodeloidis-Klaseetum lycopifoliae* association (photo by F. Bartolucci).

The preservation of one of these species is strictly linked to the park’s karstic plains, which represent areas of utmost conservation importance. Remarkably, these plains host unique grassland communities dominated by *K. lycopifolia*, a species exceptionally rare in the Italian peninsula. Formally described [44] as a new plant association (*Lathyro asphodeloidis-Klaseetum lycopifoliae*, alliance *Cynosurion cristati*), these coenoses underscore the vital role of the PRSV in sheltering rare and threatened ecosystems (Figure 8).

A total of 189 taxa within the park are included in the Red Lists of the Italian Flora [76,77,78,122]. Among these, 18 taxa fall into threatened categories: four are assessed as Critically Endangered (*Geum heterocarpum*, *Juncus atratus*, *Rosa stylosa* Desv., and *Sedum aquilanum*), 11 as Endangered (*Adonis distorta*, *A. vernalis*, *Artemisia atrata* Lam., *Astragalus aquilanus*, *Carex vulpina* L., *Dianthus guliae* Janka, *Goniolimon tataricum* (L.) Boiss. subsp. *italicum* (Tammaro, Pignatti & Frizzi) Buzurović, *Pinguicula vulgaris* L. subsp. *vestina* F.Conti & Peruzzi, *Salvia aethiopis* L., *Spiraea hypericifolia* subsp. *hypericifolia*, and *Viola kitaibeliana* Schult.), and three as Vulnerable (*Allium permixtum*, *Gentiana pneumonanthe* L. subsp. *pneumonanthe*, and *Oxytropis ocrensis*), whereas 27 are listed as Data Deficient. Conversely, the remaining 14 Near Threatened (NT) and 130 Least Concern (LC) taxa do not currently exhibit any significant conservation issues.

Specific pressures and threats affect the threatened categories depending on their habitat preferences. Specifically, wetland species (i.e., *Allium permixtum*, *Carex vulpina*, *Gentiana pneumonanthe* subsp. *pneumonanthe*, *Juncus atratus*, *Pinguicula vulgaris* subsp. *vestina*, and *Sedum aquilanum*) are mostly threatened by water abstraction and the consequent lowering of the water table, as well as localised overgrazing; furthermore, future risks may arise from ski resort expansions. For species inhabiting rocky sectors or high-altitude areas (e.g., *Adonis distorta*, *Geum heterocarpum*, and *Oxytropis ocrensis*), climate change constitutes the main long-term threat. Finally, continental steppe species (e.g., *Adonis vernalis* and *Goniolimon tataricum* subsp. *italicum*) suffer from pressures related to the expansion and invasiveness of alien or non-indigenous conifers used for historical afforestation, particularly *Pinus nigra* J.F.Arnold subsp. *nigra*.

The total number of established alien taxa [NAT (48) + INV (11)] occurring in the study area is quite low and reflects a good conservation status of the Park’s territory and a good integrity of the natural and semi-natural habitats. Furthermore, among the alien taxa, only *Ailanthus altissima* (Mill.) Swingle is included in the list of invasive alien species of Union concern under Regulation (EU) No 1143/2014 and Commission Implementing Regulations (EU) 2016/1141, 2017/1263, 2019/1262, and 2022/1203. Although this species is relatively abundant within the study area, its distribution is strictly confined to synanthropic habitats, such as the immediate vicinity of human settlements and roadside margins. Consequently, designing and implementing an effective eradication strategy would present significant logistical challenges. The situation is different for *Senecio inaequidens* DC., which is not yet widespread in the study area and remains mostly localised along roadsides. In this case, immediate intervention could prevent the spread of this species, which in other Italian and European contexts has proven capable of invading natural environments, significantly impacting plant biodiversity and habitats of high conservation value [123,124]. Furthermore, we found a few individuals of *S. inaequidens* at an elevation of 1900 m a.s.l. near a ski lift terminus, representing the highest altitude recorded for this species to date in the Apennines.

Finally, it is important to note that genera such as *Hieracium* and *Pilosella* are characterised by high taxonomic complexity and instability [125]. Although the preparation of this flora strictly followed the taxonomic treatment currently recognised for Italian flora [16], future reassessments may be necessary as new molecular or integrative taxonomic evidence becomes available.

## 5. Conclusions

The first comprehensive and updated vascular flora of the Sirente-Velino Regional Park reveals an extraordinary botanical heritage, given its limited extension, accounting for 2066 taxa, which represents approximately 20.6% of the entire Italian flora. This remarkable richness underscores the park’s status as a major plant biodiversity hotspot of European significance. The divergence from theoretical macro-regional models—exhibited by a 50.8% increase in native species richness and a 5.6% reduction in alien taxa—highlights the exceptional ecological integrity and environmental heterogeneity of this territory. This pattern is primarily driven by the region’s complex geographical layout, high geological and mesoclimatic diversity, and low degree of human disturbance across its mountainous landscapes.

The high conservation value of the study area is further emphasised by the presence of 169 taxa endemic to Italy, several species of European concern listed in the Habitats Directive, and 189 red-listed taxa, including four Critically Endangered species (i.e., *Geum heterocarpum*, *Juncus atratus*, *Rosa stylosa*, and *Sedum aquilanum*). Furthermore, the documentation of unique, isolated populations with disjunct distribution ranges (e.g., *Adonis vernalis*, *Artemisia atrata*, *Jacobaea vulgaris* subsp. *gotlandica*, *Gagea ramulosa*, *Geum heterocarpum*, *Pedicularis friderici-augusti*, *Thymus zygiformis*) provides vital insights into past climatic shifts and underscores the park’s role as an evolutionary and glacial refugium.

From a conservation and management perspective, this rigorous floristic inventory provides a fundamental baseline tool for the Park Authority. It enables decision-makers to delineate clear conservation priorities and implement targeted, science-based strategies to safeguard fragile ecosystems and endangered species. Crucially, while the overall impact of alien flora remains low, the documentation of early-stage invasions by non-native species such as *Senecio inaequidens* at high altitudes stresses the urgent need for active monitoring and timely eradication protocols. Ultimately, this baseline study will support ongoing reporting obligations under the Habitats Directive (Article 17) and guide future long-term monitoring networks to combat biodiversity loss in the face of ongoing environmental and climate changes.

## Figures and Tables

**Table 1:** Number of species and subspecies listed in the floristic inventory of the PRSV. P (present), NC (not confirmed, i.e., not recorded after 1965), D (doubtfully occurring), NP (not present, i.e., erroneously reported).

	P	NC	D	NP	Total
Native	1856	51	22	34	1929
Cryptogenic	12	1			13
Invasive alien	11				11
Naturalised alien	48			1	48
Casual alien	63				63
Cultivated	2				2
Total	1992	52	22	35	2066

**Table 2:** Italian endemics with limited distribution recorded in the Sirente-Velino Regional Park (PRSV).

Taxa (Species and Subspecies)	Italian Distribution
*Achillea barrelieri* (Ten.) Sch.Bip. subsp. *barrelieri*	Endemic to C Italy
*Adonis distorta* Ten.	Endemic to C Italy
*Alchemilla marsica* Buser	Endemic to C Italy
*Allium ducissae* Bartolucci, Iocchi & F.Conti	Endemic to PRSV and surrounding areas
*Androsace vitaliana* (L.) Lapeyr. subsp. *praetutiana* (Buser ex Sünd.) Kress	Endemic to C Italy
*Anthyllis apennina* F.Conti & Bartolucci	Endemic to C Italy
*Anthyllis vulneraria* L. subsp. *nana* (Ten.) Tammaro	Endemic to C Italy
*Astrantia pauciflora* Bertol. subsp. *tenorei* (Mariotti) Bechi & Garbari	Endemic to C Italy
*Betonica alopecuros* L. subsp. *divulsa* (Ten.) Bartolucci & Peruzzi	Endemic to C Italy
*Biscutella laevigata* L. subsp. *australis* Raffaelli & Baldoin	Endemic to C Italy
*Campanula fragilis* Cirillo subsp. *cavolinii* (Ten.) Damboldt	Endemic to C Italy
*Centaurea ambigua* Guss. subsp. *ambigua*	Endemic to C Italy
*Centaurea ambigua* Guss. subsp. *nigra* (Fiori) Pignatti	Endemic to C Italy
*Centaurea ceratophylla* Ten. subsp. *ceratophylla*	Endemic to C Italy
*Cerastium thomasii* Ten.	Endemic to C Italy
*Coristospermum cuneifolium* (Guss.) Bertol.	Endemic to C Italy
*Cymbalaria glutinosa* Bigazzi & Raffaelli subsp. *glutinosa*	Endemic to C Italy
*Cymbalaria pallida* (Ten.) Wettst.	Endemic to C Italy
*Cynanchica pyrenaica* (L.) P.Caputo & Del Guacchio subsp. *neglecta* (Guss.) P.Caputo & Del Guacchio	Endemic to C Italy
*Erodium alpinum* (Burm.f.) L’Hér.	Endemic to C Italy
*Erysimum majellense* Polatschek	Endemic to C Italy
*Euphorbia gasparrinii* Boiss. subsp. *samnitica* (Fiori) Pignatti	Endemic to C Italy
*Festuca imperatrix* Catonica	Endemic to Abruzzo
*Goniolimon tataricum* (L.) Boiss. subsp. *italicum* (Tammaro, Pignatti & Frizzi) Buzurović	Endemic to Abruzzo
*Herniaria bornmuelleri* Chaudhri	Endemic to Abruzzo
*Hieracium bifidum* Kit. ex Hornem. subsp. *nummulariifolium* Gottschl.	Endemic to Abruzzo
*Hieracium bifidum* Kit. ex Hornem. subsp. *subhastatum* Gottschl.	Endemic to Abruzzo
*Hieracium bifidum* Kit. ex Hornem. subsp. *subimbricatum* Gottschl.	Endemic to C Italy
*Hieracium bupleuroides* C.C.Gmel. subsp. *aprutiorum* (Furrer & Zahn) Gottschl.	Endemic to Abruzzo
*Hieracium chlorifolium* Arv.-Touv. subsp. *rendinaricum* Gottschl.	Endemic to C Italy
*Hieracium contii* Gottschl.	Endemic to Abruzzo
*Hieracium dentatum* Hoppe subsp. *trefferianiforme* Gottschl.	Endemic to Abruzzo
*Hieracium galeroides* Gottschl. subsp. *galeroides*	Endemic to Abruzzo
*Hieracium hypochoeroides* S.Gibson subsp. *bifidopsis* (Zahn) Greuter	Endemic to C Italy
*Hieracium hypochoeroides* S.Gibson subsp. *pallidopsis* Gottschl.	Endemic to C Italy
*Hieracium hypochoeroides* S.Gibson subsp. *potamogetifolium* Gottschl.	Endemic to Abruzzo
*Hieracium lycopifolium* Froel. subsp. *ocreanum* Gottschl.	Endemic to the PRSV
*Hieracium neoplatyphyllum* Gottschl. subsp. *malacofloccosum* Gottschl.	Endemic to Abruzzo
*Hieracium pallescens* Waldst. & Kit. subsp. *ciliatifolium* (Zahn) Gottschl.	Endemic to Abruzzo
*Hieracium permaculatum* Gottschl. subsp. *permaculatum*	Endemic to Abruzzo
*Hieracium pietrae* Zahn	Endemic to C Italy
*Hieracium prenanthoides* Vill. subsp. *lissocorium* Furrer & Zahn	Endemic to the PRSV
*Hieracium prenanthoides* Vill. subsp. *stupposifolium* Gottschl.	Endemic to Abruzzo
*Hieracium racemosum* Waldst. & Kit. ex Willd. subsp. *caramanicum* (Zahn) Zahn	Endemic to Abruzzo
*Hieracium racemosum* Waldst. & Kit. ex Willd. subsp. *pulmonariifolium* Gottschl.	Endemic to Abruzzo
*Hieracium venticaesum* Gottschl.	Endemic to Abruzzo
*Hieracium villosum* Jacq. subsp. *doratophyllum* Nägeli & Peter	Endemic to Abruzzo
*Iris marsica* I.Ricci & Colas.	Endemic to C Italy
*Minuartia glomerata* (M.Bieb.) Degen subsp. *trichocalycina* (Ten. & Guss.) F.Conti	Endemic to C Italy
*Noccaea* stylosa (Ten.) Rchb.	Endemic to C Italy
*Ononis cristata* Mill. subsp. *apennina* Tammaro & Catonica	Endemic to C Italy
*Oxytropis ocrensis* F.Conti & Bartolucci	Endemic to Abruzzo
*Paeonia officinalis* L. subsp. *italica* N.G.Passal. & Bernardo	Endemic to C Italy
*Phyllolepidum rupestre* (Sweet) Trinajstić	Endemic to Abruzzo
*Pilosella cepitina* (Gottschl.) Gottschl.	Endemic to Abruzzo
*Pinguicula vulgaris* L. subsp. *vestina* F.Conti & Peruzzi	Endemic to Abruzzo
*Polygala alpestris* Rchb. subsp. *angelisii* (Ten.) Nyman	Endemic to Abruzzo
*Ranunculus magellensis* Ten.	Endemic to C Italy
*Ranunculus marsicus* Guss. & Ten.	Endemic to C Italy
*Saxifraga exarata* Vill. subsp. *ampullacea* (Ten.) D.A.Webb	Endemic to C Italy
*Saxifraga italica* D.A.Webb	Endemic to C Italy
*Saxifraga speciosa* Dörfl. & Hayek	Endemic to C Italy
*Scorzoneroides* montana (Lam.) Holub subsp. *breviscapa* (DC.) Greuter	Endemic to C Italy
*Sedum aquilanum* L.Gallo & F.Conti	Endemic to the PRSV
*Senecio apenninus* Tausch	Endemic to C Italy
*Senecio tenorei* Pignatti	Endemic to C Italy
*Sempervivum riccii* Iberite & Anzal.	Endemic to C Italy
*Silene cattariniana* Ferrarini & Cecchi	Endemic to C Italy
*Silene notarisii* Ces.	Endemic to C Italy
*Taraxacum vaccarii* Soest	Endemic to C Italy
*Trisetum bertolonii* Jonsell	Endemic to C Italy
*Viola eugeniae* Parl. subsp. *levieri* (Parl.) Arcang.	Endemic to C Italy
*Achillea barrelieri* (Ten.) Sch.Bip. subsp. *barrelieri*	Endemic to Abruzzo

**Table 3:** Number of vascular plant taxa (species and subspecies), native and alien, in the most species-rich Mediterranean and European protected areas.

Protected Areas	Country	Surface Area (ha)	Taxa (Species and Subspecies)	References
Gran Sasso-Laga National Park	Italy	141,300	2678	[19,20,21,22]
Mercantour National Park	France	146,500	2405	[80]
Majella National Park	Italy	74,095	2335	[23,24,25]
Cevennes National Park	France	372,991	2216	[81]
Abruzzo, Lazio and Molise National Park	Italy	104,000	2116	[21,22,26,27,28]
Sirente-Velino Regional Park	Italy	55,000	2066	This paper
Olympus National Park	Greece	37,400	1983	[82]
Cilento National Park	Italy	181,048	1942	[83]
Sibillini National Park	Italy	69,722	1920	[84]
Dolomiti Bellunesi National Park	Italy	31,034	1685	[85]
Picos de Europa National Park	Spain	64,660	1673	[86]
Krka National Park	Croatia	14,200	1509	[87]
Foreste Casentinesi National Park	Italy	36,400	1415	[88]
Monfragüe National Park	Spain	150,000	1404	[89]
Ordesa y Monte Perdido National Park	Spain	15,608	1394	[90]
Sierra de las Nieves National Park	Spain	30,000	1387	[91]
Prespa (Grecia) National Park	Greece	25,600	1326	[92]
Uludag National Park	Turkey	12,762	1309	[93]
Hohe Tauern National Park	Austria	185,600	1286	
Plitvice National Park	Croatia	29,482	1267	[94]
Circeo National Park	Italy	8400	1245	[95]
Gran Paradiso National Park	Italy	71,040	1172	[96]
Risnjak National Park	Croatia	6410	1167	[97]
Mt. Oiti National Park	Greece	7210	1153	[98]
El-Kala National Park	Algeria	76,438	1050	[99]
Karagöl-Sahara National Park	Turkey	21,912	872	[100]
Meshchera National Park	Russia	118,760	812	[101]
Paklenica National Park	Croatia	10,200	809	[102]
Hatila Valley National Park	Turkey	25,000	769	[103]
Djurdjura National Park	Algeria	18,550	757	[104]
Zakynthos National Marine Park	Greece	4500	559	[105]

## Data Availability

The complete floristic inventory and information about floristic records are available in the Supplementary Materials. The data included in the geo-database are available from the corresponding authors upon reasonable request.

## Supplementary Materials

The following supporting information can be downloaded at: https://www.mdpi.com/article/10.3390/biology15131093/s1. File S1: Floristic Inventory. Table S1: Taxa (species and subspecies) to be excluded from the flora of Sirente-Velino Regional Park.
