# Supplementary material for: The Vascular Flora of Sirente-Velino Regional Park (Abruzzo, Central Italy)
Source: Biology (Basel). 2026 Jul 7;15(13):1093. doi: 10.3390/biology15131093 (PMC13359650; doi:10.3390/biology15131093)
Supplement: Supplementary file 1 [file biology-15-01093-s001.zip › Sup. file_Table S1.pdf]

Supplementay file_TabS1

Table S1. Taxa (species and subspecies) to be excluded from the flora of Sirente-Velino Regional Park (PRSV). Taxa reported for the park but already erroneously indicated for Abruzzo according to Bartolucci et al. (2024) and Galasso et al. (2024) are not included here.

Taxa (species and subspecies) to be excluded	Notes
Allium ochroleucum	The report from Castelvecchio Subequo (Groves 1880) is unlikely because the species prefers higher altitudes.
Armeria arenaria	This report by Groves (1880) is to be referred to *A. gracilis* (Tiburtini et al. 2022)
Bellis annua subsp. *annua*	*Bellis anna* is a mediterranean species, and the report by Tammaro et al. (1974) is probably due to confusion with poorly developed individuals of *Bellis perennis*. In fact, the species has not been confirmed since Guarrera & Tammaro (1996)
Betonica hirsuta	The report by Martelli (1904) is probably to be referred to *Betonica officinalis* var. *serotina*.
Campanula rotundifolia	The reports by Tenore (1830, 1831) and Montelucci should be referred to cloe taxa as *C. micrantha* and *C. tanfanii*.
Crepis paludosa	Examination of the herbarium specimens of *C. paludosa* in the Guarrera herbarium (Frizzi et al. 1996, Guarrera & Tammaro 1996) allowed us to attribute them to *Hieracium amplexicaule* subsp. *berardianum* (determination confirmed by G. Gottschlich).
Dactylorhiza maculata subsp. *fuchsii*	The populations of Sirente Velino Regional Park (Guarrera & Tammaro 1996) should be referred to *D. gervasiana*.
Draba aspera	The report by Groves (1880 sub *D. cuspidata*) should be referred to *D. aizoides*.
Echinops siculus	The report by Frizzi et al. (1996) and Guarrera & Tammaro (1996) is unlikely because it is a species that is more commonly found in the Mediterranean habitats. In the locations indicated (near Secinaro and Gole di Celano), we only found *E. sphaerocephalus* subsp. *sphaerocephalus*.
Erigeron strigosus	The report by Conti et al. (2023 sub *E. annuus* subsp. *strigosus*) from Molina Aterno is to be referred to a *E. annuus* as correctly reported by Conti et al. (2008).
Erodium moschatum	The report by Guarrera & Tammaro (1996) from Aielli is to be referred to *E. ciconium* based on the revision of specimens kept at Guarrera herbarium.
Euphorbia esula subsp. *tommasiniana*	The report by Conti et al. (2008) is to be referred to *E. saratoi*.
Euphorbia rigida	The report by Tenore (1831) is to be referred to *E. myrsinites*
Euphorbia verrucosa	The report by Guarrera & Tammaro (1996) is to be referred to *E. gasparrinii* subsp. *samnitica* (Cresti et al. 2019).
Ferula communis subsp. *communis*	The reports of *F. communis* for Rocca di Cambio (Anzalone et al. 1992) are implausible, while those for Gole di Celano (Anzalone

	et al. 1992, Frizzi et al. 1996) and the Gole di S. Venanzio (Lastoria 2000) are attributable to *F. glauca*, which has already been reported for the these localities.
Gentianella campestris subsp. *campestris*	The report for Colle dell'Orso (Rocca di Mezzo) by Balsi et al. (1992) is to be referred to *G. columnae*.
Glebionis segetum	The report for Velino massid (Petriccione 1993) is implausible
Iberis sempervirens	The report for Gole di San Venanzio (Conti 1993) is incorrect; it refers to an atypical population found at low altitude and should be attributed to *I. saxatilis*.
Laburnum alpinum	This report (Petriccione 1993) should be referred to *L. anagyroides* subsp. *anagyroides*.
Luzula sylvatica subsp. *sicula*	Reported by Petriccione (1993, 1994, 2005). All specimens observed and collected in the central Apennines are to be attributed as *L. sylvatica* subsp. *sieberi*.
Mcneillia rosanoi subsp. *clandestina*	The reports by Groves (1880) and Tammaro et al. (1974)are to be referred to M. rosanoi subsp. rosanoi, the only taxon occurring in the Sirente Velino Regional Park.
Myosotis sylvatica subsp. *sylvatica*	The reports by Veri & Tammaro (1980), Lucchese & Lattanzi (1993) and Petriccione (1993) probably refer to *M. sylvatic*a subsp. *cyanea*, the only subspecies confirmed by us throughout the Abruzzo region.
Onobrychis montana	The report by Martelli (1904 sub *O. conferta* Ten.) is unlikely, as *O. montana* is considered to be of doubtful presence and was never found during field surveys.
Poa badensis	The report by Montelucci (1958) should be referred to *P. molinerii*.
Pseudorchis albida	Recorded by Pezzetta (2016) on a bibliographic basis but not actually cited in any publication and not observed by any botanist.
Pulsatilla montana subsp. *montana*	The report by Guarrera & Tammaro (1996) is to be referred to *P. alpina* subsp. *millefoliata* after revision of specimens kept at Guarrera herbarium
Ranunculus polyanthemoides	The reports for the sudy area (Lucchese & Lattanzi 1994; Guarrera & Tammaro 1996; Ciaschetti & Pirone 2019) are to be referred to other taxa within the *R. polyanthemos* species complex.
Ribes petraeum	Recorded by Veri & Tammaro (1980) and Pirone (1995). This species is not present in Abruzzo and is probably to be referred to *R. alpinum*.
Saxifraga oppositifolia	The reports of this species in the PRSV (i.e. Tenore 1830, Groves 1880) and in the central Apennine are to be referred to *S. speciosa*
Seseli annuum subsp. *annuum*	The report by Conti et al. (2025) is to be referred to S. pallasii (Bartolucci et al. 2025).
Thymus oenipontanus	The reports by Veri & Tammaro (1980) and Guarrera & Tammaro (1996) after herbarium revisions and field investigations are to be referred to *T. moesiacus*.
Valeriana dioica	Recorded by Frizzi et al. (1996) and Guarrera & Tammaro (1996). Examination of specimens in the Guarrera herbarium allowed us to attribute them to *V. tuberosa*.
Veronica barrelieri subsp. *barrelieri*	The reports by Lucchese & Lattanzi (199e) and Guarrera & Tammaro (1996) are to be referred to *V. spicata* subsp. *fischeri*.

Vicia narbonensis	The report by Guarrera & Tammaro (1996) is to be referred to *V. johannis*.
Viola hirta	Reported for Prati del Sirente by Tammaro et al. (1974) but not confirmed in Guarrera & Tammmaro (1996).
Ziziphora suaveolens	The repots for Velino Massif (Lucchese & Lattanzi 1993) is unlikely and not confirmed by our field and herbarium observations.

References

Anzalone B., Lattanzi E., Leporatti M.L., 1992 - Il gruppo di Ferula communis L. (Umbelliferae) in Italia: ricerche sistematiche e corologiche. Archivio Botanico e Biogeografico Italiano, 67 (3-4) (1991): 221-236.

Bartolucci F., Peruzzi L., Galasso G., Alessandrini A., Ardenghi N.M.G., Bacchetta G., Banfi E., Barberis G., Bernardo L., Bouvet D., Bovio M., Calvia G., Castello M., Cecchi L., Del Guacchio E., Domina G., Fascetti S., Gallo L., Gottschlich G., Guarino R., Gubellini L., Hofmann N., Iberite M., Jimenez-Mejias P., Longo D., Marchetti D., Martini F., Masin R.R., Medagli P., Peccenini S., Prosser F., Roma-Marzio F., Rosati L., Santangelo A., Scoppola A., Selvaggi A., Selvi F., Soldano A., Stinca A., Wagensommer R.P., Wilhalm T., Conti F., 2024 - A second update to the checklist of the vascular flora native to Italy. Plant Biosystems, 158(2):219-296.

Bartolucci F, Domina G, Angiolini C, Argenti C, Bacchetta G, Barberis D, Bertotto G, Bonari G, Calvia G, Candini F, Coltri F, Conti F, Del Guacchio E, Di Pietro R, Festi F, Fois M, Forte L, Galasso G, Gallo Splendore M, Koopman J, Lonati M, Mascia F, Minutillo F, Nascimbene J, Nota G, Pappagallo G, Pellegrino G, Podda L, Roffarè G, Ruggero A, Selvi F, Silletti G, Soldano A, Terranova C, Tondi G, Vallariello R, Zanatta K, Lastrucci L 2025 - Notulae to the Italian native vascular flora: 20. Italian Botanist 20: 89-107. https://doi.org/10.3897/italianbotanist.20.179511

Blasi C., Gigli M.P., Stanisci A., 1992 - I cespuglieti altomontani del gruppo del M. Velino (Italia centrale). Annali di Botanica (Roma), 48 (1990), Suppl. 7: 243-261.

Ciaschetti G., Pirone G., 2019. Lathyro asphodeloidis-Klaseetum lycopifoliae, a new plant association in the alliance Cynosurion cristati Tüxen, 1947 in Central Apennines. Italian Botanist, 7: 35-50.

Conti F., 1993 - Note floristiche per l'Italia centro-meridionale. Archivio Botanico e Biogeografico Italiano, 68 (1-2) (1992): 26-34.

Conti F., Bartolucci F., Manzi A., Miglio M., Tinti D., 2008 - Aggiunte alla Flora d'Abruzzo: III contributo. Annali del Museo civico di Rovereto., Sez.: Arch., St., Sc. Nat., 23(2007): 127-140.

Conti F., Cangelmi G., Da Valle J., De Santis E., Giacanelli V., Gubellini L., Hofmann N., Masin R.R., Miglio M., Palermo D., Santucci B., Bartolucci F., 2023 - Additions to the vascular flora of Italy. — Flora Mediterranea, 33: 177-191.

Cresti L., Schönswetter P., Peruzzi L., Barfuss M.H.J., Frajman B., 2019 - Pleistocene survival in three Mediterranean refugia: origin and diversification of the Italian endemic Euphorbia gasparrinii from the E. verrucosa alliance (Euphorbiaceae). Botanical Journal of the Linnean Society, 189(3): 262–280, https://doi.org/10.1093/botlinnean/boy082

Frizzi G., Tammaro F., Guarrera P., 1996 - Studio floristico delle Gole di Celano (Abruzzo - Italia) e principali tipologie vegetazionali. Micologia e Vegetazione Mediterranea, 11 (1): 33-52.

Galasso G., Conti F., Peruzzi L., Alessandrini A., Ardenghi N.M.G., Bacchetta G., Banfi E., Barberis G., Bernardo L., Bouvet D., Bovio M., Castello M., Cecchi L., Del Guacchio E., Domina G., Fascetti S., Gallo L., Guarino R., Gubellini L., Guiggi A., Hofmann N., Iberite M., Jiménez-Mejías P., Longo D., Marchetti D., Martini F., Masin R.R., Medagli P., Musarella C.M., Peccenini S., Podda L., Prosser F., Roma-Marzio F., Rosati L., Santangelo A., Scoppola A., Selvaggi A., Selvi F., Soldano A., Stinca A., Wagensommer R.P., Wilhalm T., Bartolucci F. (2024): A second update to the checklist of the vascular flora alien to Italy, Plant Biosystems, 158(2): 297-340, DOI:10.1080/11263504.2024.2320129

Groves E., 1880 - Flora del Sirente. Nuovo Giornale Botanico Italiano, 12: 51-68.

Guarrera P.M., Tammaro F., 1996 - La Flora del M. Sirente e zone limitrofe (Appennino Abruzzese). Annali di Botanica (Roma), 52 (1994), Suppl. 11 (2): 267-381.

Lastoria M., 2000 - Flora d'Abruzzo, 2. Deltagrafica, Teramo. 1-416.

Lucchese F., Lattanzi E., 1993 - Nuovo contributo alla Flora del Massiccio del Monte Velino (Appennino Abruzzese). Annali di Botanica (Roma), 49 (1991): 137-199.

Martelli U., 1904 - Una passeggiata sul Monte Velino e Montagne della Duchessa. Bullettino della Società Botanica italiana, 11: 110-115.

Montelucci G., 1958 - Appunti sulla vegetazione del Monte Velino (Appennino Abruzzese). Nuovo Giornale Botanico Italiano, n.s., 65 (1-2): 237-334.

Petriccione B., 1993 - Flora e Vegetazione del Massiccio del Monte Velino (Appennino Centrale), comprendente il territorio della Riserva Naturale Orientata «Monte Velino» e della foresta demaniale «Montagna della Duchessa» (con carta della vegetazione in scala 1:10.000). Minist. Agric. e Foreste, C.F.S., Collana Verde, 92. Tipo-Lito La Grotteria. Roma. 267 pp.

Petriccione B., 1994 - Flora and Vegetation mapping of Velino Massif (Abruzzo-Italy): a data source for a scientific management of a natural reserve. Fitosociologia, 26: 189-199.

Petriccione B., 2005 - Short-term changes in key plant communities of Central Apennines (Italy). Acta Botanica Gallica, 152 (4): 545-561.

Pezzetta A. 2016 – Le Orchidaceae della Provincia dell'Aquila, 85–104. Annales · Ser. hist. nat., · 26 ·(1): 85-104.

Pirone G., 1995 - Alberi, Arbusti e Liane d'Abruzzo. Cogecstre Edizioni, Penne. 543 pp.

Tammaro F., Veri L., Frizzi G., 1974 - Indagine botanica su alcuni pascoli montani abruzzesi. Rivista Abruzzese, 27 (4): 32 pp.

Tenore M., 1830 - Succinta relazione del viaggio fatto in Abruzzo ed in alcune parti dello Stato Pontificio dal Cavalier Tenore nell'Està del 1829. Stamperia della Società Filomatica: [1]-90 [91].

Tenore M., 1831 - Sylloge Plantarum Vascularium Florae Neapolitanae. Neapoli ex Typographia Fibreni.

Tiburtini M., Astuti G., Bartolucci F., Casazza G., Varaldo L., De Luca D., Bottigliero M.V., Bacchetta G., Porceddu M., Domina G. et al. 2022 - Integrative Taxonomy of Armeria arenaria (Plumbaginaceae), with a Special Focus on the Putative Subspecies Endemic to the Apennines. Biology 2022, 11, 1060. https://doi.org/10.3390/ biology11071060

Veri L., Tammaro F., 1980 - Aspetti vegetazionali del Monte Sirente (Appennino Abruzzese). C.N.R., Coll. Progr. Final. "Promozione della Qualità dell'ambiente", AQ/1/83. Roma. 22 pp. + map.
