# Supplementary material for: The Vascular Flora of Sirente-Velino Regional Park (Abruzzo, Central Italy)
Source: Biology (Basel). 2026 Jul 7;15(13):1093. doi: 10.3390/biology15131093 (PMC13359650; doi:10.3390/biology15131093)
Supplement: Supplementary file 1 [file biology-15-01093-s001.zip › Supp. fileS1.pdf]

SUPPLEMENTARY FILE S1 - Floristic Inventory

The species and subspecies may be preceded by the following symbols:

E: Endemic to Italy (including Malta and Corsica) (Peruzzi et al. 2014; Bartolucci et al. 2024a)
C: Cryptogenic (taxon doubtfully native) (Bartolucci et al. 2024a): "C"
Alien taxa (Galasso et al. 2024):
A: Archaeophyte
N: Neophyte
AR: Regional alien
AL: Local alien
#: Taxa occurring in the Monte Velino Nature Reserve
*: New taxa (species and subspecies) for the PRSV's flora

The localities cited in bibliography confirmed by us (through the collection of samples and personal observations) are indicated in the floristic list with the symbol "!".
Taxa doubtfully occurring (D) and no longer recorded (presence of a reliable historical record before 1965, NC) are indicated in the floristic list only in italics.
Alien taxa are followed by the status (CAS, casual; NAT, naturalised; INV, invasive).
The floristic list also includes conservation information relating to the inclusion of taxa in the Habitats Directive 92/43/EEC or in the Red List of Italian Flora (Rossi et al. 2013, 2020; Orsenigo et al. 2018). For the common and less common species within the Park's territory, we have added a note regarding the altitudinal zone in which the plant is distributed [hill and submontane belt (from 400 up to 1,000/1,100 m); montane belt (from 1000/1100 up to 1800/1900 m); subalpine belt (from 1800/1900 up to 2100/2200 m); alpine belt (above 2100/2200 m up to the peaks)]

FLORISTIC INVENTORY

Ferns and fern-allies

ASPLENIACEAE

\# ***Asplenium adiantum-nigrum*** L. subsp. ***adiantum-nigrum***
Distribution:—hill, submontane and montane belt.

\# ***Asplenium ceterach*** L. subsp. ***bivalens*** (D.E.Mey.) Greuter & Burdet (*Ceterach officinarum* Willd. subsp. *bivalens* D.E.Mey.)
Distribution:—common in the hill, submontane and montane belt.

Asplenium ceterach L. subsp. ***ceterach*** (*Ceterach officinarum* Willd. subsp. *officinarum*)
Distribution:—Mt. Sirente (Veri & Tammaro 1980).

\# ***Asplenium fissum*** Kit. ex Willd.

Asplenium fontanum (L.) Bernh. subsp. *fontanum* —D

Distribution:—Mt. Sirente (Groves 1880; Fiori 1943). Not observed recently, presence uncertain, probably to be excluded.

Asplenium lepidum C.Presl subsp. ***lepidum***

Distribution:—hill and submontane belt.

Asplenium onopteris L.

Distribution:—hill and submontane belt.

\# ***Asplenium ruta-muraria*** L. subsp. ***ruta-muraria***

Distribution:—common from hill, submontane to alpine belt.

\# ***Asplenium trichomanes*** L. subsp. ***quadrivalens*** D.E.Mey.

Distribution:—from hill, submontane to subalpine belt.

\# ***Asplenium viride*** Huds.

Distribution:—from montane to alpine belt.

ATHYRIACEAE

\# ***Athyrium filix-femina*** (L.) Roth

Distribution:—montane belt.

CYSTOPTERIDACEAE

Cystopteris alpina (Lam.) Desv.

Distribution:—subalpine and alpine belt.

Cystopteris dickieana R.Sim

Distribution:—Velino (Marchetti 2004).

\# ***Cystopteris fragilis*** (L.) Bernh.

Distribution:—montane to alpine belt.

\# ***Gymnocarpium robertianum*** (Hoffm.) Newman

Distribution:—V.ne di Teve! (Lucchese & Lattanzi 1993; APP 68440).

DENNSTAEDTIACEAE

\# ***Pteridium aquilinum*** (L.) Kuhn subsp. ***aquilinum***

Distribution:—montane belt.

DRYOPTERIDACEAE

\# ***Dryopteris filix-mas*** (L.) Schott

Distribution:—montane belt.

Dryopteris pallida (Bory) Maire & Petitm. subsp. ***pallida***

Distribution:—Cardore (Ocre)!, Pié di Sevice! (Bartolucci 2010; APP 34909).

\# ***Dryopteris villarii*** (Bellardi) Woyn. ex Schinz & Thell.

subalpine belt.

\# ***Polystichum aculeatum*** (L.) Roth

Distribution:—hill to alpine belt.

\# ***Polystichum lonchitis*** (L.) Roth

Distribution:—montane to alpine belt.

Polystichum setiferum (Forssk.) T.Moore ex Woyn.

Distribution:—montane belt.

EQUISETACEAE

Equisetum arvense L.

Distribution:—hill, submontane and montane belt.

Equisetum fluviatile L.

Distribution:—Torrente La Foce in Val d'Arano (Pirone 1997), Val d'Arano! (Ciaschetti et al. 2005; APP 28598, 28611, 28620, 28621, 37670), Conche di Ovindoli! (APP 66488, 66788).

Equisetum hyemale L.—NC

Distribution:—Rocca di Cambio (Montelucci 1971).

Equisetum palustre L.

Distribution:—hill, submontane and montane belt.

Equisetum ramosissimum Desf.

Distribution:—hill and submontane belt.

Equisetum telmateia Ehrh.

Distribution:—hill, submontane and montane belt.

OPHIOGLOSSACEAE

\# ***Botrychium lunaria*** (L.) Sw.

Distribution:—montane belt.

Ophioglossum vulgatum L.

Distribution:—Altopiano delle Rocche! (Conti et al. 2006; APP 11287, 67845), Campo Felice! (De Santis & Soldati 2011; APP 38350).

POLYPODIACEAE

Polypodium cambricum L.

Distribution:—Aielli and Gole di Celano (Guarrera & Tammaro 1996).

Polypodium interjectum Shivas

Distribution:—from laghetto Fonte dell'Acqua to Val Lupara (Guarrera & Tammaro 1996).

\# ***Polypodium vulgare*** L.

Distribution:—common from hill and submontane to montane belt.

PTERIDACEAE

Adiantum capillus-veneris L.

Distribution:—Gole di Celano (Guarrera & Tammaro 1996).

WOODSIACEAE

Woodsia alpina (Bolton) Gray—NC

Distribution:—Velino (Pichi Sermolli 1955 from a specimen in herb. Rolli).

Gymnosperms

CUPRESSACEAE

N ****Cupressus sempervirens*** L.—CAS

Distribution:—near the cemetery of Secinaro! (APP 74185).

N *__Hesperocyparis glabra__* (Sudw.) Bartel—CAS
Distribution:—near the cemetery of Secinaro! (APP 74193, 74194), cemetery of Molina Aterno! (pers. obs.).

__Juniperus communis__ L.
Note:—there are two varieties in the Park: var. *communis* in the mountain range and var. *saxatilis* Pall. in the subalpine range.

__Juniperus deltoides__ R.P.Adams
Distribution:—hill, submontane to montane belt.

__Juniperus oxycedrus__ L.
Distribution:—Belvedere Peligno above Vittorito! (Bartolucci et al. 2017).

N *__Platycladus orientalis__* (L.) Franco—NAT
Distribution:—Fagnano Alto near eremo of S. Rocco! (APP 68337).

EPHEDRACEAE

__Ephedra nebrodensis__ Tineo ex Guss. subsp. *__nebrodensis__*
Distribution:—Gole di S. Venanzio! (Anzalone 1961; Pirone 1995), Gole di Celano! (Conti & Manzi 1996).

PINACEAE

AL *__Abies alba__* Mill.—CAS
Distribution:—Pineta di S. Leucio! (pers. obs.), S. Felice d'Ocre in loc. Cardore! (APP 72768).

N *__Abies pinsapo__* Boiss. subsp. *__pinsapo__*—CAS
Distribution:—Colle (Fontecchio)! (APP 74223).

N, # *__Cedrus atlantica__* (Endl.) G.Manetti ex Carrière—CAS
Distribution:—near the cemetery of Secinaro!, S. Martino d'Ocre! (APP 72770, 74195), Pié Cafornia (pers. obs.).

N *__Cedrus deodara__* (Roxb. ex D.Don) G.Don—CAS
Distribution:—near the cemetery of Secinaro!, Ovindoli! (APP 74196, 74212).

AR, # *__Larix decidua__* Mill.—CAS
Distribution:—montane belt.

AR, # *__Picea abies__* (L.) H.Karst.—CAS
Distribution:—montane belt.

*__*Pinus halepensis__* Mill. subsp. *__halepensis__*
Distribution:—Gole di S.Venanzio!, between Secinaro and Goriano Valli! (pers. obs.).

Colt, # *__Pinus mugo__* Turra subsp. *__mugo__*
Distribution:—introduced on an experimental basis in V. Briganti (Petriccione 1993).

AL, # *__Pinus nigra__* J.F.Arnold subsp. *__nigra__*—NAT
Distribution:—hill, submontane and montane belt.

N, # *__*Pinus sylvestris__* L.—CAS
Distribution:—Pineta di S. Leucio!, Pié Cafornia! (pers. obs.).

TAXACEAE

Taxus baccata L.
Distribution:—Gole di Celano!, Valle Majelama! (Pirone & Conti 1990; Lucchese & Lattanzi 1993; Frizzi et al. 1996; Guarrera & Tammaro 1996).

Angiosperms

ALISMATACEAE

Alisma lanceolatum With.
Distribution:—Piana di Ovindoli!, Prati del Lago! (Ocre) (APP 66506, 68084).

Alisma plantago-aquatica L.
Distribution:—F. Aterno (Groves 1880), Rocca di Cambio (Buchwald 1996), Laghetto Fonte dell'Acqua (Guarrera & Tammaro 1995), San Benedetto in Perillis! (APP 6229), F. Aterno below Fossa! (APP 26765).

AMARANTHACEAE

N ***Amaranthus albus*** L.—NAT
Distribution:—Serra di Celano (Guarrera & Tammaro 1996), near Campana! (APP 74234).

Amaranthus graecizans L. subsp. ***silvestris*** (Vill.) Brenan
Distribution:—Massa d'Albe (Montelucci 1958)

N ****Amaranthus hybridus*** L. subsp. ***cruentus*** (L.) Thell.—CAS
Distribution:—near Secinaro!, Frascara!, near Colle (Fontecchio)! (APP 74198, 74236, pers. obs.).

N ***Amaranthus hybridus*** L. subsp. ***hybridus***—NAT
Distribution:—Gole di Celano (Frizzi et al. 1996; Guarrera & Tammaro 1996), F. Aterno close to Tione!, Molina Aterno!, Goriano Sicoli in loc. Macchione!, Fossa di Monticchio!, Fonteavignone! (APP 28203, 28235, 28482, 29956, 32345, 74204).

N ***Amaranthus powellii*** S.Watson—NAT
Distribution:—F. Aterno below Fossa!, Tione degli Abruzzi!, Gagliano Aterno in loc. Recolle! (Iamonico et al. 2011; APP 26767, 28204, 28345).

N ***Amaranthus retroflexus*** L.—INV
Distribution:—common in the hill, submontane and lower montane belt.

A ***Atriplex hortensis*** L. subsp. ***hortensis***—NAT
Distribution:—in the neighboring territory of the Fossa di Monticchio! (APP 32342, 32346, 32347).

****Atriplex patula*** L. subsp. ***patula***
Distribution:—Goriano Valli! (APP 74237), Campo Felice!, Secinaro! (pers.

N ***Bassia scoparia*** (L.) Voss—CAS
Distribution:—road margins near Villa S. Angelo (Guarrera & Tammaro 1996).

Blitum bonus-henricus (L.) Rchb. (*Chenopodium bonus-henricus* L.)
Distribution:—common in the montane belt.

Chenopodiastrum hybridum (L.) S.Fuentes, Uotila & Borsch (*Chenopodium hybridum* L.)
Distribution:—submontane and montane belt.

Chenopodium album L. subsp. **album**
Distribution:—hill and submontane belt.

Chenopodium vulvaria L.
Distribution:—hill and submontane belt.

Lipandra polysperma (L.) S.Fuentes, Uotila & Borsch (*Chenopodium polyspermum* L.)
Distribution:—hill and submontane belt.

***Salsola tragus** L.
Distribution:—near Forme! (pers. obs.). Species new for the flora of Abruzzo.

AMARYLLIDACEAE

Allium angulosum L.
Distribution:—Prato della Madonna! (Conti et al. 2019a; APP 59534, 59536, 59542).

***Allium atroviolaceum** Boiss.
Distribution:—Massa d'Albe! (pers. obs.).
Note:—Currently, the species is being studied by J.M. Tison and E. Vela.

Allium cirrhosum Vand. (*A. coloratum* Spreng).
Distribution:—near laghetto Fonte dell'Acqua, beech forests and clearings (1200-1300 m) (Guarrera & Tammaro 1996).

E, # **Allium ducissae** Bartolucci, Iocchi & F.Conti—NT
Distribution:—Velino (Petriccione 1993 as *Allium lineare*), Mt. Rozza!, Cimata di Pezza! It is endemic to Velino massif and is also known for Mt. Orsello and Duchessa (Murolungo and Morrone)! (Bartolucci et al. 2022).

Allium flavum L. subsp. **flavum**
Distribution:—submontane and montane belt.

Allium horvatii Lovrić (*A. saxatile* M.Bieb. subsp. *tergestinum* (Gand.) Bedalov & Lovrić, nom. inval.; *A. tergestinum* Gand., nom. inval.)
Distribution:—submontane and montane belt.

Allium lusitanicum Lam.
Distribution:—montane and subalpine belt.

Allium moschatum L.
Distribution:—submontane and montane belt.

Allium nigrum L.
Distribution:—hill and submontane belt.

Allium oleraceum L. subsp. **oleraceum**
Distribution:—hill, submontane and montane belt.

Allium pallens L.—NC
Distribution:—Velino (Tenore 1830).

***Allium pendulinum** Ten.—LC
Distribution:—Eremo della Madonna di Pietrabona!, Cerreta di Goriano Sicoli! (APP 72868, 73002).

Allium permixtum Guss.—VU
Distribution:—Settacque! (Bartolucci et al. 2014; De Santis & Soldati 2019; pers. obs.), Conche di Ovindoli! (Conti et al. 2023a; APP 66503).

Allium polyanthum Schult. & Schult.f.—DD
Distribution:—indicated in the neighboring territory of Lucoli (De Santis & Soldati 2019) and likely also present in the Park.

E, # ***Allium samniticum*** Brullo, Pavone & Salmeri—LC
Distribution:—Valle Majelama, V.ne di Teve (Lucchese & Lattanzi 1993 as *A. cupani*).

Allium sphaerocephalon L. subsp. ***arvense*** (Guss.) Arcang. (*A. arvense* Guss.)
Distribution:—Piani di Pezza (Marcucci & Tornadore 1997).

Allium sphaerocephalon L. subsp. ***sphaerocephalon***
Distribution:—common in the hill, submontane and montne belt.

Allium tenuiflorum Ten.
Distribution:—common from the hill to subalpine belt.

Allium ursinum L.
Distribution:—montane belt.

Allium vineale L.
Distribution:—montane belt.

Galanthus nivalis L.—LC
Distribution:—montane belt.

Narcissus poëticus L.
Distribution:—montane belt.

****Sternbergia colchiciflora*** Waldst. & Kit.
Distribution:—sothern slopes of Mt. Cefalone! and Mt. Orsello! (pers. obs.).

****Sternbergia lutea*** (L.) Ker Gawl. ex Spreng.
Distribution:—Fossa Tarone (between S. Benedetto in Perillis and Popoli)! (APP 46537), Castelvecchio Subequo! (pers. obs.).

ANACARDIACEAE

Cotinus coggygria Scop.
Distribution:—Gole di San Venanzio!, Castelvecchio Subequo (D'Errico 1936; Montelucci 1971; Tammaro & Visca 1987; Pirone 1995; Corbetta et al. 2004).

Pistacia terebinthus L. subsp. ***terebinthus***
Distribution:—hill and submontane belt.

AR ****Rhus coriaria*** L.—NAT
Distribution:—at the start of the trail to the mountain church, above Celano towards Ovindoli! (APP 68392), Forme! (pers. obs.).

APIACEAE

Aegopodium podagraria L.
Distribution:—montane belt.

Ammoides pusilla (Brot.) Breistr.
Distribution:—hill and submontane belt.

Anethum foeniculum L. (*Foeniculum vulgare* Mill.)
Distribution:—hill and submontane belt.

Anethum piperitum Ucria (*Foeniculum vulgare* Mill. subsp. *piperitum* (Ucria) Bég.)
Distribution:—hill and submontane belt.

Anethum ridolfia Spalik & Reduron (*Ridolfia segetum* (L.) Moris)—NC
Distribution:—cliffs of Rio below Castevecchio Subequo (Groves 1880).

Angelica sylvestris L. subsp. ***sylvestris***
Distribution:—montane belt.

A ****Anthriscus cerefolium*** (L.) Hoffm.—CAS
Distribution:—Rosciolo! (pers. obs.).

Anthriscus nemorosa (M.Bieb.) Spreng.
Distribution:—montane belt.

Anthriscus sylvestris (L.) Hoffm. subsp. ***sylvestris***
Distribution:—montane belt.

Astrantia major L. subsp. ***involucrata*** (W.D.J.Koch) Ces.
Distribution:—montane belt.

E *Astrantia pauciflora* Bertol. subsp. *tenorei* (Mariotti) Bechi & Garbari—D, DD
Distribution:—"regione della macchia" (Groves 1880 as *A. pauciflora*), Ovindoli (Guarrera & Tammaro 1996 from a specimen collected by Chiarugi and kept at FI). Both reports are implausible because they place it at lower altitudes than those at which it is found. Its habitat is in fact primary grasslands. Mariotti (1989) does not mention any specimens from Velino-Sirente preserved in FI, despite having carried out extensive herbarium research.

Berula erecta (Huds.) Coville
Distribution:—laghetto Fonte dell'Acqua (Guarrera & Tammaro 1996), F. Aterno near Molina! (APP 27214), laghetto of Campo di Rovere! (pers. obs.).

****Bifora radians*** M.Bieb.
Distribution:—Mt. Offermo!, near Campana! (APP).

****Bifora testiculata*** (L.) Spreng.
Distribution:—Goriano Sicoli! (APP), Collarmele! (pers. obs.).

Bunium bulbocastanum L.
Distribution:—from the hill, submontane to subalpine belt.

E ***Bunium petraeum*** Ten.—LC
Distribution:—Mt. Puzzillo, Mt. Costone (Lucchese & Lattanzi 1993). The indication to Piani di Pezza (Ciaschetti et al. 2006) is definitely incorrect and should refer to *B. bulbocastanum* .

Bupleurum baldense Turra
Distribution:—hill, submontane to montane belt.

Bupleurum falcatum L. subsp. ***cernuum*** (Nyman) Arcang.
Distribution:—from montane to subalpine belt.

Bupleurum gerardi All.
Distribution:—Velino (Tammaro et al. 1988), Ovindoli! (APP).

Bupleurum praealtum L.
Distribution:—montane belt.

Bupleurum subovatum Link ex Spreng.
Distribution:—montane belt.

***Carum carvi** L.*

Distribution:—Conche di Ovindoli! (APP 66521, 66523).

E, # ***Carum carvifolium*** (DC.) Arcang. (*C. flexuosum* (Ten.) Nyman, nom. illeg.; *C. heldreichii* auct. Fl. Ital.)

Distribution:—frequent on rocky slopes and high-altitude scree slopes (Tenore 1830, 1831; Groves 1880; Martelli 1904; Montelucci 1958; Veri & Tammaro 1980; Blasi et al. 1992; Lucchese & Lattanzi 1993; Petriccione 1993; Guarrera & Tammaro 1996; Petriccione 2005; Ciaschetti et al. 2006; APP 67190, 72829).

Caucalis platycarpos L.

Distribution:—Goriano Sicoli! (APP 73509), Massa d'Albe! (pers. obs.).

Chaerophyllum aureum L.

Distribution:—hill, submontane to montane belt.

Chaerophyllum hirsutum L.

Distribution:—montane belt.

E ***Chaerophyllum magellense*** Ten.—LC

Distribution:—Mt. Sirente, between Celano and Ovindoli (Groves 1880; Guarrera & Tammaro 1996), Valle Cerchiata (Lucchese & Lattanzi 1993), Costa della Tavola! (APP 67621).

Chaerophyllum nodosum (L.) Crantz (*Myrrhoides nodosa* (L.) Cannon)

Distribution:—hill, submontane to montane belt.

Chaerophyllum temulum L.

Distribution:—common in the hill, submontane to montane belt.

Conium maculatum L. subsp. ***maculatum***

Distribution:—hill, submontane to montane belt.

A ***Coriandrum sativum*** L.—CAS

Distribution:—Velino (Viegi et al. 1990 from a specimen collected by Levier).

E, # ***Coristospermum cuneifolium*** (Guss.) Bertol. (*Ligusticum cuneifolium* Guss.; *L. lucidum* Mill. subsp. *cuneifolium* (Guss.) Tammaro; *L. lucidum* auct. Fl. Ital. p.p.)—LC

Distribution:—Velino!, Mt. Sirente! (Tenore 1830, 1831; Groves 1880; Abbate 1903; Adamovic 1933; Steinberg 1953; Montelucci 1958; Avena & Blasi 1975; Tammaro 1990; Petriccione 1993; Guarrera & Tammaro 1996; pers. obs.), Mt. Cefalone! (APP 67420, 67437, 67438).

E, # ***Daucus broteroi*** Ten.—LC

Distribution:—cliffs of Rio (below Castelvecchio Subequo) (Groves 1880), V.ne di Teve (Lucchese & Lattanzi 1993).

Daucus carota L. subsp. ***carota***

Distribution:—common from the hill, submontane to montane belt.

Daucus carota L. subsp. ***maximus*** (Desf.) Ball

Distribution:—Rovere (Guarrera & Tammaro 1996).

\# ***Eryngium amethystinum*** L.

Distribution:—common in the hill, submontane to montane belt.

Eryngium campestre L.

Distribution:—common in the hill, submontane to montane belt.

Falcaria vulgaris Bernh.

Distribution:—common in the hill, submontane belt.

Ferula glauca L.

Distribution:—The report of *F. communis* subsp. *communis* for Rocca di Cambio is implausible, as well those for Gole di Celano! (Anzalone et al. 1992; Guarrera & Tammaro 1996; Frizzi et al. 1996) and Gole di S. Venanzio! (Lastoria 2000). These reports should be referred to *F. glauca*, which has already been reported for Gole di S. Venanzio (Pirone 1987) and Gole di Celano! (Conti 1998; APP 40618, 66999). Also observed in loc. Forme! (pers. obs.).

Geocaryum cynapioides (Guss.) Engstrand

Distribution:—montane belt.

Grafia golaka (Hacq.) Rchb.

Distribution:—nontane belt.

Helosciadium nodiflorum (L.) W.D.J.Koch subsp. ***nodiflorum*** (*Apium nodiflorum* (L.) Lag.)

Distribution:—hill, submontane to montane belt.

Heracleum orsinii Guss.

Distribution:—montane to subalpine belt.

Heracleum sibiricum L. subsp. ***sibiricum***

Distribution:—Mt. Ventrino (Guarrera & Tammaro 1996).

Heracleum sibiricum L. subsp. ***ternatum*** (Velen.) Briq.

Distribution:—hill, submontane to montane belt.

Katapsuxis silaifolia (Jacq.) Reduron, Charpin & Pimenov (*Cnidium silaifolium* (Jacq.) Simonk.; *Laserpitium silaifolium* Jacq.; *Selinum silaifolium* (Jacq.) Beck)

Distribution:—montane belt.

Laserpitium gallicum L. subsp. ***gallicum***

Distribution:—Velino (Martelli 1904; Tammaro et al. 1988; Petriccione 1993), Valle di Sevice!, Valle Majelama (Lucchese & Lattanzi 1993; APP 59803), Vallone dell'Orso! (APP 34319, 34320), between Campo Felice and Forca Miccia! (pers. obs.).

Laserpitium latifolium L.

Distribution:—montane belt.

Lophosciadium galbaniferum (Mill.) Lyskov & Akalın (*Ferulago galbanifera* (Mill.) W.D.J.Koch; *F. campestris* (Besser) Grecescu)

Distribution:—Val d'Arano! (APP 73538), near S. Panfilo!, between Campana and Fontecchio! (pers. obs.). The report for Campo Felice (Lucchese & Lattanzi 1993) most likely refers to *Prangos ferulacea*, which is quite common in this

Meum athamanticum Jacq.

Distribution:—Valle Lupara (Guarrera & Tammaro 1996).

Oenanthe fistulosa L.—NT

Distribution:—Val d'Arano! (Conti 1998; Conti et al. 1999), Piana di Ovindoli!, Colle Ciaccio! (APP 9488, 9489, 72926).

Oenanthe pimpinelloides L.

Distribution:—common in the hill, submontane to montane belt.

Oenanthe silaifolia M.Bieb.

Distribution:—Val d'Arano! (Conti 1998; APP 28608, 28609), Conche di Ovindoli!, Campo di Rovere!, Prati del Lago! (APP 25285, 28673, 66507, 66508, 67855, 67858, 68077, 68078).

Opopanax chironium (L.) W.D.J.Koch

Distribution:—Massa d'Albe (Martelli 1904; Steinberg 1953; Montelucci 1958), S. Panfilo d'Ocre!, between S. Martino and Terranera! (APP 67702, 67703, 67704).

Oreoselinum nigrum Delarbre

Distribution:—montane belt.

Orlaya daucorlaya Murb.—LC

Distribution:—hill and submontane belt.

Orlaya grandiflora (L.) Hoffm.

Distribution:—hill, submontane to montane belt.

Orlaya platycarpos W.D.J.Koch

Distribution:—hill, submontane to montane belt.

Pastinaca sativa L. subsp. ***urens*** (Req. ex Godr.) Čelak.

Distribution:—hill, submontane to montane belt.

A ***Petroselinum crispum*** (Mill.) Fuss—CAS

Distribution:—hill and submontane belt.

A ***Pimpinella anisum*** L.—CAS

Distribution:—Velino (Parlatore 1848-96), Massa d'Albe, Celano (Tammaro et al. 1988 from a specimen collected by Levier and indicated as cultivated).

Pimpinella major (L.) Huds.

Distribution:—hill, submontane to montane belt.

Pimpinella saxifraga L. subsp. ***saxifraga***

Distribution:—hill, submontane to montane belt.

Pimpinella tragium Vill.

Distribution:—hill, submontane to montane belt.

Prangos ferulacea (L.) Lindl. (*Cachrys ferulacea* (L.) Calest.)

Distribution:—montane belt.

Pteroselinum austriacum (Jacq.) Rchb. (*Peucedanum austriacum* (Jacq.) W.D.J.Koch)

Distribution:—montane belt.

Ptychotis saxifraga (L.) Loret & Barrandon

Distribution:—near Secinaro (Tammaro et al. 1988; Guarrera & Tammaro 1996).

Sanicula europaea L.

Distribution:—common in the montane belt.

Scandix australis L. subsp. ***australis***

Distribution:—hill and submontane belt.

****Scandix macrorhyncha*** C.A.Mey.

Distribution:—between Vado della Forcella and Prati del Sirente! (APP 72849).

Scandix pecten-veneris L. subsp. ***pecten-veneris***

Distribution:—hill and submontane belt.

Seseli libanotis (L.) W.D.J.Koch

Distribution:—montane and subalpine belt.

Seseli montanum L. subsp. ***montanum***

Distribution:—hill, submontane to montane belt.

Seseli pallasii Besser

Distribution:—the report as *S. annuum* for Terranera (Conti et al. 2025a) should be referred to this taxon (Bartolucci et al. 2025).

Seseli tommasinii Rchb.f.

Distribution:—hill and submontane belt.

Seseli tortuosum L. subsp. ***tortuosum***

Distribution:—hill and submontane belt.

Siler montanum Crantz subsp. *garganicum* (Ten.) Iamonico, Bartolucci & F.Conti (*Laserpitium garganicum* (Ten.) Bertol.; *L. siler* L. subsp. *garganicum* (Ten.) Arcang.) —NC

Distribution:—Velino (Tenore 1831). Most of the subsequent reports are due to confusion with *Siler montanum* subsp. *stabianum.*

E, # ***Siler montanum*** Crantz subsp. ***stabianum*** (Lacaita) F.Conti & Bartolucci (*Laserpitium garganicum* (Ten.) Bertol. var. *stabianum* (Lacaita) Pignatti; *L. siculum* Spreng. var. *stabianum* Lacaita)

Note:—taxonomic treatment and distribution in Conti et al. (2021).

Sison amomum L.

Distribution:—hill and submontane belt.

Thapsia asclepium L.

Distribution:—hill and submontane belt.

Tordylium apulum L.

Distribution:—common in the hill and submontane belt.

Tordylium maximum L.

Distribution:—hill and submontane belt.

Torilis africana Spreng.

Distribution:—reported in the neighboring territory of Lucoli (De Santis & Soldati 2019) and likely also present in the Park.

Torilis arvensis (Huds.) Link subsp. ***arvensis***

Distribution:—hill and submontane belt.

Torilis japonica (Houtt.) DC.

Distribution:—hill and submontane belt.

Torilis leptophylla (L.) Rchb.f.

Distribution:—hill and submontane belt.

Trinia dalechampii (Ten.) Janch.

Distribution:—from montane to alpine belt.

Trinia glauca (L.) Dumort. subsp. ***glauca***

Distribution:—hill, submontane and montane belt.

****Turgenia latifolia*** (L.) Hoffm.

Distribution:—Prata d'Ansidonia!, M. Offermo!, San Benedetto in Perillis!, Eremo della Madonna di Pietrabona!, Goriano Sicoli! (APP 26698, 28097, 28101, 28103, 59828, 59831, 68180, 72884, 73510).

Xanthoselinum venetum (Spreng.) Soldano & Banfi (*Peucedanum venetum* (Spreng.) W.D.J.Koch)

Distribution:—hill and submontane belt.

APOCYNACEAE

Vinca major L. subsp. ***major***

Distribution:—hill and submontane belt.

\# ***Vinca minor*** L.

Distribution:—Velino (Avena & Blasi 1975).

\# ***Vincetoxicum hirundinaria*** Medik. subsp. ***hirundinaria***

Distribution:—common in the hill, submontane and montane belt.

AQUIFOLIACEAE

Ilex aquifolium L.

Distribution:—montane belt.

ARACEAE

\# ***Arum italicum*** Mill. subsp. ***italicum***

Distribution:—hill and submontane belt.

Arum maculatum L.

Distribution:—montane belt.

****Biarum tenuifolium*** (L.) Schott subsp. ***tenuifolium***

Distribution:—Carrito! (pers. obs.).

Lemna gibba L.

Distribution:—Gole di S. Venanzio! (Conti et al. 2008a).

Lemna minor L.

Distribution:—hill, submontane and montane belt.

N ***Lemna minuta*** Kunth—NAT

Distribution:—Fossa! (Iberite et al. 2008; APP 26772); Ovindoli close to Laghetto! (APP 26772), Sorgente la Solfa! (pers. obs.).

Spirodela polyrhiza (L.) Schleid.—NC

Distribution:—Mt. Sirente along F. Aterno (Groves 1880).

ARALIACEAE

N ***Hedera algeriensis*** Rantonnet ex C.Morren—CAS

Distribution:—cemetery of Secinaro! (APP 74187, 74188, 74189, 74190 74191, 74192; Conti et al. in press).

\# ***Hedera helix*** L. subsp. ***helix***

Distribution:—common in the hill, submontane and montane belt.

ARISTOLOCHIACEAE

\# ***Aristolochia lutea*** Desf.

Distribution:—common in the hill, submontane and montane belt.

****Asarum europaeum*** L.

Distribution:—beech forest of Sacco Sirente! (APP 27728).

ASPARAGACEAE

Anthericum liliago L.
Distribution:—montane belt.

Asparagus acutifolius L.
Distribution:—common in the hill and submontane belt.

AR **Asparagus officinalis** L. subsp. ***officinalis***—CAS
Distribution:—above Secinaro (Guarrera & Tammaro 1996), Lago Acquaviva near Molina Aterno! (APP 27211, 73037), between Castel di Ieri and Castelvecchio Subequo! (pers. obs.).

Asparagus tenuifolius Lam.
Distribution:—Velino (Petriccione 1993).

Bellevalia romana (L.) Sweet—LC
Distribution:—Alba Fucens (pers. obs.).

Convallaria majalis L.
Distribution:—montane belt.

N ***Hyacinthoides non-scripta*** (L.) Chouard ex Rothm.—CAS
Distribution:—Massa d'Albe! (pers. obs.). Species new for the flora of Abruzzo.

Loncomelos brevistylum (Wolfner) Dostál (*Ornithogalum brevistylum* Wolfner)
Distribution:—Gole di Celano! (APP), Collarmele! (pers. obs.).

Loncomelos narbonense (L.) Raf. (*Ornithogalum narbonense* L.)
Distribution:—Massa d'Albe (Lucchese & Lattanzi 1993).

Muscari botryoides (L.) Mill. subsp. *botryoides*—NC
Distribution:—Velino (Montelucci 1958).

Muscari comosum (L.) Mill.
Distribution:—common in the hill and submontane belt.

Muscari neglectum Guss. ex Ten. & Sangiovanni
Distribution:—common in the hill, submontane and montane belt.

Ornithogalum comosum L.
Distribution:—from the hill to alpine belt.

Ornithogalum divergens Boreau
Distribution:—hill, submontane and montane belt.

E ***Ornithogalum etruscum*** Parl. subsp. ***etruscum***—LC
Distribution:—M. Ocre in Vallone Canavine!, above Casentino! (APP 15334, 26818), Terranera!, Piani di Pezza! (pers. obs.).

E ***Ornithogalum exscapum*** Ten.
Distribution:—Goriano Sicoli! (APP 73476).

E ***Ornithogalum orthophyllum*** Ten.—DD
Distribution:—Velino above Fonte Canale, Coste del Cafornia, V.ne di Sevice (Lucchese & Lattanzi 1993; Petriccione 1993), Vaccarita (M. Ocre), Mt. Ventrino (Corbetta et al. 2004), Campo Felice in loc. F.te Camardosa! (APP 30169), close to Forca Caruso! (pers. obs.).

Ornithogalum refractum Willd.
Distribution:—M. Ocre near Fossa! (APP 68026).

Polygonatum multiflorum (L.) All.
Distribution:—hill, submontane and montane belt.

Polygonatum odoratum (Mill.) Druce
Distribution:—hill, submontane and montane belt.

***Polygonatum verticillatum** (L.) All.
Distribution:—from the Mt. Sirente chalet towards the summit!, Neviera!, V.ne di Teve! (APP 15615, 15616, 40590, 68464, 73399).

Prospero autumnale (L.) Speta
Distribution:—hill, submontane and montane belt.

Ruscus aculeatus L.—LC
Distribution:—hill and submontane belt.

Ruscus hypoglossum L.
Distribution:—reported in the neighboring territory of Lucoli (De Santis & Soldati 2019) and likely also present in the Park.

Scilla bifolia L. (*S. autumnalis* L.)
Distribution:—montane belt.

N ***Yucca filamentosa*** L.—CAS
Distribution:—cemetery di Molina Aterno! (APP 74160; Conti et al. in press).

N ***Yucca gloriosa** L.—CAS
Distribution:—surroundings of Fontecchio! (pers. obs.).

ASPHODELACEAE

Asphodeline liburnica (Scop.) Rchb.—LC
Distribution:—Gole di S. Venanzio! (Tammaro et al. 1980).

Asphodeline lutea (L.) Rchb.
Distribution:—hill, submontane and montane belt.

Asphodelus fistulosus L.
Distribution:—hill and submontane belt.

Asphodelus macrocarpus Parl. subsp. ***macrocarpus*** (*A. albus* auct. Fl. Ital.)
Distribution:—montane belt.

Asphodelus ramosus L. subsp. ***ramosus***
Distribution:—Gole di S. Venanzio! (Conti et al. 2008a).

ASTERACEAE

E, # ***Achillea barrelieri*** (Ten.) Sch.Bip. subsp. ***barrelieri***—LC
Note:—the reports of *A. barrelieri* (Ten.) Sch.Bip. subsp. *mucronulata* (Bertol.) Heimerl (Groves 1880 as *Anthemis mucronulata* var. *corymbulosa*; Petriccione 1993, 1994) should to be referred to this taxon.

E *Achillea barrelieri* (Ten.) Sch.Bip. subsp. *mucronulata* (Bertol.) Heimerl—D
Note:—Bazzichelli (1972) observed herbarium specimens (FI) with characteristics intermediate between this taxon and the previous one.

Achillea collina (Becker ex Wirtg.) Heimerl
Distribution:—hill, submontane and montane belt.

Achillea millefolium L. subsp. ***millefolium***
Distribution:—montane belt.

***Achillea nobilis** L.
Distribution:—between Rocca di Mezzo and Piani di Pezza! (APP 66689).

#	***Achillea setacea*** Waldst. & Kit.
Distribution:—montane belt.

E	***Achillea tenorei*** Grande—LC
Distribution:—Colle dell'Orso, Mt. Bicchero, Mt. Cafornia (Lucchese & Lattanzi 1993),

E, #	***Adenostyles australis*** (Ten.) Iamonico & Pignatti—LC
Distribution:—widespread in the park's megaforbs.

****Antennaria dioica*** (L.) Gaertn.
Distribution:—Campo Felice! (pers. obs.).

Anthemis arvensis L. subsp. ***arvensis***
Distribution:—montane belt.

Anthemis cotula L.
Distribution:—hill, submontane and montane belt.

#	***Anthemis cretica*** L. subsp. ***columnae*** (Ten.) Franzén
Distribution:—montane and subalpine belt.

Arctium lappa L.—D
Distribution:—Mt. Sirente (Veri & Tammaro 1980; Guarrera & Tammaro 1996), Velino (Petriccione 1993). Probably referring to *A. minus* .

#	***Arctium minus*** (Hill) Bernh.
Distribution:—hill, submontane and montane belt.

Arctium nemorosum Lej.
Distribution:—hill, submontane and montane belt.

Artemisia absinthium L.
Distribution:—hill, submontane and montane belt.

#	***Artemisia alba*** Turra
Distribution:—hill, submontane and montane belt.

Artemisia atrata Lam.—EN
Distribution:—Piani di Pezza! (Conti et al. 2006), Campo Felice! (De Santis & Soldati 2011).

Artemisia campestris L.
Distribution:—Gole di Celano as *A. campestris* subsp. *glutinosa* (J.Gay ex Besser) Batt. (Frizzi et al 1996; Guarrera & Tammaro 1996). Also the report of *A. campestris* subsp. *variabilis* for the same locality (Tammaro & Frizzi 1984), and not confirmed later by the same authors (Frizzi et al 1996; Guarrera & Tammaro 1996), probably refers to the subsp. *glutinosa* . We found *A. campestris* at Mt. Briccialone (APP 59719, 59720, 59721) but it seems to us belonging to *A. campestris* subsp. *campestris* . The species needs systematic revision.

N	***Artemisia verlotiorum*** Lamotte—INV
Distribution:—hill, submontane and montane belt.

Artemisia vulgaris L.
Distribution:—hill, submontane and montane belt.

#	***Aster alpinus*** L. subsp. ***alpinus***
Distribution:—subalpine belt.

#	***Bellidiastrum michelii*** Cass.
Distribution:—from montane to alpine belt.

Bellis perennis L.

Distribution:—common in he hill, submontane and montane belt.

Bellis sylvestris Cirillo

Distribution:—hill, submontane and montane belt.

N ****Bidens frondosa*** L.—NAT

Distribution:—F. Aterno near Tione!, laghetto di Goriano Valli!, Lago di Civita! (APP 28252, 34856, 74238, 74239).

****Bidens tripartita*** L. subsp. ***tripartita***

Distribution:—F. Aterno near Fossa!, Lago di Civita! (APP 26755, 26756,

\# ***Bombycilaena erecta*** (L.) Smoljan.

Distribution:—hill, submontane and montane belt.

Calendula arvensis (Vaill.) L.

Distribution:—common in the hill and submontane belt.

A ***Calendula officinalis*** L.—CAS

Distribution:—hill and submontane belt.

E ***Carduus affinis*** Guss. subsp. ***affinis***—LC

Distribution:—Velino (Martelli 1904; Montelucci 1958), Prati Canale! (Guarrera & Tammaro 1996; pers. obs.), Settacque (De Santis & Soldati 2019).

E, # ***Carduus chrysacanthus*** Ten.

Note: recently excluded from Croatia, the species must therefore be considered endemic to the Apennines (Šegota et al. 2025).

\# ***Carduus defloratus*** L. subsp. ***carlinifolius*** (Lam.) Ces.

Distribution:—from montane belt to alpine belt.

Carduus nutans L. subsp. ***nutans***

Distribution:—hill, submontane and montane belt.

E ***Carduus nutans*** L. subsp. ***perspinosus*** (Fiori) Arènes—LC

Distribution:—widespread in the pastures at the foot of the mountains.

Carduus pycnocephalus L. subsp. ***pycnocephalus***

Distribution:—common in the hill and submontane belt.

\# ***Carlina acanthifolia*** All. subsp. ***acanthifolia***

Distribution:—montane belt.

\# ***Carlina acaulis*** L. subsp. ***caulescens*** (Lam.) Schübl. & G.Martens

Distribution:—common in the montane belt.

\# ***Carlina corymbosa*** L.

Distribution:—hill and submontane belt.

Carlina vulgaris L. subsp. ***spinosa*** (Velen.) Vandas

Note:—previous reports of *C. vulgaris* should be referred to this taxon.

Carthamus lanatus L.

Distribution:—hill and submontane belt.

E ***Centaurea ambigua*** Guss. subsp. ***ambigua***—LC

Note:—very common but with an original combination of characteristics that differs from the typical subspecies. The authors have begun a morphological study of the species.

E, # ***Centaurea ambigua*** Guss. subsp. ***nigra*** (Fiori) Pignatti—LC

Distribution:—it is fairly widespread on rocky slopes at high altitudes.

Centaurea calcitrapa L.
Distribution:—hill and submontane belt.

E, # ***Centaurea ceratophylla*** Ten. subsp. ***ceratophylla***—LC
Distribution:—it is fairly widespread mainly on termphilous cliffs, sporadically reaching primary grasslands.

A, # ***Centaurea cyanus*** L.—NAT
Distribution:—hill, submontane and montane belt.

Centaurea deusta Ten. subsp. ***deusta***
Distribution:—hill and submontane belt.

\# ***Centaurea jacea*** L. subsp. ***gaudinii*** (Boiss. & Reut.) Gremli (*C. bracteata* Scop.)
Distribution:—hill, submontane and montane belt.

Centaurea jacea L. subsp. ***jacea***
Distribution:—montane belt.

E ***Centaurea nigrescens*** Willd. subsp. ***neapolitana*** (Boiss.) Dostál—DD
Distribution:—Mt. Sirente! (Groves 1889; Guarrera & Tammaro 1996), Campo Felice (De Santis & Soldati 2011), Mt. Ocre at the crossroad for Cavalletto! (APP 32339, 32340, 32341, 66808, 66809).

Centaurea rupestris L.
Distribution:—Massa d'Albe!, Mt. Cativiglia! (pers. obs.). Also reported for V.ne di Teve (Lucchese & Lattanzi 1993), where we found only *C. ceratophylla* subsp. *ceratophylla*.

Centaurea scabiosa L. subsp. ***scabiosa***
Distribution:—hill, submontane and montane belt.

\# ***Centaurea solstitialis*** L. subsp. ***solstitialis***
Distribution:—hill and submontane belt.

\# ***Centaurea triumfettii*** All.
Distribution:—from hill, submontane to alpine belt.

Chondrilla juncea L.
Distribution:—hill and submontane belt.

Cichorium intybus L.
Distribution:—hill and submontane belt.

Cirsium acaulon (L.) Scop. subsp. ***acaulon***
Distribution:—montane belt.

\# ***Cirsium arvense*** (L.) Scop.
Distribution:—hill, submontane and montane belt.

****Cirsium creticum*** (Lam.) d'Urv. subsp. ***triumfettii*** (Lacaita) K.Werner
Distribution:—Mt. Sirente along F.Aterno (Groves 1880 as *C. creticum*), near Molina Aterno! (APP 27185).

Cirsium vulgare (Savi) Ten.—LC
Distribution:—hill, submontane and montane belt.

Cota altissima (L.) J.Gay (*Anthemis altissima* L.)
Distribution:—hill and submontane belt.

****Cota segetalis*** (Ten.) Holub (*Anthemis segetalis* Ten.)
Distribution:—Forme! (APP 73365).

Cota tinctoria (L.) J.Gay subsp. **australis** (R.Fern.) Oberpr. & Greuters (*Anthemis tinctoria* L. subsp. *australis* R.Fern.)

Distribution:—hill, submontane and montane belt.

Cota triumfettii (L.) J.Gay (*Anthemis triumfettii* (L.) DC.)

Distribution:—Valle di Sevice (Lucchese & Lattanzi 1993).

Crepis aurea (L.) Cass. subsp. **glabrescens** (Caruel) Arcang.

Distribution:—subalpine and alpine belt.

Crepis biennis L.

Distribution:—hill, submontane and montane belt.

C ***Crepis bursifolia** L.

Distribution:—Molina Aterno!, Secinaro!, staz. Fagnano-Campana!, Gagliano Aterno!, Acciano!, Celano! (APP 73576, 74186, 74232, pers. obs.).

Crepis foetida L. subsp. **foetida**

Distribution:—hill and submontane belt.

Crepis lacera Ten. subsp. **lacera**

Distribution:—hill, submontane and montane belt.

Crepis neglecta L. subsp. **neglecta**

Distribution:—hill and submontane belt.

Crepis pulchra L. subsp. **pulchra**

Distribution:—hill and submontane belt.

Crepis pygmaea L.

Distribution:—subalpine and alpine belt.

N, # **Crepis sancta** (L.) Bornm. subsp. **nemausensis** (P.Fourn.) Babc.—NAT

Distribution:—common in the hill and submontane belt.

Crepis setosa Haller f.

Distribution:—hill, submontane and montane belt.

Crepis vesicaria L.

Distribution:—hill, submontane and montane belt.

Crupina crupinastrum (Moris) Vis.

Distribution:—between Raiano and Goriano Sicoli (Pirone & Tammaro 1997).

Crupina vulgaris Cass.

Distribution:—hill, submontane and montane belt.

***Dittrichia viscosa** (L.) Greuter subsp. **viscosa** (*Inula viscosa* (L.) Aiton subsp. *viscosa*)

Distribution:—S. Iona! (pers. obs.)

Doronicum columnae Ten.

Distribution:—montane and subalpine belt.

Echinops ritro L.—D

Distribution:—Velino (Montelucci 1958), Prati del Sirente (Tammaro et al. 1974). Not observed by us and perhaps confused in the past with *E. sphaerocephalus* subsp. *sphaerocephalus* , the only taxon belonging to the genus *Echinops* definitely present in the Park.

Echinops sphaerocephalus L. subsp. **sphaerocephalus**

Distribution:—hill, submontane and montane belt.

Erigeron acris L. subsp. **acris**

Distribution:—hill, submontane and montane belt.

Erigeron alpinus L.

Distribution:—subalpine and alpine belt.

N ***Erigeron annuus*** (L.) Desf.—NAT

Distribution:—Molina Aterno! (Conti et al. 2008a). The report by Conti et al. (2023a as *E. annuus* subsp. *strigosus*) for Molina Aterno should be mentioned here.

N ****Erigeron canadensis*** L.—INV

Distribution:—Tione along F. Aterno! (APP 28224).

Erigeron epiroticus (Vierh.) Halácsy

Distribution:—subalpine and alpine belt.

Erigeron glabratus Hoppe & Hornsch. ex Bluff & Fingerh.

Distribution:—subalpine and alpine belt.

N ***Erigeron sumatrensis*** Retz.—INV

Distribution:—hill and submontane belt.

Erigeron uniflorus L.

Distribution:—subalpine and alpine belt.

Eupatorium cannabinum L. subsp. ***cannabinum***

Distribution:—hill, submontane and montane belt.

Filago germanica (L.) Huds.

Distribution:—hill and submontane belt.

Galatella linosyris (L.) Rchb.f. subsp. ***linosyris*** (*Aster linosyris* (L.) Bernh.)

Distribution:—hill, submontane and montane belt.

N ****Galinsoga quadriradiata*** Ruiz & Pav.—CAS

Distribution:—Rocca di Mezzo!, Ovindoli! (APP 73283, 73530, 74210).

Gnaphalium uliginosum L.

Distribution:—Piana di Ovindoli! (Conti 1998; APP 66559), Val d'Arano! (Conti et al. 1999; APP 28589), Campo di Rovere! (APP 34789; 59912), Piana di San Nicola! (APP 62176, 62179).

N ***Helianthus pauciflorus*** Nutt.—CAS

Distribution:—Serra di Celano (Guarrera & Tammaro 1996).

N ***Helianthus tuberosus*** L.—NAT

Distribution:—hill and submontane belt.

Helichrysum italicum (Roth) G.Don subsp. ***italicum***

Distribution:—common in the hill, submontane and montane belt.

Helichrysum luteoalbum (L.) Rchb. (*Gnaphalium luteoalbum* L.; *Laphangium luteoalbum* (L.) Tzvelev)

Distribution:—Sirente, Valle Lupara (Groves 1880; Guarrera & Tammaro 1996).

Helminthotheca echioides (L.) Holub (*Picris echioides* L.)

Distribution:—hill and submontane belt.

E ***Hieracium acanthodontoides*** Arv.-Touv. & Belli—DD

Hieracium amplexicaule L. subsp. ***berardianum*** (Arv.-Touv.) Zahn

Hieracium bifidum Kit. ex Hornem. subsp. ***caesiiflorum*** (Almq. ex Norrl.) Zahn

E, # ***Hieracium bifidum*** Kit. ex Hornem. subsp. ***nummulariifolium*** Gottschl.

Hieracium bifidum Kit. ex Hornem. subsp. ***sinuosifrons*** (Dahlst.) Zahn

E	***Hieracium bifidum*** Kit. ex Hornem. subsp. ***subhastatum*** Gottschl.
E	***Hieracium bifidum*** Kit. ex Hornem. subsp. ***subimbricatum*** Gottschl.
E	***Hieracium bupleuroides*** C.C.Gmel. subsp. ***aprutiorum*** (Furrer & Zahn) Gottschl.
	Hieracium caesioides Arv.-Touv. subsp. ***caesioides***
	Hieracium caesioides Arv.-Touv. subsp. ***rionii*** (Gremli) Zahn
	Hieracium chlorelloides Zahn
E	***Hieracium chlorifolium*** Arv.-Touv. subsp. ***rendinaricum*** Gottschl.
	Hieracium chlorophyton Preissm. & Zahn ex Hayek
	Hieracium chondrillifolium Fr. subsp. ***boissieri*** (A.Huet & É.Huet ex Arv.-Touv.) Zahn
	Hieracium chondrillifolium Fr. subsp. ***megalocladum*** (Nägeli & Peter) Zahn
E	***Hieracium contii*** Gottschl.—DD
	Hieracium cydoniifolium Vill. subsp. ***mespilifolium*** (Arv.-Touv.) Zahn
	Hieracium dentatum Hoppe subsp. ***dentatiforme*** Nägeli & Peter
	Hieracium dentatum Hoppe subsp. ***subvillosum*** Nägeli & Peter
E	***Hieracium dentatum*** Hoppe subsp. ***trefferianiforme*** Gottschl.
E	***Hieracium dentatum*** Hoppe subsp. ***xanthostylophorum*** Furrer & Zahn
E	***Hieracium galeroides*** Gottschl. subsp. ***galeroides***
	Hieracium glaucinum Jord.—NC
	Hieracium huetianum Arv.-Touv.
	Hieracium humile Jacq. subsp. ***brachycaule*** Vuk. ex Zahn
E	***Hieracium hypochoeroides*** S.Gibson subsp. ***bifidopsis*** (Zahn) Greuter
	Hieracium hypochoeroides S.Gibson subsp. ***lithophilum*** (Arv.-Touv.) Greuter
E	***Hieracium hypochoeroides*** S.Gibson subsp. ***pallidopsis*** Gottschl.
E	***Hieracium hypochoeroides*** S.Gibson subsp. ***potamogetifolium*** Gottschl.
	Hieracium hypochoeroides S.Gibson subsp. ***prasinophyton*** (Zahn) Greuter
	Hieracium jurassicum Griseb. subsp. ***subperfoliatum*** (Arv.-Touv.) Greuter
E	***Hieracium lycopifolium*** Froel. subsp. ***ocreanum*** Gottschl.
	Hieracium murorum L. subsp. ***anisobasis*** Gottschl.
#	***Hieracium murorum*** L. subsp. ***pleiotrichum*** (Zahn) Zahn
	Hieracium naegelianum Pančić subsp. ***andreae*** (Degen & Zahn) Zahn
E	***Hieracium neoplatyphyllum*** Gottschl. subsp. ***malacofloccosum*** Gottschl.
	Hieracium neyranum Arv.-Touv. subsp. ***neyranum***
E, #	***Hieracium pallescens*** Waldst. & Kit. subsp. ***ciliatifolium*** (Zahn) Gottschl.
#	***Hieracium pellitum*** Fr. subsp. ***pellitum***
E	***Hieracium permaculatum*** Gottschl. subsp. ***permaculatum***
E	***Hieracium pietrae*** Zahn—DD
	Hieracium pilosum Schleich. ex Froel. subsp. ***portae*** (Nägeli & Peter) Gottschl.
#	***Hieracium pilosum*** Schleich. ex Froel. subsp. ***villosiceps*** Nägeli & Peter ex Gottschl.
	Hieracium pilosum Schleich. ex Froel. subsp. ***villosifolium*** (Nägeli & Peter) Greuter
E	***Hieracium prenanthoides*** Vill. subsp. ***lissocorium*** Furrer & Zahn
	Hieracium prenanthoides Vill. subsp. ***perfoliatum*** (Froel.) Fr.

Hieracium prenanthoides Vill. subsp. *prenanthoides*
E *Hieracium prenanthoides* Vill. subsp. *stupposifolium* Gottschl.
E *Hieracium profetanum* Belli
E *Hieracium pseudogrovesianum* Gottschl.—DD
Hieracium pulchellum Gren. ex Griseb.
Hieracium racemosum Waldst. & Kit. ex Willd. subsp. *alismatifolium* (Posp.) Zahn
E *Hieracium racemosum* Waldst. & Kit. ex Willd. subsp. *caramanicum* (Zahn) Zahn
Hieracium racemosum Waldst. & Kit. ex Willd. subsp. *crinitum* (Sm.) Rouy
E *Hieracium racemosum* Waldst. & Kit. ex Willd. subsp. *pulmonariifolium* Gottschl.
Hieracium racemosum Waldst. & Kit. ex Willd. subsp. *virgaurea* (Coss.) Zahn
Hieracium sabaudum L. subsp. *sublactucaceum* Zahn
Hieracium schmidtii Tausch subsp. *brunelliforme* (Arv.-Touv.) O.Bolòs &
E *Hieracium schmidtii* Tausch subsp. *crinitisquamum* Gottschl.
Hieracium scorzonerifolium Vill. subsp. *flexuosum* Waldst. & Kit. ex Nägeli & Peter
Hieracium symphytaceum Arv.-Touv. subsp. *neoprenanthes* (Arv.-Touv.) Zahn
Hieracium symphytaceum Arv.-Touv. subsp. *symphytaceum*
Hieracium tenuiflorum Arv.-Touv. subsp. *tenuiflorum*
Hieracium tomentosum L. subsp. *tomentosum*
Hieracium valdepilosum Vill. subsp. *subsinuatum* (Nägeli & Peter) Zahn
E *Hieracium venticaesum* Gottschl.
E *Hieracium villosum* Jacq. subsp. *doratophyllum* Nägeli & Peter
Hieracium villosum Jacq. subsp. *villosum*
Hypochaeris radicata L.
Distribution:—Forme (Lucchese & Lattanzi 1993).
Hypochaeris urens L. (*Hypochaeris cretensis* (L.) Bory & Chaub. subsp. *pinnatifida* (Ten.) P.Fourn.)
Distribution:—from montane to alpine belt.
E *Jacobaea alpina* (L.) Moench subsp. *samnitum* (Nyman) Peruzzi (*Senecio samniticus* Huet, *nom. inval.*; *Senecio samnitum* (Nyman) Greuter)—LC
Distribution:—Mt. Sirente (Groves 1880), Prati del Mt. Sirente (Guarrera & Tammaro 1996), Velino (Montelucci 1958).
Jacobaea erratica (Bertol.) Fourr. (*Senecio erraticus* Bertol.)
Distribution:—hill, submontane and montane belt.
Jacobaea erucifolia (L.) G.Gaertn., B.Mey. & Scherb. subsp. *erucifolia* (*Senecio erucifolius* L. subsp. *erucifolius*)
Distribution:—hill and submontane belt.
Jacobaea vulgaris Gaertn. subsp. *gotlandica* (Neuman) B.Nord. (*Senecio jacobaea* L. subsp. *gotlandicus* (Neuman) Sterner)—NT
Distribution:—Piani di Pezza! (Conti et al. 2012), Mt. Rotondo!, Prati della Madonna! (Roma-Marzio et al. 2016), Mt. delle Canelle!, Colle del Nibbio!, Mt. Briccialone! (Stinca et al. 2021a).

Jurinea mollis (L.) Rchb. subsp. ***mollis***
Distribution:—hill, submontane and montane belt.

Klasea lycopifolia (Vill.) Á.Löve & D.Löve (*Serratula lycopifolia* (Vill.) A.Kern.)—NT
Distribution:—Altopiano delle Rocche! (Conti & Manzi 1997; APP 28581, 28641, 28642, 28643, 59630), Piana di Ovindoli!, Campo di Rovere!, Prati del Sirente!, Campo Felice! (Stinca et al. 2021a; APP 28527, 28574, 28575, 28577, 28578, 67745).

Klasea nudicaulis (L.) Fourr. (*Serratula nudicaulis* (L.) DC.)
Distribution:—montane belt.

Lactuca perennis L.
Distribution:—hill, submontane and montane belt.

Lactuca saligna L.
Distribution:—hill, submontane and montane belt.

Lactuca sativa L. subsp. ***serriola*** (L.) Galasso, Banfi, Bartolucci & Ardenghi
Distribution:—hill and submontane belt.

Lactuca viminea (L.) J.Presl & C.Presl subsp. ***chondrilliflora*** (Boreau) St.-Lag.
Note:—previous reports as *L. viminea* (Groves 1880) and as *L. viminea* subsp. *viminea* (Lucchese & Lattanzi 1993; Petriccione 1993; Guarrera & Tammaro 1996) should be referred to this taxon.

Lactuca virosa L.
Distribution:—Rosciolo (Avena & Blasi 1975), Sirente over Ovindoli (Guarrera & Tammaro 1996).

Lapsana communis L. subsp. ***communis***
Distribution:—montane belt.

E, # ***Leontodon crispus*** Vill. var. ***biscutellifolius*** (DC.) F. Conti
Distribution:—common in stony pastures. For its relationships with similar taxa, see Conti et al. (2025b).

****Leontodon hispidus*** L. subsp. ***dubius*** (Hoppe) Pawłowska
Distribution:—Costa della Tavola! (APP 67601, 67602, 67603).

Leontodon hispidus L. subsp. ***hispidus***
Distribution:—hill, submontane to alpine belt.

E ****Leontodon intermedius*** (Fiori) Huter, Porta & Rigo—LC
Distribution:—Mt. Puzzillo!, above S. Maria in Valle Porclaneta! (APP 67601, 67601), Roccapreturo! (pers. obs.).

Leontodon rosanoi (Ten.) DC.
Distribution:—montane belt.

Leontodon saxatilis Lam. subsp. ***saxatilis***
Distribution:—hill and submontane belt.

Leontodon tuberosus L.
Distribution:—hill, submontane and montane belt.

Leucanthemum heterophyllum (Willd.) DC.
Distribution:—from montane to alpine belt.

****Leucanthemum ircutianum*** DC. subsp. ***leucolepis*** (Briq. & Cavill.) Vogt & Greuter

Distribution:—near S. Felice d'Ocre (C. Oberprieler in litt.).

\# ***Leucanthemum pallens*** (J.Gay ex Perreym.) DC.

Distribution:—hill, submontane and montane belt.

N ***Leucanthemum×superbum*** (Bergmans ex J.W.Ingram) D.H.Kent—CAS

Distribution:—Rocca di Mezzo! (APP 73526, 73527; Conti et al. in press).

Leucanthemum vulgare (Vaill.) Lam. subsp. ***vulgare***

Distribution:—hill, submontane and montane belt.

E ***Lophiolepis lobelii*** (Ten.) Del Guacchio, Bureš, Iamonico & P.Caputo (*Cirsium lobelii* Ten.)—DD

Distribution:—common in overgrazed areas.

E, # ***Lophiolepis tenoreana*** (Petr.) Del Guacchio, Bureš, Iamonico & P.Caputo (*Cirsium tenoreanum Petr.*)—LC

Distribution:—common in overgrazed areas.

Matricaria chamomilla L.

Distribution:—hill, submontane and montane belt.

N ***Matricaria discoidea*** DC. subsp. ***discoidea*** —NAT

Distribution:—montane belt.

\# ***Mycelis muralis*** (L.) Dumort. subsp. ***muralis***

Distribution:—hill, submontane and montane belt.

\# ***Omalotheca diminuta*** (Braun-Blanq.) Bartolucci & Galasso (*Gnaphalium diminutum* Braun-Blanq.; *Gnaphalium hoppeanum* W.D.J.Koch subsp. *magellense* (Fiori) Strid

Distribution:—subalpine and alpine belt.

\# ***Omalotheca sylvatica*** (L.) Sch.Bip. & F.W.Schultz (*Gnaphalium sylvaticum* L.)

Distribution:—montane to subalpine belt.

Onopordum acanthium L. subsp. ***acanthium***

Distribution:—hill, submontane and montane belt.

\# ***Onopordum illyricum*** L. subsp. ***illyricum***

Distribution:—hill, submontane and montane belt.

Pallenis spinosa (L.) Cass. subsp. ***spinosa***

Distribution:—hill and submontane belt.

****Pentanema britannicum*** (L.) D.Gut.Larr., Santos-Vicente, Anderb., E.Rico & M.M.Mart.Ort. (*Inula britannica* L.)

Distribution:—S. Iona!, Conche di Ovindoli!, Rocca di Mezzo! (pers. obs.).

****Pentanema hirtum*** (L.) D.Gut.Larr., Santos-Vicente, Anderb., E.Rico & M.M.Mart.Ort.

Distribution:—between Campo di Rovere and l'Anatella! (pers. obs.).

\# ***Pentanema montanum*** (L.) D.Gut.Larr., Santos-Vicente, Anderb., E.Rico & M.M.Mart.Ort. (*Inula montana* L.)

Distribution:—hill, submontane and montane belt.

Pentanema salicinum (L.) D.Gut.Larr., Santos-Vicente, Anderb., E.Rico & M.M.Mart.Ort. (*Inula salicina* L.)

Distribution:—hill, submontane and montane belt.

****Pentanema spiraeifolium*** (L.) D.Gut.Larr., Santos-Vicente, Anderb., E.Rico & M.M.Mart.Ort. (*Inula spiraeifolia* L.)

Distribution:—Mt. Urano (southern slopes between Castel di Ieri and Goriano Sicoli)! (pers. obs.).

\# ***Pentanema squarrosum*** (L.) D.Gut.Larr., Santos-Vicente, Anderb., E.Rico & M.M.Mart.Ort. (*Inula conyzae* (Griess.) DC.)

Distribution:—hill, submontane and montane belt.

Petasites hybridus (L.) G.Gaertn., B.Mey. & Scherb. subsp. ***hybridus***

Distribution:—hill, submontane and montane belt.

Picnomon acarna (L.) Cass.

Distribution:—hill and submontane belt.

\# ***Picris hieracioides*** L. subsp. ***hieracioides***

Distribution:—common in the hill, submontane and montane belt.

****Picris hieracioides*** L. subsp. ***umbellata*** (Schrank) Ces.

Distribution:—Neviera! (APP 73394, 73395), Val d'Arano! (pers. obs.).

E ***Pilosella cepitina*** (Gottschl.) Gottschl.—DD

\# ***Pilosella cymosa*** (L.) F.W.Schultz & Sch.Bip. subsp. ***sabina*** (Sebast.)

Pilosella hoppeana (Schult.) F.W.Schultz & Sch.Bip. subsp. ***macrantha*** (Ten.) S.Bräut. & Greuter

Pilosella hypeurya (Peter) Soják

\# ***Pilosella lactucella*** (Wallr.) P.D.Sell & C.West subsp. ***nana*** (Scheele) M.Laínz

\# ***Pilosella officinarum*** F.W.Schultz & Sch.Bip.

\# ***Pilosella piloselloides*** (Vill.) Soják

Pilosella tubulata (Vollm.) Soják

Pilosella visianii F.W.Schultz & Sch.Bip.

Pilosella ziziana (Tausch) F.W.Schultz & Sch.Bip.

\# ***Prenanthes purpurea*** L.

Distribution:—montane belt.

Pseudopodospermum hispanicum (L.) Zaika, Sukhor. & N.Kilian subsp. ***asphodeloides*** (Wallr.) Bartolucci, Galasso & F.Conti (*Scorzonera glastifolia* auct. Fl. Ital.; *Scorzonera hispanica* L. subsp. *asphodeloides* (Wallr.) Arcang.)

Distribution:—montane belt.

\# ***Ptilostemon strictus*** (Ten.) Greuter

Distribution:—hill, submontane and montane belt.

Pulicaria dysenterica (L.) Bernh.

Distribution:—hill, submontane and montane belt.

Reichardia picroides (L.) Roth

Distribution:—hill and submontane belt.

Rhagadiolus stellatus (L.) Gaertn.

Distribution:—hill and submontane belt.

\# ***Robertia taraxacoides*** (Loisel.) DC.—LC

Distribution:—montane to alpine belt.

Scolymus hispanicus L. subsp. ***hispanicus***

Distribution:—hill and submontane belt.

Scorzonera cana (C.A.Mey.) Griseb.

Distribution:—hill, submontane and montane belt.

Scorzonera laciniata L. subsp. *laciniata*—D

Distribution:—Piani di Pezza (Ciaschetti et al. 2006). Not confirmed by us and probably to be attribute to *S. cana.*

Scorzonera laciniata L. subsp. *decumbens* (Guss.) Greuter—D

Distribution:—Velino (Tenore 1831).

Scorzonera purpurea L.

Distribution:—Campo Felice (De Santis & Soldati 2011).

Scorzoneroides autumnalis (L.) Moench

Distribution:—montane belt.

Scorzoneroides cichoriacea (Ten.) Greuter

Distribution:—montane belt.

E, # ***Scorzoneroides montana*** (Lam.) Holub subsp. ***breviscapa*** (DC.) Greuter—LC

Distribution:—Velino! (Tenore 1830, 1831; Bertoloni 1833-54; Martelli 1904; Montelucci 1958; Petriccione 1993, 1994). The reports for Prati del Sirente (Tammaro et al. 1974), Piani di Canale (Veri & Tammaro 1980) and the lower parts of Mt. Sirente (680-700 m) are unlikely, as the plant lives in high-altitude scree.

Senecio doronicum (L.) L. subsp. ***orientalis*** J.Calvo

Distribution:—montane to subalpine belt.

N, # ***Senecio inaequidens*** DC.—INV

Distribution:—hill, submontane and montane belt.

E, # ***Senecio apenninus*** Tausch—LC

Distribution:—Mt. Velino (Tenore 1831; Martelli 1904), Valle del Puzzillo, Macchia Rotonda (Lucchese & Lattanzi 1993).

E, # ***Senecio ovatus*** (G.Gaertn., B.Mey. & Scherb.) Willd. subsp. ***stabianus*** (Lacaita) Greuter—LC

Distribution:—common in clearings or in open beech forests

Senecio rupestris Waldst. & Kit.

Distribution:—from montane to alpine belt.

Senecio scopolii Hoppe & Hornsch.—NT

Distribution:—from montane to alpine belt.

E, # ***Senecio tenorei*** Pignatti—LC

Distribution:—Mt. Velino (Tenore 1830; Martelli 1904), Valle di Sevice, Mt. Rozza (Lucchese & Lattanzi 1993). The report for Campo Felice (De Santis & Soldati 2011, 2019) should to be referred to *S. scopolii* after examination on published photos and our findings in APP (30127).

Senecio vulgaris L. subsp. ***vulgaris***

Distribution:—hill, submontane and montane belt.

Serratula tinctoria L.

Distribution:—Altopiano delle Rocche!, Campo di Rovere!, Piana di Ovindoli! (APP 28663, 28664, 59538, 59539, 59631, 59632, 66539, 66612, 66779, 68009). If its autonomy were recognised, it would refer to *S. tinctoria* subsp. *monticola* (Boreau) Berher.

Silybum marianum (L.) Gaertn.

Distribution:—hill and submontane belt.

Solidago virgaurea L. subsp. ***virgaurea***

Distribution:—montane to subalpine belt.

Sonchus arvensis L. subsp. ***arvensis***

Distribution:—from Ajelli to Ovindoli, Mt. Ventrino (Guarrera & Tammaro

Sonchus asper (L.) Hill subsp. ***asper***

Distribution:—hill and submontane belt.

****Sonchus bulbosus*** (L.) N.Kilian & Greuter subsp. ***bulbosus***

Distribution:—Gole di S. Venanzio near Raiano! (pers. obs.).

Sonchus oleraceus L.

Distribution:—hill and submontane belt.

A ***Tanacetum balsamita*** L.—CAS

Distribution:—Terranera! (Conti et al. 2008; APP 27582, 27583, 27584).

Tanacetum corymbosum (L.) Sch.Bip. subsp. ***achilleae*** (L.) Greuter

Distribution:—montane and subalpine belt.

Tanacetum parthenium (L.) Sch.Bip.

Distribution:—montane belt.

****Tanacetum vulgare*** L. subsp. ***vulgare***

Distribution:—between Tione and Fontecchio! (APP 33726).

E, # ***Taraxacum apenninum*** (Ten.) DC.—LC

Distribution:—Mt. Sirente! (Groves 1880; Furnkranz 1964; Guarrera & Tammaro 1996; APP 15551), Velino! (Petriccione 1993, 1994, 2005).

Taraxacum sect. ***Erythrosperma*** (H.Lindb.) Dahlst.

Note:—*T. fulvum* Raunk. has also been reported for Campo Felice (De Santis & Soldati 2011), but we include it in this section pending a review of the group.

Taraxacum sect. ***Palustria*** (H.Lindb.) Dahlst.

Distribution:—Campo Felice! (Ciaschetti et al. 2024; APP 30137), Campo di Rovere!, Rocca di Mezzo !, Prati della Madonna!, Inghiottitoio di Terranera! (APP 34818, 34819, 51568, 51571, 59767, 59768, 59769, 59770, 59771, 59772; pers. obs.).

Taraxacum F.H.Wigg. sect. ***Taraxacum***

Distribution:—common in the hill, submontane and montane belt.

E ***Taraxacum vaccarii*** Soest—DD

Distribution:—Ajelli in the meadows of S. Maria (Soest van 1966).

Tephroseris integrifolia (L.) Holub subsp. *capitata* (Wahlenb.) B.Nord.—NC

Distribution:—Velino (Abbate 1903).

Tephroseris integrifolia (L.) Holub subsp. ***integrifolia***

Distribution:—montane belt.

Tragopogon crocifolius L. subsp. ***crocifolius***

Distribution:—montane belt.

Tragopogon dubius Scop.

Distribution:—montane belt.

Tragopogon eriospermus Ten.

Distribution:—S. Spirito d'Ocre!, Le Prata!, between Rocca di Mezzo and Piani di Pezza! (APP 32333, 32334, 59604, 66664, 66752), Collarmele! (pers. obs.), Valle Cerchiata (Avena & Blasi 1975).

Tragopogon minor Mill.

Distribution:—Velino (Petriccione 1993).
Tragopogon porrifolius L.
Distribution:—hill, submontane and montane belt.
Tragopogon pratensis L.
Distribution:—montane belt.
Tragopogon samaritanii Heldr. & Sartori ex Boiss.
Distribution:—hill, submontane and montane belt.
Tripleurospermum inodorum (L.) Sch.Bip.
Distribution:—montane belt.
Tussilago farfara L.
Distribution:—hill, submontane and montane belt.
****Tyrimnus leucographus*** (L.) Cass.
Distribution:—Castelvecchio Subequo! (APP 73489).
Urospermum dalechampii (L.) Scop. ex F.W.Schmidt
Distribution:—hill and submontane belt.
Urospermum picroides (L.) Scop. ex F.W.Schmidt
Distribution:—hill and submontane belt.
N ***Xanthium orientale*** L.—NAT
Distribution:—hill, submontane and montane belt.
N, # ***Xanthium spinosum*** L.—CAS
Distribution:—montane belt.
Xanthium strumarium L.
Distribution:—Serra di Celano, surroundings of Goriano Valli (Guarrera & Tammaro 1996).
Xeranthemum cylindraceum Sm.
Distribution:—hill, submontane and montane belt.
Xeranthemum inapertum (L.) Mill.
Distribution:—hill, submontane and montane belt.

BERBERIDACEAE

Berberis vulgaris L.
Distribution:—hill, submontane and montane belt.
N ****Mahonia aquifolium*** (Pursh) Nutt.—CAS
Distribution:—Alba Fucens (pers. obs.).

BETULACEAE

Colt, # ****Alnus cordata*** (Loisel.) Duby
Distribution:—Piè Cafornia (pers. obs.)
Alnus glutinosa (L.) Gaertn.
Distribution:—hill and submontane belt.
Betula pendula Roth
Distribution:—montane belt.
Carpinus betulus L.
Distribution:—hill, submontane and montane belt.
Carpinus orientalis Mill. subsp. ***orientalis***

Distribution:—hill and submontane belt.

\# ***Corylus avellana*** L.

Distribution:—hill, submontane and montane belt.

\# ***Ostrya carpinifolia*** Scop.

Distribution:—common in the hill, submontane and montane belt.

BORAGINACEAE

Aegonychon purpurocaeruleum (L.) Holub (*Buglossoides purpurocaerulea* (L.) I.M.Johnst.; *Lithospermum purpurocaeruleum* L.)

Distribution:—hill and submontane belt.

Anchusa azurea Mill.

Distribution:—hill, submontane and montane belt.

Anchusa undulata L. subsp. ***hybrida*** (Ten.) Bég.

Distribution:—hill and submontane belt.

\# ***Asperugo procumbens*** L.

Distribution:—hill, submontane and montane belt.

Borago officinalis L.

Distribution:—hill, submontane and montane belt.

\# ***Buglossoides arvensis*** (L.) I.M.Johnst. subsp. ***arvensis***

Distribution:—

Buglossoides incrassata (Guss.) I.M.Johnst. subsp. ***incrassata***

Distribution:—Velino (Tenore 1830, 1831), Fonte Canale (Lucchese & Lattanzi 1993), Carrito! (pers. obs.).

Cerinthe major L. subsp. ***major***

Distribution:—hill and submontane belt.

Cerinthe minor L. subsp. ***auriculata*** (Ten.) Domac

Distribution:—montane belt.

E ***Cynoglossum apenninum*** L.—LC

Distribution:—common in clearings and forest edges.

****Cynoglossum cheirifolium*** L.

Distribution:—Gole di S. Venanzio! (pers. obs.).

Cynoglossum columnae Ten.

Distribution:—montane belt.

\# ***Cynoglossum creticum*** Mill.

Distribution:—hill, submontane and montane belt.

E, # ***Cynoglossum magellense*** Ten.—LC

Distribution:—common in mountain and subalpine pastures.

\# ***Cynoglossum montanum*** L.

Distribution:—hill, submontane and montane belt.

Cynoglossum officinale L.

Distribution:—hill, submontane and montane belt.

\# ***Cynoglottis barrelieri*** (All.) Vural & Kit Tan subsp. ***barrelieri***

Distribution:—montane belt.

Echium italicum L. subsp. ***italicum***

Distribution:—hill and submontane belt.

Echium plantagineum L.
Distribution:—hill and submontane belt.
Echium vulgare L.
Distribution:—hill, submontane and montane belt.
Lappula squarrosa (Retz.) Dumort.
Distribution:—hill, submontane and montane belt.
Lithospermum officinale L.
Distribution:—Forme (Lucchese & Lattanzi 1993), Lago di Civita! (APP 34847).

\# ***Myosotis arvensis*** (L.) Hill subsp. ***arvensis***
Distribution:—hill, submontane and montane belt.

E ***Myosotis decumbens*** Host subsp. ***florentina*** Grau—DD
Distribution:—above Secinaro, Prati del Sirente (Guarrera & Tammaro 1996), Mt. Ocre in Vallone Canavine! (APP 15343).

E, # ***Myosotis graui*** Selvi (*Myosotis ambigens* auct. Fl. Ital.)—LC
Distribution:—very common on all reliefs.

\# ***Myosotis incrassata*** Guss.
Distribution:—hill, submontane and montane belt.
Myosotis laxa Lehm. subsp. ***cespitosa*** (Schultz) Hyl. ex Nordh.
Distribution:—montane belt.
Myosotis minutiflora Boiss. & Reut. subsp. ***minutiflora***
Distribution:— Mt. Briccialone!, Mt. Cefalone! (Conti et al. 2023a; APP 67171, 67389, 67390, 67391, 67440, 67441).

\# ***Myosotis nemorosa*** Besser
Distribution:—Valle Pioppi (Lucchese & Lattanzi 1993), Velino (Petriccione 1993).

\# ***Myosotis ramosissima*** Rochel subsp. ***ramosissima***
Distribution:—montane belt.
Myosotis scorpioides L. subsp. ***scorpioides***
Distribution:—montane belt.

\# ***Myosotis speluncicola*** (Boiss.) Rouy—LC
Distribution:—Valle Majelama (Pignatti 1982), V.ne di Sevice (Lucchese & Lattanzi 1993), Mt. Ocre! (Conti et al. 2016; APP 55049), Grotta del Cervo! (APP 73367).
Myosotis stricta Link ex Roem. & Schult.
Distribution:—montane belt.

\# ****Myosotis sylvatica*** Hoffm. subsp. ***cyanea*** (Hayek) Vestergren—DD
Distribution:—Rif. La Vecchia!, from Chalet Mt. Sirente towards the summit!, valley below Costa della Tavola!, Mt. delle Canelle!, Cimata di Pezza! (APP 15636, 67618, 67619, 67898, 67904, 67913, 72800, 72842), Velino (Petriccione 1993 as *M. sylvatica* subsp. *sylvatica*). Other reports of *M. sylvatica* subsp. *sylvatica* (Mt. Sirente, Valle Cerchiata, Velino) (Veri & Tammaro 1980; Lucchese & Lattanzi 1993; Petriccione 1993) are also likely to refer to this taxon.

E ****Onosma echioides*** (L.) L. subsp. ***angustifolia*** (Lehm.) Peruzzi & N.G.Passal.—DD

Distribution:—loc. Macchione near Goriano Sicoli!, near Castelvecchio Subequo! (APP 29897, 73020).

E, # ***Onosma echioides*** (L.) L. subsp. ***echioides*** —LC
Distribution:—common on rocky slopes at the base of mountains.

Pulmonaria hirta L.
Distribution:—hill, submontane and montane belt.

****Pulmonaria officinalis*** L. subsp. ***officinalis***
Distribution:—Mt. Ocre in Vallone Canavine!, beech wood above Chalet Sirente! (APP 15369, 15618).

****Symphytum bulbosum*** K.F.Schimp.
Distribution:— along F.Aterno between Monticchio and Fossa!, near Rosciolo! (pers. obs.).

Symphytum officinale L.
Distribution:—Castelnuovo in loc. Vicenna! (Conti et al. 2008a; APP 36567, 36568).

Symphytum tuberosum L.
Distribution:—hill, submontane and montane belt.

BRASSICACEAE

Aethionema saxatile (L.) W.T.Aiton
Distribution:—hill, submontane and montane belt.

Alliaria petiolata (M.Bieb.) Cavara & Grande
Distribution:—hill, submontane and montane belt.

Alyssoides utriculata (L.) Medik. subsp. ***utriculata***
Distribution:—hill, submontane and montane belt.

Alyssum alyssoides (L.) L.
Distribution:—hill, submontane and montane belt.

E, # ***Alyssum diffusum*** Ten. subsp. ***diffusum*** —LC
Distribution:—common in rocky slopes.

Alyssum simplex Rudolphi
Distribution:—hill, submontane and montane belt.

Arabidopsis thaliana (L.) Heynh.
Distribution:—hill, submontane and montane belt.

Arabis alpina L. subsp. ***alpina***
Distribution:—alpine belt.

Arabis alpina L. subsp. ***caucasica*** (Willd.) Briq.
Distribution:—hill, submontane to subalpine belt.

Arabis auriculata Lam.
Distribution:—hill, submontane and montane belt.

Arabis bellidifolia Crantz subsp. ***stellulata*** (Bertol.) Greuter & Burdet
Distribution:—Velino! (Conti 1998).

Arabis ciliata Clairv.
Distribution:—Piani di Pezza, Mt. Costone (Lucchese & Lattanzi 1993).

Arabis collina Ten. subsp. ***collina***
Distribution:—hill, submontane and montane belt.

E *__Arabis collina__* Ten. subsp. *__rosea__* (DC.) Minuto—NT

Distribution:—Gole di S. Venanzio near the Eremo! (pers. obs.).

__Arabis hirsuta__ (L.) Scop.

Distribution:—hill, submontane and montane belt.

__Arabis sagittata__ (Bertol.) DC.

Distribution:—hill, submontane and montane belt.

__Arabis surculosa__ N.Terracc.

Distribution:—Sirente (Groves 1880), Mt. Bicchero (Lucchese & Lattanzi 1993).

*__*Arabis verna__* (L.) W.T.Aiton

Distribution:—Gole di S. Venanzio! (APP 35695).

E *__Aubrieta columnae__* Guss. subsp. *__columnae__*—NT

Distribution:—Mt. Sirente! (Groves 1880; APP 36627), Gole di Celano! (Fiori et al. 1907; Pomponi et al. 1988; Frizzi et al. 1996; APP 66986), Valle Lupara, Mt. Ceresole, Mt. Canale (Tammaro 1971; Guarrera & Tammaro 1996), Mt. Briccialone!, Costa della Tavola!, Serra di Celano! (APP 67387, 67610, 67655).

__Barbarea bracteosa__ Guss.

Distribution:—montane belt.

__Barbarea sicula__ C.Presl—NT

Distribution:—Piani di Pezza!, Prati della Madonna!, Rocca di Mezzo!, berween Rocca di Mezzo and Terranera!, Campo di Rovere!, Campo Felice! (Conti et al. 2023a; APP 59530, 59838, 64949, 65124, 66734, 68098, 68099, 68113, 72901).

__Barbarea vulgaris__ W.T.Aiton

Distribution:—hill, submontane and montane belt.

Biscutella cichoriifolia Loisel.

Distribution:—Sirente, Vado di S. Venanzio (Groves 1880; Guarrera & Tammaro 1996 from a specimen collected by Profeta and kept in FI).

E, # *__Biscutella laevigata__* L. subsp. *__australis__* Raffaelli & Baldoin—LC

Distribution:—Mt. Sirente, Gagliano Aterno, Rosciolo (Raffaelli & Baldoin 1997 from specimens collected by Groves, Levier e Martelli), Val Lupara! (APP

__Biscutella laevigata__ L. subsp. *__laevigata__*

Distribution:—hill, submontane to subalpine belt.

__Brassica gravinae__ Ten.

Distribution:—subalpine to alpine belt.

__Brassica rapa__ L.

Distribution:—recorded in Lucoli outside the park but near its boundaries (De Santis & Soldati 2019).

__Bunias erucago__ L.

Distribution:—hill and submontane belt.

*__*Calepina irregularis__* (Asso) Thell.

Distribution:—at foot of Mt. Ventrino! (APP 72996)

__Capsella bursa-pastoris__ (L.) Medik. subsp. *__bursa-pastoris__*

Distribution:—hill, submontane and montane belt.

__Capsella rubella__ Reut.

Distribution:—hill, submontane and montane belt.

*__*Cardamine amporitana__* Sennen & Pau

Distribution:—Le Prata!, Campo di Rovere!, F. Aterno at Ponte Romano near S. Maria della Vittoria!, Lago di Molina Aterno! (APP 1621, 34810, 73030, 73038).

\# ***Cardamine bulbifera*** (L.) Crantz
Distribution:—montane belt.

Cardamine chelidonia L.
Distribution:—montane belt.

\# ***Cardamine enneaphyllos*** (L.) Crantz
Distribution:—montane belt.

\# ***Cardamine graeca*** L.
Distribution:—hill, submontane and montane belt.

Cardamine heptaphylla (Vill.) O.E.Schulz
Distribution:—montane belt.

Cardamine hirsuta L.
Distribution:—hill, submontane and montane belt.

Cardamine impatiens L. subsp. ***impatiens***
Distribution:—montane belt.

Cardamine kitaibelii Bech.
Distribution:—montane belt.

Cardamine parviflora L.
Distribution:—wet meadows of Ovindoli (Guarrera & Tammaro 1996).

Clypeola jonthlaspi L.
Distribution:—hill, submontane and montane belt.

****Conringia austriaca*** (Jacq.) Sweet
Distribution:—Collarmele! (pers. obs.).

A ****Conringia orientalis*** (L.) Andrz. ex DC.—NAT
Distribution:— Collarmele! (pers. obs.).

Descurainia sophia (L.) Webb ex Prantl
Distribution:—montane belt.

Diplotaxis erucoides (L.) DC. subsp. ***erucoides***
Distribution:—hill, submontane and montane belt.

Diplotaxis muralis (L.) DC.
Distribution:—hill and submontane belt.

Diplotaxis tenuifolia (L.) DC.
Distribution:—hill, submontane and montane belt.

\# ***Draba aizoides*** L. subsp. ***aizoides***
Distribution:—montane to alpine belt.

Draba verna L. subsp. ***praecox*** (Steven) Rouy & Foucaud
Distribution:—hill, submontane and montane belt.

\# ***Draba verna*** L. subsp. ***verna***
Distribution:—hill, submontane and montane belt.

Drabella muralis (L.) Fourr. (*Draba muralis* L.)
Distribution:—hill, submontane and montane belt.

C ***Eruca vesicaria*** (L.) Cav.
Distribution:—hill, submontane and montane belt.

E ***Erysimum apenninum*** Peccenini & Polatschek—LC

Distribution:—hill, submontane and montane belt.

A *__Erysimum cheiranthoides__* L.—NAT

Distribution:—Prati del Sirente (Guarrera & Tammaro 1996), Val d'Arano! (Conti 1998; APP 28623, 28624, 28625), Conche di Ovindoli! (APP 66563).

A *__Erysimum cheiri__* (L.) Crantz—NAT

Distribution:—hill, submontane and montane belt.

E, # *__Erysimum majellense__* Polatschek—LC

Distribution:—rocky slopes at high altitudes.

E, # *__Erysimum pseudorhaeticum__* Polatschek—LC

Distribution:—common on rocky slopes.

N *__Erysimum repandum__* L.—CAS

Distribution:—Mt. Sirente (Groves 1880).

__Fibigia clypeata__ (L.) Medik. subsp. *__clypeata__*

Distribution:—hill and submontane belt.

__Fourraea alpina__ (L.) Greuter & Burdet (*Arabis brassica* (Leers) Rauschert)

Distribution:—montane belt.

__Hesperis laciniata__ All. subsp. *__laciniata__*

Distribution:—hill, submontane and montane belt.

__Hesperis matronalis__ L. subsp. *__matronalis__*

Distribution:—Sirente, Mt. Ventrino (Groves 1880; Guarrera & Tammaro 1996), S. Spirito d'Ocre! (APP 32331).

__Hornungia alpina__ (L.) O.Appel subsp. *__alpina__*

Distribution:—from montane to alpine belt.

__Hornungia petraea__ (L.) Rchb. subsp. *__petraea__*

Distribution:—montane belt.

__Iberis saxatilis__ L. subsp. *__saxatilis__*

Note:—the report of *I. sempervirens* for Gole di San Venanzio (Conti 1993) is incorrect; it refers to a population with atypical individuals, found at low altitude and attributable to this taxon.

__Iberis violacea__ W.T.Aiton

Distribution:—montane to alpine belt.

__Isatis apennina__ Ten. ex Grande

Distribution:—subalpine to alpine belt.

A,# *__Isatis tinctoria__* L. subsp. *__tinctoria__*—INV

Distribution:—hill and submontane belt.

__Kernera saxatilis__ (L.) Sweet subsp. *__saxatilis__*

Distribution:—alpine belt.

__Lepidium campestre__ (L.) W.T.Aiton

Distribution:—hill, submontane and montane belt.

***Lepidium coronopus__* (L.) Al-Shehbaz

Distribution:—Piano di S. Nicola! (APP 62174).

N *__Lepidium didymum__* L.—NAT

Distribution:—Rovere, Ovindoli (Viegi et al. 1990).

__Lepidium draba__ L. subsp. *__draba__*

Distribution:—hill and submontane belt.

Lepidium graminifolium L. subsp. ***graminifolium***
Distribution:—hill and submontane belt.
AL ****Lobularia maritima*** (L.) Desv.—CAS
Distribution:—Rovere! (pers. obs.).
Lunaria annua L.
Distribution:—hill, submontane and montane belt.
Lunaria rediviva L.
Distribution:—hill, submontane and montane belt.
****Malcolmia orsiniana*** (Ten.) Ten. subsp. ***orsiniana***
Distribution:—Mt. Cefalone! (APP).
Matthiola fruticulosa (L.) Maire subsp. ***fruticulosa***
Distribution:—hill and submontane belt.
\# ***Matthiola fruticulosa*** (L.) Maire subsp. ***valesiaca*** (Boiss.) P.W.Ball
Distribution:—from montane to alpine belt.
\# ***Microthlaspi perfoliatum*** (L.) F.K.Mey. (*Thlaspi perfoliatum* L.)
Distribution:—hill, submontane and montane belt.
****Mummenhoffia alliacea*** (L.) Esmailbegi & Al-Shehbaz (*Thlaspi alliaceum* L.)
Distribution:—between Rocca di Mezzo and Piani di Pezza (APP 66749).
Mutarda arvensis (L.) D.A.German (*Sinapis arvensis* L.)
Distribution:—Mt. Ventrino (Guarrera & Tammaro 1996).
C ****Mutarda nigra*** (L.) Bernh. (*Brassica nigra* (L.) W.D.J.Koch)
Distribution:—Molina Aterno! (APP 28386).
Myagrum perfoliatum L.
Distribution:—hill, submontane and montane belt.
Nasturtium officinale W.T.Aiton
Distribution:—F. Aterno (Groves 1880), Gole di Celano (Frizzi et al. 1996; Guarrera & Tammaro 1996).
****Neslia paniculata*** (L.) Desv. subsp. ***thracica*** (Velen.) Bornm.
Distribution:—Forme!, Collarmele! (pers. obs.).
Noccaea brachypetala (Jord.) F.K.Mey.
Distribution:—montane to subalpine belt.
\# ***Noccaea praecox*** (Wulfen) F.K.Mey.
Distribution:—montane belt.
E, # ***Noccaea stylosa*** (Ten.) Rchb.—LC
Distribution:—pastures and hayfields in the subalpine plain.
E, # ***Noccaea torreana*** (Ten.) Bartolucci, Galasso & Peruzzi—DD
Distribution:—Mt. Puzzillo (Lucchese & Lattanzi 1993), Velino (Petriccione 1993).
E ***Phyllolepidum rupestre*** (Sweet) Trinajstić (*Alyssum rupestre* Ten.; *Ptilotrichum cyclocarpum* auct.)—NT
Distribution:—Mt. Sirente! (Groves 1880; Guarrera & Tammaro 1996; pers.
Pseudoturritis turrita (L.) Al-Shehbaz
Distribution:—hill, submontane and montane belt.
Rapistrum rugosum (L.) All.
Rorippa amphibia (L.) Besser—NC

Distribution:—Mt. Sirente (Parlatore 1848-96).
Rorippa sylvestris (L.) Besser subsp. ***sylvestris***
Distribution:—hill, submontane and montane belt.
Sisymbrium austriacum Jacq. subsp. ***austriacum***
Distribution:—Velino (Petriccione 1993).
****Sisymbrium irio*** L.
Distribution:—Castel di Ieri!, Castelvecchio Subequo!, Massa d'Albe! (pers.
Sisymbrium officinale (L.) Scop.
Distribution:—hill, submontane and montane belt.
C ***Sisymbrium orientale*** L.
Distribution:—hill, submontane and montane belt.
Thlaspi arvense L.
Distribution:—montane belt.
Turritis glabra L.
Distribution:—hill, submontane and montane belt.

BUXACEAE
Buxus sempervirens L.
Distribution:—hill and submontane belt.

CAMPANULACEAE
Campanula bononiensis L.
Distribution:—montane belt.
Campanula cochleariifolia Lam.
Distribution:—subalpine and alpine belt.
****Campanula erinus*** L.
Distribution:—Molina Aterno!, Rosciolo! (pers. obs.).
Campanula foliosa Ten.
Distribution:—montane belt.
E, # ***Campanula fragilis*** Cirillo subsp. ***cavolinii*** (Ten.) Damboldt—LC
Distribution:—common in low altitude cliffs.
Campanula glomerata L.
Distribution:—montane belt.
Campanula latifolia L.
Distribution:—Gole di Celano! (Frizzi et al. 1996; Guarrera & Tammaro 1996; APP 9460), Val d'Arano! (pers. obs.).
E ***Campanula micrantha*** Bertol.—LC
Distribution:—widespread in stony pastures.
Campanula persicifolia L. subsp. ***persicifolia***
Distribution:—montane belt.
AL ****Campanula rapunculoides*** L. subsp. ***rapunculoides***—CAS
Distribution:—Rocca di Mezzo! (APP 73531).
Campanula rapunculus L.
Distribution:—hill, submontane and montane belt.

E, # **_Campanula scheuchzeri_** Vill. subsp. **_pseudostenocodon_** (Lacaita) Bernardo, Gargano & Peruzzi—LC

Distribution:—Valle di Sevice (Lucchese & Lattanzi 1993), Velino (Bernardo et al. 2005), Campo Felice (De Santis & Soldati 2011), Mt. Rozza! (APP 32493, 32494).

Campanula scheuchzeri Vill. subsp. **_scheuchzeri_**

Distribution:—common from the montane to alpine belt.

E, # **_Campanula tanfanii_** Podlech—LC

Distribution:—common on the cliffs.

Campanula trachelium L. subsp. **_trachelium_**

Distribution:—hill, submontane and montane belt.

Edraianthus graminifolius (L.) A.DC. ex Meisn. subsp. **_graminifolius_**

Distribution:—from montane to alpine belt.

*Legousia hybrida (L.) Delarbre

Distribution:—S. Benedetto in Perillis!, Prati del Sirente!, Goriano Sicoli! (APP 6125, 27779, 73499).

Legousia speculum-veneris (L.) Chaix subsp. **_speculum-veneris_**

Distribution:—hill and submontane belt.

Phyteuma hemisphaericum L.—NC

Distribution:—Mt. Sirente (Groves 1880).

Phyteuma orbiculare L.

Distribution:—from montane to alpine belt.

CANNABACEAE

*Celtis australis L. subsp. **_australis_**

Distribution:—Fontecchio!, Acciano! (APP 74178, 74219).

Humulus lupulus L.

Distribution:—hill and submontane belt.

CAPRIFOLIACEAE

Lonicera alpigena L. subsp. **_alpigena_**

Distribution:—montane belt.

Lonicera caprifolium L.

Distribution:—hill, submontane and montane belt.

Lonicera etrusca Santi

Distribution:—hill and submontane belt.

Lonicera implexa Aiton subsp. **_implexa_**

Distribution:—Gole di S. Venanzio (D'Errico 1936; Montelucci 1971).

Lonicera xylosteum L.

Note:—only var. _nigra_ Loisel. is reported for the central Apennines (hill, submontane and montane belt).

CARYOPHYLLACEAE

A **_Agrostemma githago_** L. subsp. **_githago_**—NAT

Distribution:—hill, submontane and montane belt.

E, # ***Arenaria bertolonii*** Fiori—LC
Distribution:—common in scree slopes.

\# ***Arenaria grandiflora*** L. subsp. ***grandiflora***
Distribution:—from montane to alpine belt.

\# ***Arenaria leptoclados*** (Rchb.) Guss. subsp. ***leptoclados***
Distribution:—hill, submontane and montane belt.

Arenaria serpyllifolia L. subsp. ***serpyllifolia***
Distribution:—hill, submontane and montane belt.

\# ***Atocion armeria*** (L.) Raf.
Distribution:—from montane to subalpine belt.

\# ***Cerastium arvense*** L. subsp. ***arvense***
Distribution:—hill, submontane and montane belt.

Cerastium arvense L. subsp. ***strictum*** (W.D.J.Koch) Gremli
Distribution:—montane belt.

\# ***Cerastium arvense*** L. subsp. ***suffruticosum*** (L.) Ces.
Distribution:—from hill to alpine belt.

\# ****Cerastium arvense*** subsp. ***suffruticosum*** (L.) Ces. × ***C. tomentosum*** L.
Distribution:—common in the grasslands of the Park as well as in other massifs of the central Apennines. In some cases, it is more widespread than its parent

Cerastium brachypetalum Desp. ex Pers. subsp. ***brachypetalum***
Distribution:—hill, submontane and montane belt.

Cerastium brachypetalum Desp. ex Pers. subsp. ***roeseri*** (Boiss. & Heldr.) Nyman
Distribution:—hill, submontane and montane belt.

Cerastium glomeratum Thuill.
Distribution:—recorded in areas outside the park but near its boundaries: Lucoli (De Santis & Soldati 2019), Prati del Lago! (APP 72888).

Cerastium glutinosum Fr.
Distribution:—montane belt.

Cerastium holosteoides Fr.
Distribution:—montane belt.

Cerastium ligusticum Viv.
Distribution:—hill, submontane and montane belt.

\# ***Cerastium pumilum*** Curtis
Distribution:—montane belt.

E ***Cerastium scaranoi*** Ten.—LC
Distribution:—Mt. Leone (Bertoloni 1833-54), Mt. Ocre (Guarrera & Tammaro 1996).

Cerastium semidecandrum L.
Distribution:—hill, submontane and montane belt.

E ***Cerastium thomasii*** Ten.—LC
Distribution:—screes of Mt. Sirente (Guarrera & Tammaro 1996).

E, # ***Cerastium tomentosum*** L.—LC
Distribution:—common in pastures, often replaced by the hybrid with *C. arvense* subsp. *suffruticosum*

Cherleria capillacea (All.) A.J.Moore & Dillenb. (*Minuartia capillacea* (All.) Graebn.)

Distribution:—from montane to alpine belt.

Dianthus armeria L. subsp. **armeria**

Distribution:—S. Maria in Valle Porclaneta (Vannicelli Casoni et al. 1998), Campo di Rovere! (APP 28660).

Dianthus barbatus L. subsp. **compactus** (Kit.) Heuff.

Distribution:—montane belt.

E **Dianthus carthusianorum** L. subsp. **tenorei** (Lacaita) Pignatti—LC

Distribution:—common in mountain and subalpine pastures.

Dianthus ciliatus Guss. subsp. **ciliatus**

Distribution:—hill and submontane belt.

Dianthus deltoides L. subsp. **deltoides**

Distribution:—montane to alpine belt.

E ***Dianthus guliae** Janka—EN

Distribution:—Mt. Offermo! (APP 59824, 59832, 59833), close to Lake of Bominaco!, Cima di Mallevona!, Colle della Forchetta!, Carrito! (APP 61817, 61818, 61819, 73402; pers. obs.).

Dianthus hyssopifolius L. (*D. marsicus* Ten.; *D. monspessulanus* L.)

Distribution:—montane to subalpine belt.

Dianthus virgineus L. (*D. sylvestris* Wulfen subsp. *longicaulis* (Ten.) Greuter & Burdet; *D. longicaulis* Ten.; *D. sylvestris* auct. Fl. Ital. p.p.)

Distribution:—from hill to alpine belt.

Dichodon cerastoides (L.) Rchb. (*Cerastium cerastoides* (L.) Britton)

Distribution:—Velino! (Martelli 1904; Grande 1912; Montelucci 1958).

Dichodon viscidum (M.Bieb.) Holub (*Cerastium dubium* (Bastard) Guépin)

Distribution:—Prati del Lago! (Conti et al. 2025a; APP 73008). Found near the park boundaries.

Drypis spinosa L. subsp. **spinosa**

Distribution:—from hill to alpine belt.

Heliosperma pusillum (Waldst. & Kit.) Rchb. subsp. **pusillum** (*Silene pusilla* Waldst. & Kit.; *Silene quadridentata* (L.) Pers. subsp. *pusilla* (Waldst. & Kit.) H.Neumayer)

Distribution:—from montane to alpine belt.

E **Herniaria bornmuelleri** Chaudhri—LC

Distribution:—Velino, Mt. Sirente (Chaudri 1968).

Herniaria glabra L. subsp. **glabra**

Distribution:—montane belt.

Herniaria glabra L. subsp. **nebrodensis** Nyman

Distribution:—from montane to alpine belt.

Herniaria hirsuta L. subsp. **hirsuta**

Distribution:—from montane to subalpine belt.

Herniaria incana Lam.

Distribution:—hill, submontane and montane belt.

***Holosteum umbellatum** L.

Distribution:—Forme! (APP 73364), Terranera! (pers. obs.).

Lychnis coronaria (L.) Desr.

Distribution:—montane belt.

Lychnis flos-cuculi L. subsp. ***flos-cuculi***

Distribution:—montane belt.

E, # ***Mcneillia rosanoi*** (Ten.) F.Conti & Del Guacchio subsp. ***rosanoi*** (*Minuartia graminifolia* (Ard.) Jáv. subsp. *rosanoi* (Ten.) Mattf.; *Mcneillia graminifolia* (Ard.) Dillenb. & Kadereit subsp. *rosanoi* (Ten.) F.Conti, Bartolucci, Iamonico & Del Guacchio)—LC

Distribution:—Velino! (Montelucci 1958 as *Alsine graminifolia*; Petriccione 1993 as *Minuartia graminifolia*; Conti 2003), Gole di Celano!, Prati del Sirente (Frizzi et al. 1996; Guarrera & Tammaro 1996 as *Minuaria graminifolia*; Conti 2003; APP 42428, 68186), Mt. Canale (Conti 2003 from a specimen collected by Groves), Mt. Briccialone!, Serra di Celano! (APP 67200, 67646).

E ***Minuartia glomerata*** (M.Bieb.) Degen subsp. ***trichocalycina*** (Ten. & Guss.) F.Conti—LC

Distribution:—Prati del Sirente, Ocre! (Tammaro et al. 1974; Guarrera & Tammaro 1996, Conti 1997), above Forme (Lucchese & Lattanzi 1993), Mt. Canale, Collarmele, Celano!, Serra di Celano!, Bominaco!, Prati del Lago! (Conti 1997 from specimens collected by Groves and Calestani; APP 36045, 67653, 67856, 67857).

Moehringia muscosa L.

Distribution:—V.ne di Teve!, Velino (Lucchese & Lattanzi 1993; Petriccione 1993; pers. obs.).

Moehringia trinervia (L.) Clairv.

Distribution:—montane belt.

Paronychia kapela (Hacq.) A.Kern. subsp. ***kapela***

Distribution:—from the hill to alpine belt.

Petrorhagia prolifera (L.) P.W.Ball & Heywood

Distribution:—hill, submontane and montane belt.

Petrorhagia saxifraga (L.) Link subsp. ***saxifraga***

Distribution:—hill, submontane and montane belt.

****Polycarpon tetraphyllum*** (L.) L. subsp. ***tetraphyllum***

Distribution:—Rocca di Mezzo!, Santa Maria del Ponte!, Gagliano Aterno!, Acciano! (APP 73436, 74183, 74215).

Rabelera holostea (L.) M.T.Sharples & E.A.Tripp (*Stellaria holostea* L.)

Distribution:—hill, submontane and montane belt.

Sabulina attica (Boiss. & Sprun.) Dillenb. & Kadereit subsp. ***attica***

Distribution:—from hill to alpine belt.

Sabulina glaucina (Dvořáková) Dillenb. & Kadereit

Distribution:—from hill to alpine belt.

Sabulina tenuifolia (L.) Rchb. subsp. ***tenuifolia***

Distribution:—hill, submontane and montane belt.

Sabulina verna (L.) Rchb. subsp. ***verna***

Distribution:—subalpine and alpine belt.

Sagina alexandrae Iamonico (*S. subulata* (Sw.) C.Presl, *nom. illeg.*)
Distribution:—Prati del Sirente (Guarrera & Tammaro 1996).
Sagina apetala** Ard. subsp. **apetala
Distribution:—Ovindoli!, Rocca di Mezzo! (APP 73439, 74211).
Sagina glabra (Willd.) Fenzl
Distribution:—from montane to alpine belt.
Sagina procumbens** L. subsp. **procumbens
Distribution:—Rosciolo! (pers. obs.).
Saponaria bellidifolia Sm.
Distribution:—montane and sbalpine belt.
Saponaria ocymoides L. subsp. ***ocymoides***
Distribution:—hill, submontane and montane belt.
Saponaria officinalis L.
Distribution:—hill, submontane and montane belt.
Scleranthus annuus L.
Distribution:—montane belt.
Scleranthus polycarpos L.
Distribution:—montane belt.
Scleranthus verticillatus Tausch
Distribution:—montane belt.
Silene acaulis (L.) Jacq. subsp. ***bryoides*** (Jord.) Nyman
Distribution:—alpine belt.
Silene baccifera (L.) Durande (*Cucubalus baccifer* L.)
Distribution:—Mt. Sirente (Groves 1880; Guarrera & Tammaro 1996), Lago Acquaviva near Molina! (APP 27198, 27202).
E ***Silene cattariniana*** Ferrarini & Cecchi —LC
Distribution:—Mt. Sirente (Groves 1880 as *S. vallesia*; Guarrera & Tammaro 1996 as *S. vallesia* subsp. *graminea*; Ferrarini & Cecchi 2001).
Silene ciliata Pourr. subsp. ***graefferi*** (Guss.) Nyman
Distribution:—from montane to alpine belt.
Silene conica L.
Distribution:—hill, submontane and montane belt.
Silene cretica L.—NC
Distribution:—Rupi del Rio below Castelvecchio Subequo (Groves 1880).
Silene dioica (L.) Clairv.
Distribution:—montane belt.
Silene italica (L.) Pers. subsp. ***italica***
Distribution:—hill, submontane and montane belt.
Silene latifolia Poir.
Distribution:—hill, submontane and montane belt.
Silene multicaulis Guss. subsp. ***multicaulis***
Distribution:—from the hill to alpine belt.
Silene muscipula L. subsp. *muscipula*—NC
Distribution:—Velino (Martelli 1904).
Silene nemoralis Waldst. & Kit.

Distribution:—hill, submontane and montane belt.

E, # ***Silene notarisii*** Ces.—DD
Distribution:—Velino (Montelucci 1958 as *S. saxifraga* var. *parnassica*; Petriccione 1993 as *S. parnassica*).

Silene nutans L. subsp. ***insubrica*** (Gaudin) Soldano
Distribution:—Mt. Sirente (Jeanmonod & Bocquet 1983).

\# ***Silene nutans*** L. subsp. ***nutans***
Distribution:—montane belt.

\# ***Silene otites*** (L.) Wibel subsp. ***otites***
Distribution:—hill, submontane and montane belt.

Silene paradoxa L.
Distribution:—hill, submontane and montane belt.

E ***Silene roemeri*** Friv. subsp. ***staminea*** (Bertol.) Nyman (*S. staminea* Bertol.)—DD
Distribution:—Mt. Sirente (Groves 1880; Veri & Tammaro 1980; Foggi & Ricceri 1993 from specimens collected by Groves and Profeta), Mt. Bicchero (Lucchese & Lattanzi 1993), Campo Felice (De Santis & Soldati 2011), Cimata di Pezza! (APP 67920).

Silene saxifraga L.
Distribution:—Sirente (Veri & Tammaro 1980), Gole di Celano! (Frizzi et al. 1996; Guarrera & Tammaro 1996; APP 9451, 9452, 9453, 9454, 9455, 9456, 68187, 68188), M. Briccialone!, Serra di Celano!, below "La Neviera"! (APP 67176, 67652, 73388).

Silene viridiflora L.
Distribution:—montane and subalpine belt.

\# ***Silene vulgaris*** (Moench) Garcke subsp. ***prostrata*** (Gaudin) Schinz & Thell.
Distribution:—montane to subalpine belt.

****Silene vulgaris*** (Moench) Garcke subsp. ***tenoreana*** (Colla) Soldano & F.Conti
Distribution:—Castelvecchio Subequo! (APP 73491).

\# ***Silene vulgaris*** (Moench) Garcke subsp. ***vulgaris***
Distribution:—hill, submontane and montane belt.

Spergularia rubra (L.) J.Presl & C.Presl
Distribution:—reported for the neighbouring territory of Lucoli (De Santis & Soldati 2019) and likely also present in the Park.

****Stellaria aquatica*** (L.) Scop.
Distribution:—Tione!, Molina Aterno! (APP 28202, 28381).

Stellaria graminea L.
Distribution:—Val d'Arano! (Conti 1998; APP 28603), Altopiano delle Rocche! (APP 59619, 67837).

Stellaria media (L.) Vill.
Distribution:—hill, submontane and montane belt.

\# ***Stellaria neglecta*** Weihe subsp. ***neglecta***
Distribution:—Velino (Petriccione 1993).

\# ***Stellaria nemorum*** L. subsp. ***montana*** (Pierrat) Berher
Distribution:—montane belt.

Stellaria pallida (Dumort.) Crép.
Distribution:—Gole di Celano (Frizzi et al. 1996; Guarrera & Tammaro 1996).

CELASTRACEAE

Euonymus europaeus L.
Distribution:—hill, submontane and montane belt.

Euonymus latifolius (L.) Mill. subsp. ***latifolius***
Distribution:—hill, submontane and montane belt.

Euonymus verrucosus Scop.
Distribution:—hill, submontane and montane belt.

CERATOPHYLLACEAE

****Ceratophyllum demersum*** L.
Distribution:—Lago di Tempra! (APP 73463).
****Ceratophyllum submersum*** L. subsp. ***submersum***
Distribution:—Lago di Bominaco! (pers. obs.).

CISTACEAE

Cistus creticus L. subsp. ***creticus***
Distribution:—Gole di S. Venanzio, Vittorito (Pirone & Tammaro 1997; Pirone & Cutini 2002; Corbetta et al. 2004).
Cistus creticus L. subsp. ***eriocephalus*** (Viv.) Greuter & Burdet
Distribution:—hill and submontane belt.
Fumana ericifolia Wallr.
Distribution:—Vittorito (Corbetta et al. 2004), Bocca di Teve! (APP 68383).

Fumana procumbens (Dunal) Gren. & Godr.
Distribution:—hill and submontane belt.
Fumana thymifolia (L.) Spach ex Webb
Distribution:—hill and submontane belt.

Helianthemum apenninum (L.) Mill. subsp. ***apenninum***
Distribution:—hill, submontane and montane belt.

Helianthemum nummularium (L.) Mill. subsp. ***glabrum*** (W.D.J.Koch)
Distribution:—montane belt.
Helianthemum nummularium (L.) Mill. subsp. ***grandiflorum*** (Scop.) Schinz & Thell.
Distribution:—from montane to alpine belt.
Helianthemum nummularium (L.) Mill. subsp. ***nummularium***
Distribution:—hill and submontane belt.

Helianthemum nummularium (L.) Mill. subsp. ***obscurum*** (Čelak.) Holub
Distribution:—hill, submontane and montane belt.

Helianthemum oelandicum (L.) Dum.Cours. subsp. ***alpestre*** (Jacq.) Ces.
Distribution:—from montane to alpine belt.

Helianthemum oelandicum (L.) Dum.Cours. subsp. ***incanum*** (Willk.) G.López
Distribution:—from hill, submontane to alpine belt.

Helianthemum oelandicum (L.) Dum.Cours. subsp. ***italicum*** (L.) Ces.

Distribution:—Velino (Tenore 1830; Martelli 1904; Montelucci 1958; Petriccione 1993), Gole di Celano (Frizzi et al. 1996; Guarrera & Tammaro 1996),

Helianthemum salicifolium (L.) Mill.

Distribution:—hill and submontane belt.

COLCHICACEAE

Colchicum alpinum DC.

Distribution:—montane belt.

Colchicum bulbocodium Ker Gawl. subsp. ***versicolor*** (Ker Gawl.) K.Perss.

Distribution:—Piana di Campo Felice! (Conti et al. 2008a; APP 30130), Piani di Pezza! (APP 50265, 67019).

Colchicum lusitanum Brot.—LC

Distribution:—montane belt.

E, # ***Colchicum neapolitanum*** (Ten.) Ten. subsp. ***neapolitanum*** —LC

Distribution:—Mt. Sirente, Velino (Groves 1880, Martelli 1904; Grande 1924 da Groves e Martelli), V.ne di Teve (Lucchese & Lattanzi 1993), Campo Felice (De Santis & Soldati 2011).

CONVOLVULACEAE

Convolvulus arvensis L.

Distribution:—hill, submontane and montane belt.

Convolvulus cantabrica L.

Distribution:—hill, submontane and montane belt.

Convolvulus elegantissimus Mill.

Distribution:—Vittorito (Corbetta et al. 2004), Gole di San Venanzio!, Castelvecchio Subequo!, Pié della Selva a Fontecchio!, Macchione a Goriano Sicoli!, S. Rocco near Fagnano Alto! (APP 27046, 28304, 28898, 73488, 74282).

Convolvulus sepium L.

Distribution:—hill, submontane and montane belt.

N ***Cuscuta campestris*** Yunck.—NAT

Distribution:—Velino (Martelli 1904; Viegi et al. 1990).

****Cuscuta epithymum*** (L.) L. subsp. ***kotschyi*** (Des Moul.) Arcang.

Distribution:—the previous reports of *C. epithymum* for Velino (Steinberg 1953; Montelucci 1958; Petriccione 1993) and Val d'Arano (Guarrera & Tammaro 1996) should probably to be referred to this taxon.

Cuscuta europaea L.

Distribution:—hill, submontane and montane belt.

C *Cuscuta monogyna* Vahl subsp. *monogyna* —NC, DD

Distribution:—Velino (Martelli 1904).

Cuscuta planiflora Ten.

Distribution:—montane belt.

N ***Ipomoea purpurea*** (L.) Roth—CAS

Distribution:—Castelvecchio Subequo (Viegi et al. 1990), Acciano! (APP 74177, 74179).

CORNACEAE

Cornus mas L.
Distribution:—hill, submontane and montane belt.

Cornus sanguinea L. subsp. **hungarica** (Kárpáti) Soó
Distribution:—hill, submontane and montane belt.

CRASSULACEAE

***Hylotelephium maximum** (L.) Holub subsp. **maximum**
Distribution:—near Campana! (pers. obs.).

Petrosedum rupestre (L.) P.V.Heath (*Sedum rupestre* L.)
Distribution:—hill, submontane to subalpine belt.

Petrosedum sediforme (Jacq.) Grulich subsp. *sediforme*—D
Distribution:—Mt. Sirente (Tammaro et al. 1974; Veri & Tammaro 1980; Guarrera & Tammaro 1993).

Petrosedum tenuifolium (Sm.) Grulich—NC
Distribution:—Mt. Sirente (Groves 1880 as *Sedum tenuifolium*; Guarrera & Tammaro 1996 as *Sedum amplexicaule* subsp. *tenuifolium*), Velino (Blasi et al. 1992 as *Sedum pruinatum* subsp. *tenuifolium*). At least the most recent reports are doubtful; the species is to be confirmed.

Phedimus stellatus (L.) Raf.—NC
Distribution:—lower slopes of Mt. Velino (Brocchi 1823).

Sedum acre L.
Distribution:—hill, submontane to montane belt.

Sedum album L.
Distribution:—hill, submontane to montane belt.
Note:—there are two subspecies in Italy: *S. album* subsp. *album* and *S. album* subsp. *micranthum* (Bast. ex DC.) Syme, but the differences in our samples are not clear.

E **Sedum aquilanum** L.Gallo & F.Conti—CR
Distribution:—Campo Felice! (Minutillo et al. 2010 as *S. nevadense*; Gallo & Conti 2015; APP 38348, 38349, 40593, 67736). Endemic to Campo Felice.

Sedum atratum L.
Distribution:—subalpine to alpine belt.

***Sedum caespitosum** (Cav.) DC.
Distribution:—Forme! (pers. obs.), Terranera! (APP 73447).

Sedum cepaea L.
Distribution:—reported for the neighbouring territory of Lucoli (De Santis & Soldati 2019) and likely also present in the Park.

Sedum dasyphyllum L.
Distribution:—hill, submontane to montane belt.
Note:—there are two subspecies in Italy: *S. dasyphyllum* subsp. *dasyphyllum* and *S. dasyphyllum* subsp. *glanduliferum* (Guss.) Nyman but the differences in our samples are not clear.

Sedum hispanicum L.
Distribution:—hill, submontane to montane belt.

Sedum magellense Ten. subsp. ***magellense***—LC
Distribution:—from montane to alpine belt.
****Sedum rubens*** L.
Distribution:—Forme!, Colle del Rascito! (pers. obs.).
Sedum sexangulare L.
Distribution:—hill, submontane and montane belt.
E ***Sempervivum ×luisae*** L.Gallo
Distribution:—Colle di Ocre (Gallo 2012).
Sempervivum arachnoideum L.
Distribution:—from montane to alpine belt.
E, # ***Sempervivum riccii*** Iberite & Anzal.—LC
Distribution:—Velino (Montelucci 1958 as *S. tectorum* var. *clusianum*; Ricci 1961 as *S. italicum*; Petriccione 1993 as *S. italicum*; Iberite & Anzalone 2001 from a specimen collected by Ricci; Gallo 2012), Valle Lupara (Guarrera & Tammaro 1996 as *S. italicum*), Mt. Offermo! (Gallo 2012; APP 26429), near Terranera!, close to Carrito! (APP 33301, 66811).
Sempervivum tectorum L.
Distribution:—hill, submontane to alpine belt.
Umbilicus horizontalis (Guss.) DC.
Distribution:—hill, submontane to montane belt.

CUCURBITACEAE
Bryonia dioica Jacq.
Distribution:—hill and submontane belt.
****Ecballium elaterium*** (L.) A.Rich.
Distribution:—Secinaro!, Acciano! (pers. obs.).

CYPERACEAE
Blysmus compressus (L.) Panz. ex Link
Distribution:—montane belt.
Carex acuta L.
Distribution:—montane belt.
Carex acutiformis Ehrh.
Distribution:—Molina Aterno! (Conti 1993; APP 28487, 28488, 28489).
Carex basilaris Jord.—D
Distribution:—Vallone il Bicchero (Avena & Blasi 1975 as *C. depressa*), Velino (Pignatti 1982 as *C. depressa*). The report by Avena & Blasi (1975), cited by Pignatti, is questionable and needs to be confirmed.
Carex caryophyllea Latourr.
Distribution:—hill, submontane and montane belt.
Carex davalliana Sm.
Distribution:—Altopiano delle Rocche (Ballelli & Pedrotti 1979), Settacque (De Santis & Soldati 2019), Campo di Rovere! (Ciaschetti et al. 2024; APP 9379, 28649, 34709, 64978), Conche di Ovindoli! (APP 66487, 66785).
Carex digitata L.

Distribution:—montane belt.

Carex distachya Desf. subsp. ***distachya***

Distribution:—Mt. Sirente (Groves 1880), eremo di Madonna di Pietrabona near Castel di Ieri! (APP 72878).

Carex distans L.

Distribution:—montane belt.

Carex divulsa Stokes

Distribution:—montane belt.

Carex echinata Murray subsp. ***echinata***

Distribution:—montane belt.

****Carex elata*** All. subsp. ***elata***

Distribution:—Conche di Ovindoli!, Colle Ciaccio! (APP 66495, 66524, 72900).

Carex flacca Schreb. subsp. ***erythrostachys*** (Hoppe) Holub

Distribution:—hill, submontane and montane belt.

Carex flacca Schreb. subsp. ***flacca***

Distribution:—hill, submontane and montane belt.

Carex halleriana Asso

Distribution:—hill, submontane and montane belt.

Carex hartmaniorum A.Cajander

Distribution:—Piana di Ovindoli!, Val d'Arano! (Bartolucci et al. 2025; APP 68475, 73375, 73377, 73378, 73379, 73380, 73548, 73549). Previous reports of *C. buxbaumii* for Abruzzo (Bartolucci et al. 2025) should be referred to this species.

Carex hirta L.

Distribution:—hill, submontane and montane belt.

Carex hostiana DC.

Distribution:—Campo di Rovere! (Conti & Bartolucci 2011; Ciaschetti et al. 2024; APP 9380).

Carex humilis Leyss.

Distribution:—hill, submontane and montane belt.

Carex kitaibeliana Degen ex Bech.

Distribution:—montane to alpine belt.

Carex leporina L.

Distribution:—montane belt.

Carex liparocarpos Gaudin subsp. ***liparocarpos***

Distribution:—hill and submontane belt.

Carex macrolepis DC.—LC

Distribution:—montane to subalpine belt.

Carex mucronata All.—NC

Distribution:—Velino, Mt. Sirente (Furrer 1928).

Carex muricata L. subsp. ***muricata***—LC

Distribution:—montane belt.

Carex nigra (L.) Reichard subsp. ***nigra***

Distribution:—montane belt.

Carex oederi Retz.

Distribution:—montane belt.

*Carex otrubae*** Podp.
Distribution:—hill, submontane and montane belt.

Carex pairae F.W.Schultz
Distribution:—montane belt.

****Carex pallescens*** L.
Distribution:—Piana di Ovindoli! (APP 73429).

Carex panicea L.
Distribution:—montane belt.

Carex pendula Huds.
Distribution:—hill, submontane and montane belt.

Carex pilosa Scop.
Distribution:—montane belt.

****Carex praecox*** Schreb.
Distribution:—Piano di S. Nicola!, Piano S. di Rufino! (APP 62168, 62181).

Carex riparia Curtis
Distribution:—Molina Aterno! (Tammaro 1988; Conti 1993; APP 27192, 27193, 28560, 28561, 28562, 28563, 28564, 28565, 28566, 73036).

Carex spicata Huds. subsp. ***spicata***
Distribution:—montane belt.

Carex sylvatica Huds.
Distribution:—montane belt.

****Carex tomentosa*** L.
Distribution:—Campo Felice! (APP 67743).

Carex umbrosa Host subsp. *umbrosa*—NC
Distribution:—Velino (Petriccione 1993).

Carex vesicaria L.
Distribution:—Val d'Arano (Ciaschetti et al. 2005)

Carex vulpina L.—EN
Distribution:—Val d'Arano (Costalonga 2004), Laghetto nella Piana di Ovindoli!, Conche di Ovindoli!, Prato della Corte (Terranera)!, Fosso Scettro!, Prati della Madonna!, Colle Ciaccio! (APP 9482, 9485, 9486, 9487, 35353, 66486, 67059, 68115, 68116, 72899).

Cyperus longus L.
Distribution:—hill, submontane and montane belt.

Eleocharis palustris (L.) Roem. & Schult. subsp. ***palustris***
Distribution:—some specimens collected at Conche di Ovindoli, Campo di Rovere, and Prati del Lago near S. Panfilo d'Ocre (34803, 66537, 68020, 68071, 68395), in the absence of plants with mature capsules, have been tentatively attributed by us to *E. palustris* (L.) Roem. & Schult. subsp. *waltersii* Bureš & Danihelka.

Eleocharis quinqueflora (Hartmann) O.Schwarz
Distribution:—Campo Felice!, Campo di Rovere!, Piani di Pezza! (Conti & Manzi 1998; Conti et al. 2008a; Ciaschetti et al. 2024; APP 30156, 30167, 34707, 66695).

Eleocharis uniglumis (Link) Schult.
Distribution:—Campo Felice! (De Santis & Soldati 2011; APP 28192, 30140, 30166), Campo di Rovere!, Conche di Ovindoli! (APP 28667, 34804, 66493, 68308).

Eriophorum latifolium Hoppe
Distribution:—between Rocca di Mezzo and Rocca di Cambio! (Guarrera & Tammaro 1996), Conche di Ovindoli! (APP 66489)

Isolepis cernua (Vahl) Roem. & Schult.—NC
Distribution:—Mt. Sirente along F.Aterno (Groves 1880).

Schoenoplectus lacustris (L.) Palla
Distribution:—hill, submontane and montane belt.

Scirpoides holoschoenus (L.) Soják
Distribution:—hill, submontane and montane belt.

DIOSCOREACEAE

\# ***Dioscorea communis*** (L.) Caddick & Wilkin
Distribution:—hill, submontane and montane belt.

DIPSACACEAE

\# ***Cephalaria leucantha*** (L.) Roem. & Schult.
Distribution:—hill, submontane and montane belt.

Cephalaria transsylvanica (L.) Roem. & Schult.
Distribution:—hill and submontane belt.

\# ***Dipsacus fullonum*** L. subsp. ***fullonum***
Distribution:—hill, submontane and montane belt.

Knautia arvensis (L.) Coult.
Distribution:—montane belt.

E ***Knautia calycina*** (C.Presl) Guss.—LC
Distribution:—common in mountain pastures.

Knautia integrifolia (L.) Bertol. subsp. ***integrifolia***
Distribution:—hill, submontane and montane belt.

Lomelosia argentea (L.) Greuter & Burdet
Distribution:—hill and submontane belt.

\# ***Lomelosia graminifolia*** (L.) Greuter & Burdet subsp. ***graminifolia***
Distribution:—montane to alpine belt.

\# ***Scabiosa columbaria*** L.
Note:—the Apennine populations are of uncertain attribution and should probably belong to the subsp. *portae* (A.Kern. ex Huter) Hayek.

Scabiosa pyrenaica All.
Distribution:—subalpine and alpine belt.

\# ***Scabiosa triandra*** L.
Distribution:—hill and submontane belt.

E ***Scabiosa uniseta*** Savi—LC
Distribution:—Gole di Celano, Prati del Sirente, between Castelvecchio Subequo and Collarmele (Frizzi et al. 1996; Guarrera & Tammaro 1996).

Sixalix atropurpurea (L.) Greuter & Burdet
Distribution:—hill and submontane belt.
Succisa pratensis Moench
Distribution:—Val d’Arano! (Tammaro 1984), near Ocre below the castle (Guarrera et al. 1996), Campo di Rovere! (Ciaschetti et al. 2024; APP 28674, 57251, 68021, 68022, 68023), Rocca di Cambio! (APP 11269, 11270, 59605, 59606), Conche di Ovindoli! (APP 66527, 66777, 66778, 66784).

ERICACEAE
Arbutus unedo L.
Distribution:—Gole di S. Venanzio! (D’Errico 1936; Montelucci 1971; Pirone 1995), (APP 28505).
Arctostaphylos uva-ursi (L.) Spreng.
Distribution:—montane and subalpine belt.
Monotropa hypophegea Wallr.
Distribution:—Piani di Pezza! (Conti et al. 2008a; APP 66743), beech forest of Sacco Sirente! (APP 27732).
Monotropa hypopitys L.
Distribution:—Velino (Petriccione 1993, 2005), Mt. delle Canelle! (pers. obs.).
Orthilia secunda (L.) House
Distribution:—montane belt.
Pyrola minor L.
Distribution:—beech forest of Mt. Sirente (Guarrera & Tammaro 1996), Costa della Tavola! (APP 67625).

EUPHORBIACEAE
Chrozophora tinctoria (L.) A.Juss.
Distribution:—hill and submontane belt.
Euphorbia amygdaloides L.
Distribution:—hill, submontane and montane belt.
Euphorbia chamaesyce L.
Distribution:—hill, submontane and montane belt.
Euphorbia characias L.
Distribution:—between Forme and Massa d'Albe (Lucchese & Lattanzi 1993).
Euphorbia cyparissias L.
Distribution:—from hill to subalpine belt.
Euphorbia dulcis L.
Distribution:—montane belt.
Euphorbia falcata L.
Distribution:—hill, submontane and montane belt.
E ***Euphorbia gasparrinii*** Boiss. subsp. ***samnitica*** (Fiori) Pignatti—LC
Distribution:—common in mountain pastures, especially on high plateaus.
Euphorbia helioscopia L. subsp. ***helioscopia***
Distribution:—hill, submontane and montane belt.

Euphorbia japygica Ten. subsp. ***prostrata*** (Fiori) Del Guacchio & Frajman *(Euphorbia nicaeensis* auct. Fl. Ital.)
Distribution:—hill, submontane and montane belt.

N ****Euphorbia maculata*** L.—NAT
Distribution:—staz. di Fagnano-Campana! (APP 74235).

Euphorbia myrsinites L. subsp. ***myrsinites***
Distribution:—hill, submontane and montane belt.

Euphorbia peplus L.
Distribution:—ill and submontane belt.

****Euphorbia platyphyllos*** L.
Distribution:—Castelnuovo! (pers. obs.)

N ****Euphorbia saratoi*** Ardoino—NAT
Distribution:—Prati del Lago (Ocre)! (Conti et al. 2008a as *E. esula* L. subsp. *tommasiniana* (Bertol.) Kuzsamanov; APP 25284). The occurence of *E. tommasiniana* Bertol. should be excluded from the flora of Abruzzo.

Euphorbia spinosa L. subsp. ***spinosa***
Distribution:—hill and submontane belt.

Mercurialis annua L.
Distribution:—hill, submontane and montane belt.

Mercurialis ovata Sternb. & Hoppe
Distribution:—montane and subalpine belt

Mercurialis perennis L.
Distribution:—montane belt.

FABACEAE

E, # ***Anthyllis apennina*** F.Conti & Bartolucci—LC
Distribution:—Prati del Sirente!, Piani di Pezza!, Mt. Ocre!, V.ne di Sevice!, Ovindoli!, Piano di Canale! (Conti & Bartolucci 2021; APP 9343, 55537, 55538, 55539, 55540, 55541, 55542, 55543, 55544, 59702, 59703, 59704, 59705, 59706, 59707, 59708, 59709, 59710, 59711, 59801, 59802, 64972, 64973, 64974, 64979, 64980, 72856, 73451).

Anthyllis montana L. subsp. ***jacquinii*** (Rchb.f.) Rohlena
Distribution:—montane belt.

****Anthyllis vulneraria*** L. subsp. ***carpatica*** (Pant.) Nyman
Distribution:—Piani di Pezza!, between Rocca di Mezzo and Piani di Pezza!, Piana di Ovindoli at the base of the ski lifts!, Conche di Ovindoli! (APP 9504, 9505, 60347, 60349, 60352, 64904, 64905, 64906, 64907, 64908, 64909, 64910, 64911, 64912, 64913, 64914, 64915, 68239, 73432, 73433).

Anthyllis vulneraria L. subsp. ***maura*** (Beck) Maire
Distribution:—hill and submontane belt.

E, # ***Anthyllis vulneraria*** L. subsp. ***nana*** (Ten.) Tammaro—LC
Distribution:—widespread in high mountain pastures.
Note:—the boundaries with respect to the subsp. *pulchella* are unclear and require further investigation.

Anthyllis vulneraria L. subsp. ***polyphylla*** (DC.) Nyman

Distribution:—montane and subalpine belt.

Anthyllis vulneraria L. subsp. *pulchella* (Vis.) Bornm.—D

Note:—the previous reports probably belong to *A. apennina* or *A. vulneraria* subsp. *nana* .

Anthyllis vulneraria L. subsp. ***rubriflora*** (DC.) Arcang.

Distribution:—hill, submontane and montane belt.

Argyrolobium zanonii (Turra) P.W.Ball subsp. ***zanonii***

Distribution:—hill and submontane belt.

E ***Astragalus aquilanus*** Anzal.—EN

Distribution:—near Terranera (Frattaroli & Frizzi 1988) where it has not been confirmed in recent times, between S. Benedetto in Perillis and Acciano!, Goriano Sicoli on the edge of the oak forest!, Mt. Le Serre (Goriano Sicoli)!, Secina in the municipality of Prezza! (APP 73564; pers. obs.).

Astragalus danicus Retz.

Distribution:—montane belt.

Astragalus depressus L. subsp. ***depressus***

Distribution:—montane to alpine belt.

Astragalus glycyphyllos L.

Distribution:—montane belt.

Astragalus hamosus L.

Distribution:—hill and submontane belt.

Astragalus monspessulanus L. subsp. ***monspessulanus***

Distribution:—hill and submontane belt.

Astragalus sempervirens Lam.

Distribution:—montane and subalpine belt.

Astragalus sesameus L.

Distribution:—hill and submontane belt.

E, # *Astragalus sirinicus* Ten.—NC, LC

Distribution:—Mt. Sirente (Groves 1880).

Astragalus vesicarius L. subsp. ***vesicarius***

Distribution:—montane belt.

Bituminaria bituminosa (L.) C.H.Stirt. (*Psoralea bituminosa* L.)

Distribution:—hill and submontane belt.

Cercis siliquastrum L. subsp. ***siliquastrum***

Distribution:—Gole di S. Venanzio (Viegi et al. 1990).

Colutea arborescens L.

Distribution:—hill, submontane and montane belt.

Coronilla minima L. subsp. ***minima***

Distribution:—from hill to alpine belt.

Coronilla scorpioides (L.) W.D.J.Koch

Distribution:—hill and submontane belt.

Coronilla vaginalis Lam.

Distribution:—montane and subalpine belt.

Cytisophyllum sessilifolium (L.) O.Lang

Distribution:—hill, submontane and montane belt.

Cytisus decumbens (Durande) Spach
Distribution:—hill, submontane and montane belt.
Cytisus scoparius (L.) Link subsp. ***scoparius***
Distribution:—Piani di Pezza (Lucchese & Lattanzi 1993).
Cytisus spinescens Sieber ex Spreng.
Distribution:—hill and submontane belt.
Cytisus triflorus Lam. (*C. hirsutus* auct. fl. Ital.)
Note:—previous reports of *C. hirsutus* in the central-southern Apennines refer to this taxon.
Cytisus villosus Pourr.
Distribution:—hill and submontane belt.
Emerus major Mill.
Note:—the subsp. *major* and subsp. *emeroides* (Boiss. & Spruner) Soldano & F.Conti are indicated in the Park, but the differences between the two taxa are unclear. The species deserves further investigation.
Ervilia hirsuta (L.) Opiz (*Vicia hirsuta* (L.) Gray)
Distribution:—montane belt.
****Ervilia loiseleurii*** (M.Bieb.) H.Schaef., Coulot & Rabaute (*Vicia loiseleurii* (M.Bieb.) Litv.)
Distribution:—Cerreta di Goriano Sicoli! (APP 73005, 73566).
A ***Ervilia sativa*** Link (*Vicia ervilia* (L.) Willd.)—CAS
Distribution:—Gole di Celano, Molina Aterno (Viegi et al. 1990).
****Ervum gracile*** DC. (*Vicia parviflora* Cav.; *V. tenuissima* (Bieb.) Sch. *& Th.*)
Distribution:—Forme! (pers. obs.)
A ***Galega officinalis*** L.—NAT
Distribution:—hill and submontane belt.
Genista januensis Viv. subsp. ***januensis***
Distribution:—montane belt.
Genista radiata (L.) Scop.
Distribution:—ridge above Valle Ortica berween Piani di Pezza and Cimata di Pezza! (Conti et al. 2023a; APP 67914, 68389).
Genista sagittalis L.
Distribution:—montane belt.
Genista tinctoria L.
Distribution:—hill, submontane and montane belt.
****Hippocrepis biflora*** Spreng.
Distribution:—Collarmele!, Massa d'Albe!, Rosciolo!, Carrito! (pers. obs.).
Hippocrepis comosa L. subsp. ***comosa***
Distribution:—from hill to alpine belt.
Hippocrepis glauca Ten.
Distribution:—subalpine and alpine belt.
Laburnum anagyroides Medik. subsp. ***anagyroides***
Note:—previous reports of *L. alpinum* (Petriccione 1993) should be referred to this taxon.
Lathyrus annuus L.

Distribution:—Piè di Cafornia (Avena & Blasi 1975)
Lathyrus aphaca L. subsp. ***aphaca***
Distribution:—hill and submontane belt.
Lathyrus cicera L.
Distribution:—hill and submontane belt.
Lathyrus hirsutus L.
Distribution:—between Aielli and Ovindoli, Molina Aterno (Guarrera & Tammaro 1996).
****Lathyrus inconspicuus*** L.
Distribution:—Cerreta di Goriano Sicoli! (APP 73568, 73569).
Lathyrus latifolius L.
Distribution:—hill and submontane belt.
****Lathyrus niger*** (L.) Bernh.
Distribution:—Cerreta di Goriano Sicoli! (APP 73003).
Lathyrus nissolia L.
Distribution:—montane belt.
Lathyrus oleraceus Lam. subsp. ***biflorus*** (Raf.) H.Schaef., Coulot & Rabaute
Distribution:—hill, submontane and montane belt.
Lathyrus pannonicus (Jacq.) Garcke subsp. ***asphodeloides*** (Gouan) Bässler
Distribution:—montane belt.
Lathyrus pratensis L. subsp. ***pratensis***
Distribution:—hill, submontane and montane belt.
Lathyrus setifolius L.
Distribution:—hill, submontane and montane belt.
Lathyrus sphaericus Retz.
Distribution:—hill and submontane belt.
Lathyrus sylvestris L. subsp. ***sylvestris***
Distribution:—hill, submontane and montane belt.
Lathyrus venetus (Mill.) Wohlf.
Distribution:—hill, submontane and montane belt.
Lathyrus vernus (L.) Bernh.
Distribution:—hill, submontane and montane belt.
Lotus corniculatus L. subsp. ***alpinus*** (DC.) Rothm.
Distribution:—subalpine and alpine belt.
Lotus corniculatus L. subsp. ***corniculatus***
Distribution:—hill, submontane and montane belt.
Lotus dorycnium L. subsp. ***dorycnium***
Distribution:—Velino (Petriccione 1993).
Lotus dorycnium L. subsp. ***herbaceus*** (Vill.) Kramina & D.D.Sokoloff
Distribution:—hill, submontane and montane belt.
Lotus hirsutus L.
Distribution:—hill and submontane belt.
Lotus pedunculatus Cav.
Distribution:—montane belt.
Lotus tenuis Waldst. & Kit. ex Willd.

Distribution:—hill, submontane and montane belt.

Medicago arabica (L.) Huds.

Distribution:—hill and submontane belt.

Medicago falcata L. subsp. ***falcata***

Distribution:—hill, submontane and montane belt.

Medicago lupulina L.

Distribution:—from hill to alpine belt.

Medicago minima (L.) L.

Distribution:—hill and submontane belt.

Medicago monspeliaca (L.) Trautv.

Distribution:—hill and submontane belt.

Medicago orbicularis (L.) Bartal.

Distribution:—hill and submontane belt.

Medicago polymorpha L.

Distribution:—hill and submontane belt.

Medicago prostrata Jacq. subsp. ***prostrata***

Distribution:—hill and submontane belt.

Medicago rigidula (L.) All.

Distribution:—hill and submontane belt.

A ***Medicago sativa*** L.—NAT

Distribution:—hill, submontane and montane belt.

Medicago turbinata (L.) All.

Distribution:—Prati del Sirente (Guarrera & Tammaro 1996).

A ****Medicago ×varia*** Martyn—CAS

Distribution:—Terranera! (APP 67057), Alba Fucens! (pers. obs.).

Onobrychis alba (Waldst. & Kit.) Desv. subsp. ***alba***

Distribution:—hill and submontane belt.

****Onobrychis alba*** (Waldst. & Kit.) Desv. subsp. ***pentelica*** (Hausskn.) Nyman

Distribution:—between Piani di Pezza and Cimata di Pezza!, Mt. Ocre! (APP 67905, 67906, 67999, 68158, 68159, 68162, 68168, 68169, 68170, 73300), Velino (Petriccione 1993 as *O. alba* subsp. *tenoreana*).

Onobrychis arenaria (Kit.) DC.—NC

Distribution:—surroundings of Magliano dei Marsi (Tammaro 1982),

Onobrychis caput-galli (L.) Lam.

Distribution:—hill and submontane belt.

Onobrychis viciifolia Scop.

Distribution:—hill, submontane and montane belt.

E ***Ononis cristata*** Mill. subsp. ***apennina*** Tammaro & Catonica—LC

Distribution:—diffusa soprattutto nei pascoli degli altopiani.

Ononis natrix L. subsp. ***natrix***

Distribution:—hill and submontane belt.

****Ononis ornithopodioides*** L.

Distribution:—Gole di S. Venanzio! (pers. obs.).

Ononis pusilla L. subsp. ***pusilla***

Distribution:—hill, submontane and montane belt.

Ononis reclinata L.
Distribution:—hill and submontane belt.
Ononis rotundifolia L.—NC
Distribution:—Mt. Sirente (Groves 1880; Guarrera & Tammaro 1996 from specimens collected by Groves in FI).
Ononis spinosa L. subsp. ***antiquorum*** (L.) Arcang.
Distribution:—V.ne di Sevice (Lucchese & Lattanzi 1993).
Ononis spinosa L. subsp. ***spinosa***
Distribution:—hill, submontane and montane belt.
Oxytropis campestris (L.) DC.
Distribution:—subalpine and alpine belt.
Oxytropis neglecta J.Gay ex Ten.
Distribution:—alpine belt.
E ***Oxytropis ocrensis*** F.Conti & Bartolucci—VU
Distribution:—endemic to Mt. Ocre! (Conti et al. 2018a; APP 55046, 55047, 55048, 55065) and Gran Sasso! (Conti et al. 2023b).
E, # ***Oxytropis pilosa*** (L.) DC. subsp. ***caputoi*** (Moraldo & la Valva) Brilli-Catt., Di Massimo & Gubellini—LC
Distribution:—widespread in high mountain pastures.
N ***Robinia pseudoacacia*** L.—INV
Distribution:—hill, submontane and montane belt.
Securigera cretica (L.) Lassen
Distribution:—Velino (Abbate 1903), Valle di Sevice (Lucchese & Lattanzi
Securigera varia (L.) Lassen
Distribution:—hill, submontane and montane belt.
Spartium junceum L.
Distribution:—hill and submontane belt.
Trifolium alpestre L.
Distribution:—montane to subalpine belt.
Trifolium angustifolium L. subsp. ***angustifolium***
Distribution:—hill and submontane belt.
Trifolium arvense L. subsp. ***arvense***
Distribution:—hill, submontane and montane belt.
****Trifolium aureum*** Pollich subsp. ***aureum***
Distribution:—Piani di Pezza!, between Piani di Pezza and Cimata di Pezza!, Prati del Sirente!, Val d'Arano!, Serra di Celano!, Mt. Ocre! (APP 9339, 9373, 9396, 67667, 67915, 67923, 68167), Fonte Anatella! (pers. obs.).
Trifolium campestre Schreb.
Distribution:—hill, submontane and montane belt.
****Trifolium dubium*** Sibth.
Distribution:—Campo di Rovere!, Piana di Ovindoli! (APP 34811, 73552).
Trifolium fragiferum L. subsp. ***fragiferum***
Distribution:—montane belt.
Trifolium hybridum L. subsp. ***hybridum***
Distribution:—montane belt.

A *Trifolium incarnatum* L. subsp. *incarnatum* —NAT
Distribution:—Ovindoli! (APP 73405), Forme! (pers. obs.).
Trifolium incarnatum** L. subsp. ***molinerii (Balb. ex Hornem.) Ces.
Distribution:—widespread in the Park but not previously reported.
Trifolium medium L. subsp. ***medium***
Distribution:—hill, submontane and montane belt.
Trifolium michelianum Savi
Distribution:—S. Nicola (Collarmele)! (Bartolucci et al. 2024b; APP 72043).
Trifolium micranthum Viv.
Distribution:—Prati del Sirente (Guarrera & Tammaro 1996).
Trifolium montanum L. subsp. ***rupestre*** (Ten.) Nyman
Distribution:—montaneand subalpine belt.
N ***Trifolium mutabile*** Port. subsp. ***mutabile***—CAS
Distribution:—Piana di Campo Felice! (Conti et al. 2023a; APP 67674).
Trifolium nigrescens Viv. subsp. ***nigrescens***
Distribution:—hill and submontane belt.
Trifolium noricum Wulfen subsp. ***praetutianum*** (Guss. ex Savi) Arcang.
Distribution:—alpine belt.
Trifolium ochroleucon Huds.
Distribution:—montane belt.
Trifolium phleoides Pourr. ex Willd. subsp. ***phleoides***
Distribution:—Mt. Revecena (Guarrera & Tammaro 1996).
Trifolium pratense L. subsp. ***nivale*** Ces.
Distribution:—montane to subalpine belt.
Trifolium pratense L. subsp. ***pratense***
Distribution:—hill, submontane and montane belt.
E, # ***Trifolium pratense*** L. subsp. ***semipurpureum*** (Strobl) Pignatti—LC
Distribution:—widespread in high mountain pastures.
Trifolium repens L.
Distribution:—hill, submontane and montane belt.
Trifolium resupinatum L.
Distribution:—hill, submontane and montane belt.
Trifolium rubens L.
Distribution:—Mt. Sirente (Guarrera & Tammaro 1996).
Trifolium scabrum L.
Distribution:—hill and submontane belt.
***Trifolium spadiceum** L.
Distribution:—Val d'Arano! (APP 73372).
***Trifolium squarrosum** L.
Distribution:—F. Aterno between Campana and Stiffe! (APP 73559).
Trifolium stellatum L.
Distribution:—hill and submontane belt.
Trifolium striatum L. subsp. ***striatum***
Distribution:—V.ne di Sevice (Lucchese & Lattanzi 1993), near Terranera! (APP 66628).

Trifolium strictum L.
Distribution:—Passo della Forcella (Guarrera & Tammaro 1996).
****Trifolium subterraneum*** L. s.l.
Distribution:—Piana di S. Rufina!, Carrito! (pers. obs.). At present, it is not possible to say whether it is *T. subterraneum* or *T. brachycalycinum.*
****Trifolium suffocatum*** L.
Distribution:—Castello (Fagnano Alto)! (APP 73459).
Trifolium thalii Vill.
Distribution:—subalpine and alpine belt.
Trigonella alba (Medik.) Coulot & Rabaute
Distribution:—hill, submontane and montane belt.
Trigonella altissima (Thuill.) Coulot & Rabaute
Distribution:—reported near the Park, in the neighbouring territory of Lucoli (De Santis & Soldati 2019) and likely also present in the Park.
A ***Trigonella foenum-graecum*** L.—CAS
Distribution:—Massa d'Albe, Fontecchio (Viegi et al. 1990).
Trigonella gladiata Steven ex M.Bieb.
Distribution:—Costa di Massa d'Albe (Montelucci 1958), Gole di S. Venanzio! (APP 35718), Forme! (pers. obs.).
Trigonella italica (L.) Coulot & Rabaute
Distribution:—reported near the Park, in the neighbouring territory of Lucoli (De Santis & Soldati 2019) and likely also present in the Park.
Trigonella officinalis (L.) Coulot & Rabaute
Distribution:—hill and submontane belt.
Trigonella smallii Coulot & Rabaute
Distribution:—between Celano and Ovindoli (Guarrera & Tammaro 1996).
Trigonella sulcata (Desf.) Coulot & Rabaute
Distribution:—hill, submontane and montane belt.
Trigonella wojciechowskii Coulot & Rabaute
Distribution:—reported near the Park, in the neighbouring territory of Lucoli (De Santis & Soldati 2019) and likely also present in the Park.
Tripodion tetraphyllum (L.) Fourr.
Distribution:—S. Benedetto in Perillis outside the park boundaries! (APP 68181).
Vicia angustifolia L.
Distribution:—hill and submontane belt.
Vicia bithynica (L.) L.
Distribution:—hill and submontane belt.
Vicia cassubica L.—NC
Distribution:—Macchia di Pezza before Rocca di Mezzo (Tenore 1830, 1831).
Vicia cracca L.
Distribution:—montane belt.
Vicia dasycarpa Ten.
Distribution:—above Goriano Valli (Guarrera & Tammaro 1996), Eremo della Madonna di Pietrabona! (APP 72867).
****Vicia dumetorum*** L.

Distribution:—Val d'Arano! (APP 73415).

\# ***Vicia grandiflora*** Scop.

Distribution:—Valle Majelama (Lucchese & Lattanzi 1993), Collarmele! (pers. obs.).

Vicia hybrida L.

Distribution:—hill and submontane belt.

Vicia incana Gouan

Distribution:—montane belt.

Vicia johannis Tamamsch.

Distribution:—hill and submontane belt.

****Vicia laeta*** Ces.

Distribution:—Eremo della Madonna di Pietrabona!, Cerreta di Goriano Sicoli! (APP 72869, 73004).

Vicia lathyroides L.

Distribution:—Mt. Revecena (Guarrera & Tammaro 1996).

A ***Vicia lens*** (L.) Coss. & Germ. subsp. ***lens***—CAS

Distribution:—montane belt.

****Vicia lentoides*** (Ten.) Coss. & Germ.

Distribution:—Mt. Rimagi! (APP 72983), Forme!, Capo La Maina! (pers. obs.).

Vicia lutea L.

Distribution:—hill and submontane belt.

\# ***Vicia onobrychioides*** L.

Distribution:—montane belt.

\# ***Vicia pannonica*** Crantz subsp. ***striata*** (M.Bieb.) Nyman

Distribution:—hill, submontane and montane belt.

\# ***Vicia peregrina*** L.

Distribution:—hill and submontane belt.

Vicia pseudocracca Bertol.

Distribution:—Mt. Ventrino (Guarrera & Tammaro 1996).

Vicia sativa L.

Distribution:—hill and submontane belt.

Vicia segetalis Thuill.

Distribution:—Forme (Lucchese & Lattanzi 1993), Goriano Sicoli! (APP 73567).

Vicia sepium L.

Distribution:—montane belt.

Vicia tenuifolia Roth subsp. ***tenuifolia***

Distribution:—hill and submontane belt.

\# ***Vicia villosa*** Roth

Distribution:—hill, submontane and montane belt.

FAGACEAE

AL ***Castanea sativa*** Mill.—NAT

Distribution:—hill, submontane and montane belt.

\# ***Fagus sylvatica*** L.

Distribution:—hill, submontane and montane belt.

Quercus* ×*hispanica Lam.
Distribution:—Pratelle di Fagnano (Tammaro 1984 as *Q. crenata*), Saravastrello on the slopes of Mt. Rozza! (APP 34659).

\# ***Quercus cerris*** L.
Distribution:—hill, submontane and montane belt.

Quercus frainetto Ten.—D
Distribution:—from Bocca di Teve to Passo le Forche, Pié di Sevice (Lucchese & Lattanzi 1993).

\# ***Quercus ilex*** L.
Distribution:—hill, submontane and montane belt.

Quercus petraea (Matt.) Liebl. subsp. *petraea*—D
Distribution:—Velino (Avena & Blasi 1980).

\# ***Quercus pubescens*** Willd. subsp**.** ***pubescens***
Distribution:—hill, submontane and montane belt.

GENTIANACEAE

Blackstonia perfoliata (L.) Huds. subsp. ***perfoliata***
Distribution:—hill and submontane belt.

Centaurium erythraea Rafn subsp. ***erythraea***
Distribution:—hill, submontane and montane belt.

Centaurium pulchellum (Sw.) Hayek ex Hand.-Mazz., Stadlm., Janch. & Faltis subsp. ***pulchellum***
Distribution:—laghetto at Prati del Sirente (Guarrera & Tammaro 1996).

\# ***Gentiana cruciata*** L. subsp. ***cruciata***
Distribution:—montane belt.

\# ***Gentiana dinarica*** (Beck) Beck
Distribution:—montane and subalpine belt.

\# ***Gentiana lutea*** L. subsp. ***lutea***—NT
Distribution:—montane belt.

\# ***Gentiana nivalis*** L.
Distribution:—montane belt.

Gentiana orbicularis Schur
Distribution:—montane and subalpine belt.

Gentiana pneumonanthe L. subsp. ***pneumonanthe***—VU
Distribution:—Campo di Rovere!, Val d'Arano! (Ciaschetti 2003; APP 28662, 57250, 68010, 68011), Conche di Ovindoli!, Piana di Ovindoli near il laghetto! (APP 66780, 67777).

Gentiana utriculosa L.
Distribution:—montane and subalpine belt.

Gentiana verna L. subsp. *tergestina* (Beck) Hayek—NC
Distribution:—Velino, Mt. Sirente (Tenore 1830, 1831 as *G. aestiva*; Groves 1880 as *G. aestiva*; Martelli 1904 as *G. aestiva*; Steinberg 1953 as *G. verna* var. *aestiva*). Montelucci (1958) does not confirm its presence on the Velino.

\# ***Gentiana verna*** L. subsp. ***verna***
Distribution:—montane and subalpine belt.

E, # **Gentianella columnae** (Ten.) Holub—LC
Distribution:—widespread in high mountain pastures.
Gentianopsis ciliata (L.) Ma subsp. **ciliata**
Distribution:—montane belt.

GERANIACEAE

Erodium acaule (L.) Bech. & Thell.—NC
Distribution:—Celano (Tenore 1842 as *E. romanum*).

E **Erodium alpinum** (Burm.f.) L'Hér.—LC
Distribution:—widespread in high montane pastures.
Erodium ciconium (L.) L'Hér.
Note:—Guarrera & Tammaro (1996) reported also *E. moschatum* (L.) L'Hér. for Aielli and surrounding areas. This report is to be referred to *E. ciconium* on the basis of ours revision of the specimens preserved in the Guarrera herbarium.

Erodium cicutarium (L.) L'Hér.
Distribution:—hill, submontane and montane belt.
***Erodium malacoides** (L.) L'Hér. subsp. **malacoides**
Distribution:—Acciano! (APP 74176), Fontecchio!, Fagnano Alto in loc. Ripa!, Succiano! (pers. obs.).

E, # **Geranium austroapenninum** Aedo—LC
Distribution:—Mt. Sirente!, Velino (Groves 1880 as *G. cinereum*; Petriccione 1993 as *G. cinereum* subsp. *subcaulescens*; Guarrera & Tammaro 1996 as *G. cinereum*; Aedo 1996; APP 67199, 67394).
Geranium columbinum L.
Distribution:—montane belt.
Geranium dissectum L.
Distribution:—hill and submontane belt.

Geranium lucidum L.
Distribution:—montane belt.
***Geranium macrorrhizum** L.
Distribution:—Mt. Ocre! (APP 15809).

Geranium molle L.
Distribution:—hill, submontane and montane belt.

Geranium nodosum L.
Distribution:—montane belt.

Geranium purpureum Vill.
Distribution:—hill, submontane and montane belt.
Geranium pusillum L.
Distribution:—hill, submontane and montane belt.
Geranium pyrenaicum Burm.f. subsp. **pyrenaicum**
Distribution:—hill, submontane and montane belt.

Geranium reflexum L.
Distribution:—montane belt.

Geranium robertianum L.
Distribution:—hill, submontane and montane belt.

Geranium rotundifolium L.
Distribution:—hill, submontane and montane belt.
Geranium sanguineum L.
Distribution:—hill, submontane and montane belt.
Geranium sylvaticum L.
Distribution:—montane belt.
Geranium tuberosum L. subsp. ***tuberosum***
Distribution:—hill, submontane and montane belt.

GROSSULARIACEAE
Ribes alpinum L.
Distribution:—montane belt.
Ribes multiflorum Kit. ex Roem. & Schult. subsp. ***multiflorum***
Distribution:—montane belt.
Ribes uva-crispa L. subsp. ***uva-crispa***
Distribution:—montane belt.

HALORAGACEAE
Myriophyllum spicatum L.
Distribution:—hill, submontane and montane belt.

HELIOTROPIACEAE
Heliotropium europaeum L.
Distribution:—hill and submontane belt.

HYDRANGEACEAE
AR ****Philadelphus coronarius*** L.—CAS
Distribution:—Alba Fucens! (pers. obs.).

HYDROPHYLLACEAE
N ***Phacelia tanacetifolia*** Benth.—CAS
Distribution:—Gole di S. Venanzio! (Conti et al. 2016; APP 55566).

HYPERICACEAE
N ****Hypericum calycinum*** L.—NAT
Distribution:—Molina Aterno!, Pedicciano!, crossroad for Roccapreturo!, cemetery of Castelvecchio Subequo! (APP 28409, 28410, 68338; pers. obs.).
Hypericum hyssopifolium Chaix
Distribution:—montane to subalpine belt.
Hypericum montanum L.
Distribution:—hill, submontane and montane belt.
Hypericum perfoliatum L.
Distribution:—hill, submontane and montane belt.
Hypericum perforatum L. subsp. ***perforatum***
Distribution:—hill, submontane and montane belt.

Hypericum perforatum L. subsp. ***veronense*** (Schrank) Ces.
Distribution:—hill, submontane and montane belt.

\# ***Hypericum richeri*** Vill. subsp. ***grisebachii*** (Boiss.) Nyman
Distribution:—Capo di Pezza, Fossa del Puzzillo (Blasi et al. 1992 as *H. richeri*; Stanisci 1997 as *H. richeri*), Valle Majelama, V.ne di Teve, between Rif. Sebastiani and Colle dell'Orso, Cimata di Puzzillo, Mt. Velino! (Lucchese & Lattanzi 1993 as *H. richeri*; Petriccione 1993 as *H. richeri;* Conti et al. in press; APP 34645), Campo Felice (De Santis & Soldati 2009 as *H. richeri* subsp. *richeri*). All Central Apennine specimens of *H. richeri* have been attributed to the subsp. *grisebachii*, new to Italy (Conti et al. in press).

Hypericum tetrapterum Fr.
Distribution:—hill, submontane and montane belt.

IRIDACEAE

****Chamaeiris foetidissima*** (L.) Medik. (*Iris foetidissima* L.)
Distribution:—near Molina Aterno! (APP 28402, 38408), Alba Fucens! (pers. obs.).

E ***Crocus biflorus*** Mill.—LC
Distribution:—prati di Rocca di Mezzo! (Guarrera & Tammaro 1996; APP 70983), near Terranera! (APP 46546, 67020).

\# ***Crocus neapolitanus*** (Ker Gawl.) Loisel.
Distribution:—hill, submontane and montane belt.

\# ***Crocus variegatus*** Hoppe & Hornsch.
Distribution:—hill, submontane and montane belt.

C ***Gladiolus byzantinus*** Mill.
Distribution:—hill and submontane belt.

C ***Gladiolus italicus*** Mill.
Distribution:—hill and submontane belt.

A ***Iris germanica*** L.—NAT
Distribution:—hill, submontane and montane belt.

Iris lutescens Lam.
Distribution:—Mt. Rozza! (Conti 1998; Conti et al. 1999; APP 40622).

E, # ***Iris marsica*** I.Ricci & Colas.—NT
Distribution:—Valle di Sevice (Lucchese & Lattanzi 1993), Secinaro!, Tione degli Abruzzi!, Terranera!, Fonteavignone!, Prati del Sirente! (Conti & Di Martino 2021; APP 28492, 28493, 59699, 59924, 59925, 59926, 59927, 59928). Species included in All. IV of the Habitats Directive 92/43/EEC.

****Romulea bulbocodium*** (L.) Sebast. & Mauri
Distribution:—near Magliano! (pers. obs.).

JUGLANDACEAE

N ***Juglans nigra*** L.—CAS
Distribution:—Rocca di Mezzo (Galasso et al. 2019), Tione degli Abruzzi! (APP 28250, 28251).

AR, # ***Juglans regia*** L.—CAS

Distribution:—hill and submontane belt.

JUNCACEAE

Juncus acutiflorus Ehrh. ex Hoffm.

Distribution:—Piano S. di Rufino! (APP 62187).

Juncus articulatus L. subsp. ***articulatus***

Distribution:—hill, submontane and montane belt.

Juncus atratus Krock.—CR

Distribution:—Prato della Madonna! (Conti et al. 2023a; APP 60340, 60341, 60342).

Juncus bufonius L.

Distribution:—hill and submontane belt.

Juncus compressus Jacq.

Distribution:—hill, submontane and montane belt.

Juncus conglomeratus L.

Distribution:—Campo di Rovere! (pers. obs.).

Juncus inflexus L. subsp. ***inflexus***

Distribution:—hill, submontane and montane belt.

Luzula alpina Hoppe

Distribution:—Campo di Rovere! (APP 34793).

Luzula campestris (L.) DC. subsp. ***campestris***

Distribution:—montane belt.

Luzula forsteri (Sm.) DC.

Distribution:—hill, submontane and montane belt.

Luzula multiflora (Ehrh.) Lej. subsp. ***multiflora***

Distribution:—from hill and submontane to subalpine belt.

Luzula spicata (L.) DC. subsp. ***bulgarica*** (Chrtek & Křísa) Gamisans

Distribution:—hill, submontane and montane belt.

Luzula sylvatica (Huds.) Gaudin subsp. ***sieberi*** (Tausch) K.Richt.

Distribution:—from hill and submontane to subalpine belt.

Oreojuncus monanthos (Jacq.) Záv.Drábk. & Kirschner (*Juncus monanthos* Jacq.)

Distribution:—from montane to subalpine belt.

JUNCAGINACEAE

Triglochin palustris L.

Distribution:—Altopiano delle Rocche (Ballelli & Pedrotti 1979; Guarrera & Tammaro 1996), Campo di Rovere! (Ciaschetti et al. 2024; APP 28665, 68002), Piana di Ovindoli in loc. Le Conche! (APP 66547, 66615), between Rocca di Mezzo and Piani di Pezza! (APP 66669, 66684).

LAMIACEAE

Ajuga chamaepitys (L.) Schreb. subsp. ***chamaepitys***

Distribution:—hill, submontane and montane belt.

Ajuga chamaepitys (L.) Schreb. subsp. *chia* (Schreb.) Arcang.—NC

Distribution:—Mt. Sirente (Groves 1880).

\# ***Ajuga reptans*** L.

Distribution:—hill, submontane and montane belt.

E, # ***Ajuga tenorei*** C.Presl—LC

Distribution:—uncommon in mountain pastures.

Ballota nigra L. subsp. ***meridionalis*** (Bég.) Bég.

Distribution:—hill, submontane and montane belt.

E, # ***Betonica alopecuros*** L. subsp. ***divulsa*** (Ten.) Bartolucci & Peruzzi—LC

Distribution:—common in high-altitude rocky slopes.

\# ***Betonica officinalis*** L.

Distribution:—hill, submontane and montane belt.

Clinopodium grandiflorum (L.) Kuntze

Distribution:—montane belt.

\# ***Clinopodium menthifolium*** (Host) Merino subsp. ***ascendens*** (Jord.) Govaerts

Distribution:—Le Coste (Rosciolo) (Avena & Blasi 1975).

Clinopodium nepeta (L.) Kuntze subsp. ***nepeta***

Distribution:—hill, submontane and montane belt.

****Clinopodium nepeta*** (L.) Kuntze subsp. ***spruneri*** (Boiss.) Bartolucci & F.Conti

Distribution:—Recolle! and loc. Macchione! (APP 28340, 28355, 29952).

Clinopodium vulgare L. subsp. ***vulgare***

Distribution:—hill, submontane and montane belt.

\# ***Dracocephalum officinale*** (L.) Y.I.Chen & B.T.Drew (*Hyssopus officinalis* L.; *Hyssopus officinalis* L. subsp. *aristatus* (Godr.) Nyman; *Hyssopus officinalis* L. subsp. *pilifer* (Pant.) Murb.)

\# ***Galeopsis angustifolia*** Ehrh. ex Hoffm. subsp. ***angustifolia***

Distribution:—hill, submontane and montane belt.

Galeopsis bifida Boenn.

Distribution:—Conche di Ovindoli! (Conti et al. 2025a; APP 72961).

Galeopsis ladanum L.

Distribution:—montane belt.

Galeopsis tetrahit L.

Distribution:—hill, submontane and montane belt.

****Glechoma hirsuta*** Waldst. & Kit.

Distribution:—Fontecchio! (APP 68276).

Lamium album L. subsp. ***album***

Distribution:—Mt. Sirente (Veri & Tammaro 1980), Piani di Pezza (Lucchese & Lattanzi 1993), Campo Felice (De Santis & Soldati 2011).

Lamium amplexicaule L.

Distribution:—hill, submontane and montane belt.

Lamium bifidum Cirillo subsp. ***balcanicum*** Velen.

Distribution:—Prati del Sirente! (Conti et al. 2008a; APP 27771, 67165), from Campo di Rovere to Rif. La Vecchia! (APP 72778).

Lamium bifidum Cirillo subsp. ***bifidum***

Distribution:—hill and submontane belt.

****Lamium flexuosum*** Ten. subsp. ***flexuosum***

Distribution:—Mt. Briccialone! (APP 67201).

Lamium galeobdolon (L.) L.

Distribution:—Piani di Pezza (Ciaschetti et al. 2006). Not found by us in the Park. We didn't trace herbarium samples for the identification of the subspecies.

Lamium garganicum L. subsp. ***longiflorum*** (Ten.) Kerguélen

Distribution:—hill, submontane and montane belt.

Lamium garganicum L. subsp. ***striatum*** (Sm.) Hayek

Distribution:—from montane to subalpine belt.

Lamium maculatum L.

Distribution:—hill, submontane and montane belt.

Lamium purpureum L.

Distribution:—hill, submontane and montane belt.

****Lycopus europaeus*** L.

Distribution:—F. Aterno below Fossa! (APP 26751, 26752, 26753), Molina Aterno! (APP 28387, 28388).

Marrubium incanum Desr.

Distribution:—hill, submontane and montane belt.

Marrubium vulgare L.

Distribution:—hill, submontane and montane belt.

C ***Melissa officinalis*** L. subsp. ***officinalis***

Distribution:—hill and submontane belt.

Melittis melissophyllum L. subsp. ***melissophyllum***

Mentha ×piperita L.—NC

Distribution:—Mt. Sirente (Groves 1880).

Mentha aquatica L. subsp. ***aquatica***

Distribution:—hill, submontane and montane belt.

Mentha arvensis L.

Distribution:—montane belt.

Mentha longifolia (L.) L.

Distribution:—hill, submontane and montane belt.

****Mentha microphylla*** K. Koch

Distribution:—between Fonte Salecchia and Fonte del Campo! (APP 73539).

Mentha pulegium L. subsp. ***pulegium***

Distribution:—hill, submontane and montane belt.

Mentha spicata L.

Distribution:—hill, submontane and montane belt.

E ***Micromeria graeca*** (L.) Benth. ex Rchb. subsp. ***tenuifolia*** (Ten.) Nyman—LC

Distribution:—common in low-altitude cliffs.

Micromeria juliana (L.) Benth. ex Rchb.

Distribution:—"alla salita di Raiano" from a specimen collected by Gussone (Grande 1925 as *Satureja juliana*), S. Maria in Valle Porclaneta (Vannicelli Casoni et al. 1998), Fontecchio! (pers. obs.).

Nepeta cataria L.

Distribution:—Velino (Petriccione 1993).

Nepeta nuda L. subsp. ***nuda***

Distribution:—montane belt.

Origanum vulgare L. subsp. ***vulgare***

Distribution:—hill, submontane and montane belt.

Phlomis fruticosa L.

Distribution:—Colle del Rascito!, Colle della Forchetta! (Pirone 1995, 1996; APP 61823; pers. obs.).

Phlomis herba-venti L. subsp. ***herba-venti***

Distribution:—hill and submontane belt.

****Prunella* ×*intermedia*** Link

Distribution:—Serra di Celano!, Ovindoli! (pers. obs.).

\# ***Prunella laciniata*** (L.) L.

Distribution:—hill, submontane and montane belt.

Prunella vulgaris L. subsp. ***vulgaris***

Distribution:—hill, submontane and montane belt.

Salvia aethiopis L.—EN

Distribution:—Forme! (Conti et al. 2011a; APP 43185).

Salvia argentea L.

Distribution:—hill, submontane and montane belt.

****Salvia clandestina*** L.

Distribution:—Gole di S. Venanzio!, Collarmele!, Carrito!, Goriano Valli!, Acciano! (pers. obs.).

Salvia glutinosa L.

Distribution:—hill, submontane and montane belt.

E ***Salvia haematodes*** L.—LC

Distribution:—near Rosciolo (Del Carratore & Garbari 2003 from a specimen collected by Martelli), Aielli and surroundings (Guarrera & Tammaro 1996).

Salvia officinalis L. subsp. ***officinalis*** var. ***angustifolia*** Ten.

Distribution:—Gole di San Venanzio (Pirone et al. 2018), between Frascara and Castello! (APP 74246, 74247). The population found between Frascara and Castello mostly has very narrow leaves and often the lamina has two lobes at the base, characteristics that would be typical of *Salvia officinalis* var. *angustifolia* Ten. However, examination of aerial photographs taken about ten years ago reveals that the site developed over a rectangular area and the individuals were aligned, which would suggest an anthropogenic planting.

\# ***Salvia pratensis*** L.

Distribution:—hill, submontane and montane belt.

Salvia rosmarinus Spenn. (*Rosmarinus officinalis* L.)

Distribution:—hill and submontane belt.

Salvia sclarea L.

Distribution:—hill and submontane belt.

Salvia verbenaca L.

Distribution:—hill and submontane belt.

Salvia virgata Jacq.

Distribution:—presso Alba, stazione di Fontecchio, between Magliano dei Marsi and Torano (Del Carratore & Garbari 2003).

A **Satureja hortensis** L.—NAT

Distribution:—Rocca di Mezzo! (Conti et al. 2019a).

Satureja montana L. subsp. **montana**

Distribution:—hill, submontane and montane belt.

Scutellaria alpina L. subsp. **alpina**

Distribution:—montane and alpine belt.

Scutellaria columnae All. subsp. **columnae**

Distribution:—hill, submontane and montane belt.

Scutellaria galericulata L.

Distribution:—Altopiano delle Rocche!, Piano di Ovindoli (Ciaschetti et al. 2005; APP 11283), Paludi di Celano! (APP 56202), Campo di Rovere! (pers. obs.).

Stachys annua (L.) L. subsp. **annua**

Distribution:—hill, submontane and montane belt.

Stachys germanica L. subsp. **germanica**

Distribution:—hill and submontane belt.

Stachys germanica L. subsp. **salviifolia** (Ten.) Gams

Distribution:—hill and submontane belt.

Stachys heraclea All.

Distribution:—montane belt.

E, # **Stachys italica** Mill.—LC

Distribution:—common in thermophilous garrigue.

Stachys montana (L.) Peruzzi & Bartolucci subsp. **montana**

Distribution:—hill, submontane and montane belt.

***Stachys palustris** L.

Distribution:—Molina Aterno! (APP 28383).

Stachys recta L. subsp. **labiosa** (Bertol.) Briq.

Distribution:—from montane to subalpine belt.

Stachys recta L. subsp. **recta**

Distribution:—hill and submontane belt.

Stachys recta L. subsp. **subcrenata** (Vis.) Briq.

Distribution:—hill and submontane belt.

Stachys romana (L.) E.H.L.Krause

Distribution:—hill and submontane belt.

Stachys sylvatica L.

Distribution:—hill, submontane and montane belt.

Stachys tymphaea Hausskn.

Distribution:—from montane to subalpine belt.

Teucrium botrys L.

Distribution:—hill, submontane and montane belt.

Teucrium capitatum L. subsp. **capitatum**

Distribution:—hill and submontane belt.

Teucrium chamaedrys L. subsp. **chamaedrys**

Distribution:—hill, submontane and montane belt.

Teucrium flavum L. subsp. **flavum**

Distribution:—hill and submontane belt.

Teucrium montanum L.
Distribution:—montane belt.

Teucrium scordium L. subsp. ***scordioides*** (Schreb.) Arcang.
Distribution:—Mt. Sirente (Groves 1880), Lago di Molina! (Conti 1998 da reperti in FI), Lago di Civita! (APP 34858). Reports for Campo Felice (De Santis & Soldati 2011, 2019), Gole di Celano (probably Val d'Arano) (Frizzi et al. 1996; Guarrera & Tammaro 1996), Val d'Arano and Piana di Ovindoli (Ciaschetti et al. 2005) are probably to be referred to *T. scordium* subsp. *scordium* .

Teucrium scordium L. subsp. ***scordium***
Distribution:—Gole di Celano (probably Val d'Arano) (Frizzi et al. 1996; Guarrera & Tammaro 1996 as *T. scordium* subsp. *scordioides*), Piana di Ovindoli! (Conti 1998; APP 66505, 66567), Campo di Rovere!, Val d'Arano! (APP 28597, 28670).

Teucrium siculum (Raf.) Guss. subsp. ***siculum***
Distribution:—hill and submontane belt.

Thymus longicaulis C.Presl subsp. ***longicaulis***
Distribution:—hill, submontane and montane belt.

Thymus moesiacus Velen.
Distribution:—hill, submontane and montane belt.

Thymus praecox Opiz subsp. ***polytrichus*** (A.Kern ex Borbás) Jalas
Distribution:—montane and alpine belt.

Thymus striatus Vahl subsp. ***acicularis*** (Waldst. & Kit.) Ronniger
Distribution:—hill, submontane and montane belt.

Thymus striatus Vahl subsp. ***striatus***
Distribution:—Mt. Cefalone!, Mt. Rozza!, V.ne di Sevice! (APP 67431, 67432; pers. obs.). Confirmed for the flora of Abruzzo.

Thymus vulgaris L. subsp. ***vulgaris***
Distribution:—Colle Putano (Castelvecchio Subequo)!, Monte Colle di More (Molina Aterno)! (Bartolucci et al. 2013; APP 50507, 73022, 73023, 73024).

****Thymus zygiformis*** Heinr.Braun—DD
Distribution:—Campo Felice!, Mt. Sirente! (APP 68085, 68290), from Rovere to Rif. La Vecchia (APP 72795), Piani di Pezza!, Cimata di Pezza! (pers. obs.).

Ziziphora acinos (L.) Melnikov
Distribution:—hill and submontane belt.

Ziziphora granatensis (Boiss. & Reut.) Melnikov subsp. ***alpina*** (L.) Bräuchler & Gutermann
Distribution:—from montane to subalpine belt.

Ziziphora granatensis (Boiss. & Reut.) Melnikov subsp. ***granatensis***
Note:—common from from hill and submontane to montane belt. the report of *Z. suaveolens* (Sm.) Melnikov (Lucchese & Lattanzi 1993) for Valle Pioppi should probably to be referred to this taxon.

LENTIBULARIACEAE

E ***Pinguicula vulgaris*** L. subsp. ***vestina*** F.Conti & Peruzzi—EN
Distribution:—Campo di Rovere! (Conti & Peruzzi 2006; APP 34713).

LILIACEAE

Fritillaria montana Hoppe ex W.D.J.Koch—NT
Distribution:—montane belt.

Gagea bohemica (Zauschn.) Schult. & Schult.f.
Distribution:—hill, submontane and montane belt.

\# ***Gagea fragifera*** (Vill.) Ehr.Bayer & G.López
Distribution:—from montane to subalpine belt.

Gagea lacaitae A.Terracc.—LC
Distribution:—Terranera! (Peruzzi & Bartolucci 2006; pers. obs.).

Gagea luberonensis J.-M.Tison—LC
Distribution:—Terranera! (Peruzzi & Bartolucci 2006; pers. obs.).

\# ***Gagea lutea*** (L.) Ker Gawl.
Distribution:—montane belt.

Gagea minima (L.) Ker Gawl.
Distribution:—V. di Fundoli! (Bartolucci et al. 2025; APP 73370, 73371).

Gagea pratensis (Pers.) Dumort.
Distribution:—montane belt.

Gagea ramulosa A.Terracc.
Distribution:—Terranera! (Peruzzi & Bartolucci 2006; APP 20580, 44393).

\# ***Gagea villosa*** (M.Bieb.) Sweet
Distribution:—hill, submontane and montane belt.

\# ***Lilium bulbiferum*** L. subsp. ***croceum*** (Chaix) Jan
Distribution:—hill, submontane and montane belt.

\# ***Lilium martagon*** L.
Distribution:—montane belt.

N ****Tulipa agenensis*** Redouté—CAS
Distribution:—Corbellino! (pers. obs.).

Tulipa pumila Moench
Distribution:—Ovindoli! (Guarrera & Tammaro 1996; pers. obs.), Altopiano delle Rocche! (APP 26958, 26959, 68114).

LINACEAE

\# ***Linum alpinum*** Jacq.
Distribution:—montane belt.

\# ***Linum capitatum*** Kit. ex Schult. subsp. ***serrulatum*** (Bertol.) Hartvig
Distribution:—from montane to subalpine belt.

Linum catharticum L. subsp. ***catharticum***
Distribution:—montane belt.

\# ***Linum catharticum*** L. subsp. ***suecicum*** Hayek
Distribution:—montane belt.

Linum narbonense L.
Distribution:—Campo Felice! (Lucchese & Lattanzi 1993; Conti 1998 from a specimen collected by Rosati; APP 62784), Prati del Sirente! (APP 60253,

Linum strictum L.

Distribution:—hill and submontane belt.
\# ***Linum tenuifolium*** L.
Distribution:—hill, submontane and montane belt.
\# ***Linum tommasinii*** (Rchb.) Nyman
Distribution:—hill, submontane and montane belt.
Linum usitatissimum L. subsp. ***angustifolium*** (Huds.) Thell.
Distribution:—hill and submontane belt.

LORANTHACEAE
Loranthus europaeus Jacq.
Distribution:—hill and submontane belt.

LYTHRACEAE
Lythrum salicaria L.
Distribution:—hill and submontane belt.
Peplis portula L.
Distribution:—Altopiano delle Rocche! (Conti et al. 2006; pers. obs.), Campo di Rovere! (APP 28657).

MALVACEAE
A ****Abutilon theophrasti*** Medik.—NAT
Distribution:—Forme! (pers. obs.).
N ***Alcea biennis*** Winterl subsp. ***biennis***—NAT
Distribution:—hill and submontane belt.
N ***Hibiscus trionum*** L.—NAT
Distribution:—several locations along the Aterno Valley (pers. obs.).
Malope malacoides L. subsp. *malacoides*—NC
Distribution:—Mt. Sirente (Groves 1880).
Malva moschata L.
Distribution:—hill, submontane and montane belt.
\# ***Malva neglecta*** Wallr.
Distribution:—hill and submontane belt.
Malva nicaeensis All.
Distribution:—Forme (Lucchese & Lattanzi 1993), Velino (Petriccione 1993).
Malva setigera K.F.Schimp. & Spenn.
Distribution:—hill and submontane belt.
Malva sylvestris L.
Distribution:—common from hill and submontane to montane belt.
Malva thuringiaca (L.) Vis.—NC
Distribution:—Mt. Sirente (Groves 1880).
\# ***Tilia cordata*** Mill.
Distribution:—hill and submontane belt.
Tilia platyphyllos Scop. subsp. ***cordifolia*** (Besser) C.K.Schneid.
Distribution:—hill and submontane belt.

MELANTHIACEAE

\# ***Paris quadrifolia*** L.

Distribution:—montane belt.

MENYANTHACEAE

****Menyanthes trifoliata*** L.

Distribution:—Piano di Ovindoli in loc. Le Conche! (APP 66492).

MORACEAE

Ficus carica L.

Distribution:—hill and submontane belt.

N ****Maclura pomifera*** (Raf.) C.K.Schneid.—CAS

Distribution:—Aielli along the road leading up to Prato Cerro! (pers. obs.)

A ***Morus nigra*** L.—CAS

NYCTAGINACEAE

N ****Mirabilis jalapa*** L.—CAS

Distribution:—Succiano! (pers. obs.).

NYMPHAEACEAE

N ***Nymphaea ×marliacea*** Lat.-Marl.—CAS

Distribution:—Lago di Bominaco! (APP 73532; Conti et al. in press).

OLEACEAE

****Fraxinus angustifolia*** Vahl subsp. ***oxycarpa*** (M.Bieb. ex Willd.) Franco & Rocha Afonso

Distribution:—F. Aterno near Tione! (APP 28239).

\# ***Fraxinus excelsior*** L. subsp. ***excelsior***

Distribution:—montane belt.

\# ***Fraxinus ornus*** L. subsp. ***ornus***

Distribution:—hill, submontane and montane belt.

\# ***Ligustrum vulgare*** L.

Distribution:—hill and submontane belt.

A ****Olea europaea*** L.—CAS

Distribution:—Gole di S.Venanzio near Raiano! (pers. obs.).

Phillyrea latifolia L.

Distribution:—hill and submontane belt.

N ****Syringa vulgaris*** L.—CAS

Distribution:—Castelnuovo in loc. Vicenne! (APP 62204), Succiano!, Rosciolo!, Alba Fucens!, between Massa d'Albe and Forme! (pers. obs.).

ONAGRACEAE

\# ***Chamaenerion angustifolium*** (L.) Scop.

Distribution:—montane belt.

Chamaenerion dodonaei (Vill.) Schur ex Fuss

Distribution:—montane belt.

Circaea lutetiana L. subsp. ***lutetiana***

Distribution:—montane belt.

Epilobium hirsutum L.

Distribution:—hill, submontane and montane belt.

Epilobium montanum L.

Distribution:—montane belt.

Epilobium palustre L.

Distribution:—Campo Felice, Campo di Rovere (De Santis & Soldati 2011; Ciaschetti et al. 2024).

Epilobium parviflorum Schreb.

Distribution:—common from hill and submontane to montane belt.

ORCHIDACEAE

Anacamptis coriophora (L.) R.M.Bateman, Pridgeon & M.W.Chase

Distribution:—hill and submontane belt.

Anacamptis laxiflora (Lam.) R.M.Bateman, Pridgeon & M.W.Chase

Distribution:—Velino (Avena & Blasi 1975), Aielli (Guarrera & Tammaro 1996), Piana di Ovindoli in loc. Le Conche! (pers. obs.).

Anacamptis morio (L.) R.M.Bateman, Pridgeon & M.W.Chase

Distribution:—common from hill and submontane to montane belt.

Anacamptis papilionacea (L.) R.M.Bateman, Pridgeon & M.W.Chase

Distribution:—Raiano (Pezzetta 2016), between Castelvecchio Subequo and Forca Caruso! (pers. obs.).

Anacamptis pyramidalis (L.) Rich.

Distribution:—hill, submontane and montane belt.

Cephalanthera damasonium (Mill.) Druce

Distribution:—hill, submontane and montane belt.

Cephalanthera longifolia (L.) Fritsch

Distribution:—hill, submontane and montane belt.

Cephalanthera rubra (L.) Rich.

Distribution:—hill, submontane and montane belt.

Coeloglossum viride (L.) Hartm.

Distribution:—from montane to subalpine belt.

Corallorhiza trifida Châtel.

Distribution:—montane belt.

Dactylorhiza gervasiana (Tod.) H.Baumann & Künkele (*D. maculata* (L.) Soó subsp. *saccifera* auct.)

Note:—the reports of *D. maculata* (L.) Soó subsp. *fuchsii* (Druce) Hyl. should to be referred to this taxon. Common species from hill and submontane to montane belt.

Dactylorhiza incarnata (L.) Soó subsp. ***incarnata***

Distribution:—montane belt.

Dactylorhiza sambucina (L.) Soó

Distribution:—common from hill and submontane to montane belt.

Epipactis atrorubens (Hoffm.) Besser
Distribution:—montane belt.
Epipactis exilis P.Delforge—LC
Distribution:—Mt. Rotondo (De Angelis & Scacchi 1990 as *E. persica*).
Epipactis helleborine (L.) Crantz
Distribution:—common from hill and submontane to montane belt.
Epipactis microphylla (Ehrh.) Sw.
Distribution:—hill, submontane and montane belt.
Epipactis palustris (L.) Crantz—NT
Distribution:—Rocca di Mezzo! (Pezzetta 2016; APP 66677, 72974, 73468), Piana di Ovindoli!, Campo di Rovere!, Val d'Arano! (pers. obs.).
Epipogium aphyllum Sw.
Distribution:—Sacco Sirente! (Fumanti et al. 1986; pers. obs.), Rocca di Mezzo (Pezzetta 2016).
Gymnadenia conopsea (L.) R.Br.
Distribution:—common from montane to subalpine belt.
****Gymnadenia densiflora*** (Wahlenb.) A.Dietr.
Distribution:—Ovindoli, Rocca di Cambio (G. Picone in litt.).
Himantoglossum adriaticum H.Baumann—LC
Distribution:—Molina Aterno, Goriano Valli (Guarrera & Tammaro 1996), Massa d'Albe, Castelvecchio Subequo, Mt. Sirente, Mt. Velino, Rocca di Mezzo (Pezzetta 2016), Terranera! and Mt. della Cerreta (pers. obs.). Species included in All. II and IV of the Habitats Directive 92/43/EEC
Limodorum abortivum (L.) Sw.
Distribution:—common from hill and submontane to montane belt.
Neotinea tridentata (Scop.) R.M.Bateman, Pridgeon & M.W.Chase
Distribution:—common from hill and submontane to montane belt.
Neotinea ustulata (L.) R.M.Bateman, Pridgeon & M.W.Chase
Distribution:—montane belt.
Neottia nidus-avis (L.) Rich.
Distribution:—common from hill and submontane to montane belt.
Neottia ovata (L.) Bluff & Fingerh.
Distribution:—common from hill and submontane to montane belt.
Nigritella widderi Teppner & E.Klein
Distribution:—Mt. Sirente!, Mt. Coccorello (Rossi et al. 1987; pers. obs.), Cimata di Pezza! (R. Soldati in litt.; pers. obs.). Petriccione's report (1993) refers to Monti della Duchessa.
Ophrys apifera Huds.
Distribution:—common from hill and submontane to montane belt.
Ophrys bertolonii Moretti subsp. ***bertolonii*** —LC
Distribution:—common from hill and submontane to montane belt.
E ****Ophrys crabronifera*** Mauri subsp. ***crabronifera*** —LC
Distribution:—Gole di S. Venanzio! (APP 66905).
Ophrys fusca Link subsp. ***funerea*** (Viv.) Arcang.
Distribution:—Massa d’Albe (Pezzetta 2016).

E *__Ophrys fusca__* Link subsp. *__lucana__* (P.Delforge, Devillers-Tersch. & Devillers) Kreutz

Distribution:—Aielli (R. Soca in litt.).

E ***Ophrys holosericea*** (Burm.f.) Greuter subsp. ***appennina*** (Romolini & Soca) Kreutz—LC

Distribution:—Magliano dei Marsi (Pezzetta 2016), Aielli (R. Soca in litt.).

Ophrys holosericea (Burm.f.) Greuter subsp. ***dinarica*** (Kranjčev & P.Delforge) Kreutz

Distribution:—common from hill and submontane to montane belt.

Ophrys incubacea Bianca subsp. ***incubacea***

Distribution:—hill and submontane belt.

E ***Ophrys passionis*** Sennen subsp. ***majellensis*** (Helga Daiss & Herm.Daiss) Romolini & Soca

Distribution:—Aielli (Pezzetta 2016).

E ***Ophrys promontorii*** O.Danesch & E.Danesch—LC

Distribution:— S. Benedetto in Perillis (Kalteisen & Reinhard 1987), Raiano (Pezzetta 2016), Acciano! (pers. obs.), Castelvecchio Subequo! (APP 73016).

Ophrys sphegodes Mill. subsp. ***ausonia*** (Devillers, Devillers-Tersch. & P.Delforge) Biagioli & Grünanger

Distribution:—Magliano dei Marsi, Mt. Velino, Rosciolo (Pezzetta 2016).

E, # ***Ophrys sphegodes*** Mill. subsp. ***classica*** (Devillers-Tersch. & Devillers) Kreutz—LC

Distribution:—common from hill and submontane to montane belt.

Ophrys sphegodes Mill. subsp. ***tommasinii*** (Vis.) Soó (*O. riojana* auct.)

Distribution:—hill and submontane belt.

__Ophrys tenthredinifera__ Willd.—LC

Distribution:—Aielli (R. Romolini in litt.).

Orchis anthropophora (L.) All.

Distribution:—hill, submontane and montane belt.

Orchis italica Poir.—LC

Distribution:—hill, submontane and montane belt.

Orchis mascula (L.) L. subsp. ***mascula*** (*O. mascula* subsp. *speciosa* (Mutel) Hegi

Distribution:—hill, submontane and montane belt.

Orchis militaris L.

Distribution:—Aielli (Guarrera & Tammaro 1996), Campo Felice (De Santis & Soldati 2019).

Orchis pallens L.

Distribution:—montane belt.

Orchis pauciflora Ten.—LC

Distribution:—common from hill and submontane to montane belt.

Orchis purpurea Huds.

Distribution:—common from hill and submontane to montane belt.

Orchis simia Lam.

Distribution:—Aielli e dintorni (Guarrera & Tammaro 1996).

\# ***Orchis spitzelii*** Saut. ex W.D.J.Koch subsp. ***spitzelii***
Distribution:—from montane to subalpine belt.

\# ***Platanthera bifolia*** (L.) Rich.
Distribution:—montane belt.

\# ***Platanthera chlorantha*** (Custer) Rchb.
Distribution:—hill, submontane and montane belt.

Serapias parviflora Parl.
Distribution:—Aielli (Pezzetta 2016), Colle della Forchetta (Collarmele)! (pers. obs.), between Raiano and Goriano Sicoli (R. Soca in litt.).

Serapias vomeracea (Burm.f.) Briq.
Distribution:—hill and submontane belt.

****Spiranthes spiralis*** (L.) Chevall.
Distribution:—between Capo La Maina and S. Iona! (pers. obs.).

OROBANCHACEAE

Bellardia trixago (L.) All.
Distribution:—hill and submontane belt.

\# ***Euphrasia illyrica*** Wettst.
Distribution:—V.ne di Teve, Fonte Canale, Coste del Cafornia (Lucchese & Lattanzi 1993), Mt. Velino! (APP 34321).

Euphrasia italica Wettst.
Distribution:—montane belt.

Euphrasia liburnica Wettst.
Distribution:—hill, submontane and montane belt.

\# ***Euphrasia officinalis*** L. subsp. ***rostkoviana*** (Hayne) F.Towns.
Distribution:—Velino (Petriccione 1993).

\# ***Euphrasia salisburgensis*** Funck ex Hoppe
Distribution:—from montane to subalpine belt.

Euphrasia stricta D.Wolff ex J.F.Lehm.
Distribution:—montane belt.

Lathraea squamaria L.
Distribution:—Mt. Sirente (Veri & Tammaro 1980), F. Aterno at the Roman bridge near the church of Santa Maria della Vittoria! (APP 73026).

Melampyrum arvense L. subsp. ***arvense***
Distribution:—hill, submontane and montane belt.

\# ***Melampyrum cristatum*** L. subsp. ***cristatum***
Distribution:—hill and submontane belt.

E ***Melampyrum italicum*** (Beauverd) Soó—LC
Distribution:—V.ne di Teve (Lucchese & Lattanzi 1993), Gole di S. Venanzio! (APP 35407).

Melampyrum nemorosum L.
Distribution:—Mt. Sirente (Groves 1880), S. Martino d'Ocre! (APP 67892, 67893, 67894, 67895, 67896, 68203. 68204, 68205).

E, # ***Melampyrum variegatum*** (Porta & Rigo) Huter—LC

Distribution:—V.ne di Teve! (Lucchese & Lattanzi 1993; APP 68374), Gole di Celano! (APP 68193, 68194).

\# ***Odontites luteus*** (L.) Clairv. subsp. ***luteus***

Distribution:—common from hill and submontane to montane belt.

Odontites vernus (Bellardi) Dumort. subsp. ***serotinus*** Corb.

Note:—the specimens collected in the Park (Fossa Raganesca, Lago di Civita and S. Felice d'Ocre) (APP 27457, 34566, 34859, 34860) appear to belong to the subsp. *siculus* (Guss.) P.D.Sell., which has not yet been reported in Abruzzo. Further investigation is needed through comparison with typical populations.

Odontites vernus (Bellardi) Dumort. subsp. ***vernus***

Distribution:—hill and submontane belt.

Orobanche alba Stephan ex Willd.

Distribution:—common in the montane belt.

\# ***Orobanche amethystea*** Thuill.

Distribution:—Velino (Petriccione 1993).

\# ***Orobanche caryophyllacea*** Sm.

Distribution:—common from hill and submontane to montane belt.

Orobanche crenata Forssk.

Distribution:—common in the hill and submontane belt.

****Orobanche flava*** Mart. ex F.W.Schultz

Distribution:—Rocca di Mezzo! (pers. obs.).

Orobanche gracilis Sm.

Distribution:—common from hill and submontane to montane belt.

Orobanche hederae Vaucher ex Duby

Distribution:—common from hill and submontane to montane belt.

Orobanche lutea Baumg.

Distribution:—Gole di S. Venanzio! (Conti et al. 2018b; APP 58392).

****Orobanche minor*** Sm.

Distribution:—presso Gagliano Aterno!, Castelvecchio Subequo! (pers. obs.).

Orobanche reticulata Wallr. subsp. ***reticulata***

Distribution:—montane belt.

Orobanche teucrii Holandre

Distribution:—Mt. Ocre! and Rocca di Cambio! (Conti et al. 2008a; APP 25281, 25282).

Parentucellia latifolia (L.) Caruel

Distribution:—common from hill and submontane to montane belt.

\# ***Pedicularis comosa*** L. subsp. ***comosa***

Distribution:—from montane to subalpine belt.

E, # ***Pedicularis elegans*** Ten.—LC

Distribution:—common from montane to alpine belt.

Pedicularis friderici-augusti Tomm.

Distribution:—between Massa d'Albe and the summit of Velino at 1350 m! (APP 73891), Mt. Costone (Lucchese & Lattanzi 1993). The report by Guarrera & Tammaro (1996) refers to *P. comosa* (herb. Guarrera!).

Pedicularis hoermanniana K.Malý

Distribution:—montane belt.

Pedicularis petiolaris Ten.—NC

Distribution:—Velino (Tenore 1831; Martelli 1904).

Pedicularis tuberosa L.

Distribution:—montane and alpine belt.

Pedicularis verticillata L. subsp. ***verticillata***

Distribution:—common from montane to alpine belt.

****Phelipanche mutelii*** (F.W.Schultz) Pomel

Distribution:—Collarmele! (pers. obs.).

Phelipanche nana (Reut.) Soják

Distribution:—Prati del Lago! (Conti et al. 2008a; APP 67699), Collarmele!, Massa d'Albe! (pers. obs.).

****Phelipanche purpurea*** (Jacq.) Soják

Distribution:—between Rocca di Mezzo and Piani di Pezza! (APP 42310).

Phelipanche ramosa (L.) Pomel

Distribution:—reported for the neighbouring areas of Lucoli (De Santis & Soldati 2019) and Prata d'Ansidonia! (APP 26702) and likely also present in the Park.

Rhinanthus alectorolophus (Scop.) Pollich subsp. ***alectorolophus***

Distribution:—common from hill and submontane to montane belt.

Rhinanthus minor L.

Distribution:—common from hill and submontane to montane belt.

E, # ***Rhinanthus wettsteinii*** (Sterneck) Soó—LC

Distribution:—from montane to subalpine belt.

OXALIDACEAE

N ****Oxalis articulata*** Savigny—CAS

Distribution:—Gagliano Aterno! (pers. obs.).

C ****Oxalis corniculata*** L.

Distribution:—Fontecchio!, Gagliano Aterno! (APP), Rosciolo! (pers. obs.).

N ****Oxalis dillenii*** Jacq.—NAT

Distribution:—Fontecchio!, Santa Maria del Ponte!, Acciano! (APP 74170, 74214, 74221).

PAEONIACEAE

E ***Paeonia officinalis*** L. subsp. ***italica*** N.G.Passal. & Bernardo—LC

Distribution:—Velino (Petriccione 1993 as *P. officinalis* subsp. *villosa*), Mt. Rotondo at the base of the ski resorts! (Guarrera & Tammaro 1996 as *P. officinalis* subsp. *villosa*; Passalacqua & Bernardo 2004 from specimens collected by Anzalone and Montelucci), Campo Felice! (De Santis & Soldati 2011; pers. obs.), Mt. Ocre in V.ne Canavine! (APP 15387).

PAPAVERACEAE

Chelidonium majus L.

Distribution:—common from hill and submontane to montane belt.

Corydalis cava (L.) Schweigg. & Körte subsp. ***cava***

Distribution:—hill, submontane and montane belt.

E, # ***Corydalis densiflora*** C.Presl subsp. ***apennina*** F.Conti, Bartolucci & Uzunov—LC

Distribution:—Campo Felice (De Santis & Soldati 2011), Velino (Petriccione 1993 as *Corydalis solida*) and in various locations in the Lucoli area! (Conti et al. 2019b) but outside the boundaries of the Park.

****Corydalis pumila*** (Host) Rchb.

Distribution:—V.ne di Teve!, Val d'Arano! (pers. obs.).

****Fumaria capreolata*** L. subsp. ***capreolata***

Distribution:—Gole di S. Venanzio! (APP 35698).

Fumaria officinalis L. subsp. ***officinalis***

Distribution:—hill, submontane and montane belt.

Fumaria parviflora Lam.

Distribution:—hill, submontane and montane belt.

Fumaria vaillantii Loisel.

Distribution:—Velino (Tenore 1830; Montelucci 1958), Fonte Canale (Lucchese & Lattanzi 1993).

****Glaucium flavum*** Crantz

Distribution:—between Fossa and the Convento of S. Spirito d'Ocre! (APP 35323).

\# ***Oreomecon alpina*** (L.) Banfi, Bartolucci, J.-M.Tison & Galasso subsp. ***alpina*** (*Papaver alpinum* L. subsp. *alpinum* ; *Papaver alpinum* L. subsp. *ernesti-mayeri* Markgr.; *Papaver julicum* E.Mayer & Merxm.)

Distribution:—from subalpine to alpine belt.

C ***Papaver dubium*** L.

Distribution:—hill, submontane and montane belt.

C, # ***Papaver rhoeas*** L. subsp. ***rhoeas***

Distribution:—common from hill and submontane to montane belt.

A ****Papaver somniferum*** L. subsp. ***somniferum*** —CAS

Distribution:—Ripa! (APP).

\# ***Pseudofumaria alba*** (Mill.) Lidén subsp. ***alba***

Distribution:—Gole di Celano! (Lastoria 1989; Frizzi et al. 1996; Guarrera & Tammaro 1996; Conti 1998; APP 9468, 66988), Valle Majelama (Lucchese & Lattanzi 1993), Velino (Petriccione 1993).

Roemeria argemone (L.) C.Morales, R.Mend. & Romero García (*Papaver argemone* L.)

Distribution:—near Forme! (Conti et al. 2008a; pers. obs.), near Carrito! (APP 33310), Rosciolo! (pers. obs.).

****Roemeria sicula*** (Guss.) Galasso, Banfi, L.Sáez & Bartolucci (*Papaver siculum* Guss.)

Distribution:—S. Benedetto in Perillis! (APP 68177, 68183).

PARNASSIACEAE

Parnassia palustris L. subsp. ***palustris***

Distribution:—montane belt.

PHYLLANTHACEAE

Andrachne telephioides L.

Distribution:—Fontecchio, Passo della Forcella (Tammaro & Pogliani 1977).

PLANTAGINACEAE

N ****Antirrhinum majus*** L.—NAT

Distribution:—Rocca di Mezzo!, Fonteavignone!, Fontecchio!, Castelvecchio Subequo! (APP 27057, 73529, 74207: pers. obs.)

Chaenorhinum minus (L.) Lange subsp. ***minus***

Distribution:—hill, submontane and montane belt.

E ***Cymbalaria glutinosa*** Bigazzi & Raffaelli subsp. ***glutinosa***—LC

Distribution:—Cocorello (Parlatore 1848-96 as *Linaria pilosa*), Alba Fucens (Tammaro & Pace 1994 as *C. muralis* var. *pilosa*).

Cymbalaria muralis G.Gaertn., B.Mey. & Scherb. subsp. ***muralis***

Distribution:—hill, submontane and montane belt.

Cymbalaria muralis G.Gaertn., B.Mey. & Scherb. subsp. ***visianii*** (Jav.) D.A.Webb

Distribution:—hill, submontane and montane belt.

E, # ***Cymbalaria pallida*** (Ten.) Wettst.—LC

Distribution:—uncommon in the high-altitude scree slopes of Velino and Mt. Sirente.

Digitalis ferruginea L.

Distribution:—hill, submontane and montane belt.

E, # ***Digitalis micrantha*** Roth ex Schweigg.—LC

Distribution:—hill, submontane and montane belt.

Globularia bisnagarica L.

Distribution:—hill, submontane and montane belt.

Globularia cordifolia L. subsp. ***bellidifolia*** (Nyman) Wettst.

Distribution:—hill, submontane and montane belt.

****Kickxia elatine*** (L.) Dumort. subsp. ***sieberi*** (Rchb.) Hayek

Distribution:—Goriano Sicoli! (APP 73515).

****Kickxia spuria*** (L.) Dumort.

Distribution:—Castelnuovo presso Forme! (pers. obs.).

Note:—no subspecific level has been assigned due to morphological variability and unclear taxonomic delimitation of the two subspecies reported in Italy.

Linaria alpina (L.) Mill.

Distribution:—from subalpine to alpine belt.

Linaria arvensis (L.) Desf.—D

Distribution:—Massa d'Albe (Parlatore 1848-96), from Rocca di Cambio to L'Aquila (Montelucci 1962), Forca d'Acero (Guarrera & Tammaro 1996).

E, # ***Linaria purpurea*** (L.) Mill.—LC

Distribution:—common in the mountain belt.

Linaria simplex (Willd.) Desf.

Distribution:—hill, submontane and montane belt.

Linaria vulgaris Mill. subsp. ***vulgaris***
Distribution:—common from hill and submontane to montane belt.
****Misopates orontium*** (L.) Raf.
Distribution:—Lago Acquaviva (Molina Aterno)! (APP 27200), Forme! (pers. obs.).
****Plantago afra*** L. subsp. ***afra***
Distribution:—Gole di S. Venanzio! (APP 35724).
Plantago alpina L.—NC
Distribution:—Velino (Tenore 1830), Mt. Sirente (Groves 1880).
\# ***Plantago argentea*** Chaix subsp. ***argentea***
Distribution:—montane belt.
\# ***Plantago atrata*** Hoppe subsp. ***atrata***
Distribution:—montane and alpine belt.
Plantago atrata Hoppe subsp. ***fuscescens*** (Jord.) Pilg.
Distribution:—montane and alpine belt.
Plantago bellardii All. subsp. *bellardii*—NC
Distribution:—Mt. Sirente (Groves 1880).
Plantago coronopus L.
Distribution:—above Secinaro (Guarrera & Tammaro 1996).
\# ***Plantago lanceolata*** L.
Distribution:—hill, submontane and montane belt.
Plantago major L.
Distribution:—hill, submontane and montane belt.
Plantago maritima L. subsp. ***serpentina*** (All.) Arcang.
Distribution:—montane belt.
\# ***Plantago media*** L. subsp. ***media***
Distribution:—montane belt.
\# ***Plantago sempervirens*** Crantz
Distribution:—hill and submontane belt.
Plantago subulata L.
Distribution:—hill, submontane and montane belt.
Veronica acinifolia L.—NC
Distribution:—Mt. Velino (Abbate 1903).
Veronica agrestis L.
Veronica alpina L.—NC
Distribution:—Mt. Velino (Montelucci 1958).
Veronica anagallis-aquatica L. subsp. ***anagallis-aquatica***
Distribution:—montane belt.
\# ***Veronica aphylla*** L. subsp. ***aphylla***
Distribution:—from subalpine to alpine belt.
\# ***Veronica arvensis*** L.
Distribution:—hill, submontane and montane belt.
\# ***Veronica beccabunga*** L. subsp. ***beccabunga***
Distribution:—hill, submontane and montane belt.
Veronica chamaedrys L. subsp. ***chamaedrys***

Distribution:—hill, submontane and montane belt.

\# *Veronica cymbalaria* Bodard subsp. ***cymbalaria***

Distribution:—hill and submontane belt.

Veronica hederifolia L.

Distribution:—hill, submontane and montane belt.

Veronica montana L.

Distribution:—montane belt.

Veronica officinalis L.

Distribution:—hill, submontane and montane belt.

\# ***Veronica orsiniana*** Ten.

Distribution:—hill, submontane and montane belt.

N ***Veronica peregrina*** L.—NAT

Distribution:—Mt. Revecena (Guarrera & Tammaro 1996). The only known location in Abruzzo.

N ***Veronica persica*** Poir.—INV

Distribution:—hill, submontane and montane belt.

****Veronica polita*** Fr.

Distribution:—Rosciolo! (pers. obs.).

Veronica praecox All.

Distribution:—hill, submontane and montane belt.

Veronica prostrata L.

Distribution:—hill, submontane and montane belt.

Veronica scutellata L.

Distribution:—Val d'Arano! (Conti 1998; APP 28595), Campo di Rovere!, Altopiano delle Rocche!, Piano S. di Rufino!, Conche di Ovindoli! (APP 11280, 11281, 28654, 59620, 62182, 66504, 67844, 68103).

\# ***Veronica serpyllifolia*** L.

Distribution:—hill, submontane and montane belt.

Veronica spicata L. subsp. ***fischeri*** (Trávn.) Albach

Nota:—previous reports of *V. barrelieri* H.Schott ex Roem. & Schult. subsp. *barrelieri* (Lucchese & Lattanzi 1993; Guarrera & Tammaro 1996) should to be referred to this taxon. Our specimens have sepals with numerous hairs at the margin, which are flexible and not short, sparse and stiff as in *V. barrelieri* . The hairiness on the faces of the sepals seems variable.

Veronica urticifolia Jacq.

Distribution:—Mt. Sirente (Veri & Tammaro 1980).

Veronica verna L. subsp. ***verna***

Distribution:—Velino (Tenore 1830; Grande 1925; Montelucci 1958), territory of Lucoli (De Santis & Soldati 2019), Campo Felice! (APP 30121, 30122), Terranera! (pers. obs.).

PLUMBAGINACEAE

E, # ***Armeria gracilis*** Ten.—DD

Note:—common from montane to subalpine belt. The records of *A. majellensis* Boiss. should be here referred (Tiburtini et al., in preparation).

E *Goniolimon tataricum* (L.) Boiss. subsp. *italicum* (Tammaro, Pignatti & Frizzi) Buzurović—EN

Distribution:—note for locations near the Park territory: Fossa del Campanaro!, Colle di Monticchio!, Fossa Raganesca!, Mt. Ocre, above Navelli on Colle S. Eugenia! (Tammaro et al. 1982; Frattaroli 1988; Conti et al. 2008b; pers. obs.), Mt. Cavalletto! (pers. obs.).

Plumbago europaea L.
Distribution:—hill and submontane belt.

POACEAE

Achnatherum bromoides (L.) P.Beauv.
Distribution:—hill and submontane belt.

****Achnatherum virescens*** (Trin.) Banfi, Galasso & Bartolucci
Distribution:—Gole di S. Venanzio! (APP 65137, 66894).

Agrostis capillaris L.
Distribution:—montane belt.

Agrostis castellana Boiss. & Reut.
Distribution:—Campo Felice (Ciaschetti et al. 2024).

Agrostis stolonifera L. subsp. ***stolonifera***
Distribution:—montane belt.

Alopecurus aequalis Sobol.
Distribution:—montane belt.

Alopecurus bulbosus Gouan
Distribution:—Prati del Sirente (Guarrera & Tammaro 1996), Laghetto di Ovindoli! (APP 67764).

Alopecurus myosuroides Huds.
Distribution:—hill and submontane belt.

Alopecurus pratensis L. subsp. ***pratensis***
Distribution:—hill, submontane and montane belt.

Alopecurus rendlei Eig
Distribution:—hill, submontane and montane belt.

Anisantha diandra (Roth) Tutin ex Tzvelev
Distribution:—hill and submontane belt.

Anisantha madritensis (L.) Nevski
Distribution:—hill and submontane belt.

Anisantha rigida (Roth) Hyl.
Distribution:—hill and submontane belt.

Anisantha rubens (L.) Nevski
Distribution:—above Secinaro (Guarrera & Tammaro 1996).

Anisantha sterilis (L.) Nevski
Distribution:—hill, submontane and montane belt.

Anisantha tectorum (L.) Nevski
Distribution:—montane belt.

Anthoxanthum nipponicum Honda
Distribution:—Campo Felice (De Santis & Soldati 2011).

Anthoxanthum odoratum L.
Distribution:—common from hill and submontane to subalpine belt.
Apera interrupta (L.) P.Beauv.—NC
Distribution:—Mt. Velino (Bertoloni 1833-54 as *Agrostis interrupta*).
Arrhenatherum elatius (L.) P.Beauv. ex J.Presl & C.Presl subsp. *bulbosum* (Willd.) Schübl. & G.Martens—NC
Distribution:—Mt. Velino (Tenore 1831 as *Avena bulbosa* Pers.).
Arrhenatherum elatius (L.) P.Beauv. ex J.Presl & C.Presl subsp. ***elatius***
Distribution:—hill, submontane and montane belt.
A ***Arundo donax*** L.—INV
Distribution:—hill and submontane belt.
Avena barbata Pott ex Link
Distribution:—hill and submontane belt.
A ***Avena fatua*** L.—NAT
Distribution:—hill and submontane belt.
Avenella flexuosa (L.) Drejer subsp. ***flexuosa***
Distribution:—hill, submontane and montane belt.
Avenula pubescens (Huds.) Dumort.
Distribution:—montane belt.
Bellardiochloa variegata (Lam.) Kerguélen
Distribution:—montane belt.
Bothriochloa ischaemum (L.) Keng
Distribution:—hill and submontane belt.
Brachypodium distachyon (L.) P.Beauv.
Distribution:—hill and submontane belt.
E, # ***Brachypodium genuense*** (DC.) Roem. & Schult.—LC
Distribution:—common in the subalpine belt.
Brachypodium rupestre (Host) Roem. & Schult.
Distribution:—common from hill and submontane to montane belt.
Brachypodium sylvaticum (Huds.) P.Beauv. subsp. ***sylvaticum***
Distribution:—common from hill and submontane to montane belt.
Briza media L.
Distribution:—montane belt.
Bromopsis erecta (Huds.) Fourr.
Distribution:—common from hill and submontane to montane belt.
N ****Bromopsis inermis*** (Leyss.) Holub subsp. ***inermis***—NAT
Distribution:—near Rocca di Mezzo! (APP 73469).
Bromopsis ramosa (Huds.) Holub subsp. ***ramosa***
Distribution:—hill, submontane and montane belt.
Bromus arvensis L.
Distribution:—hill, submontane and montane belt.
Bromus commutatus Schrad. subsp. ***commutatus***
Distribution:—Mt. Velino (Lucchese & Lattanzi 1993).
Bromus hordeaceus L. subsp. ***hordeaceus***
Distribution:—common from hill and submontane to montane belt.

Bromus lanceolatus Roth
Distribution:—hill and submontane belt.
Bromus racemosus L. subsp. ***racemosus***
Distribution:—hill and submontane belt.
Bromus squarrosus L.
Distribution:—hill and submontane belt.
Calamagrostis varia (Schrad.) Host
Distribution:—Valle di Teve and Coste del Cafornia (Lucchese & Lattanzi 1993).
Catabrosa aquatica (L.) P.Beauv.
Distribution:—Campo di Rovere! (Ciaschetti et al. 2005; pers. obs.).
Catapodium rigidum (L.) C.E.Hubb. subsp. ***rigidum***
Distribution:—common in the hill and submontane belt.
Ciliochloa effusa (Link) Röser, Tkach & Rasti (*Cynosurus effusus* Link)—LC
Distribution:—montane belt.
Cleistogenes serotina (L.) Keng subsp. ***serotina***
Distribution:—Vittorito (Corbetta et al. 2004).
Cynodon dactylon (L.) Pers.
Distribution:—common from hill and submontane to montane belt.
Cynosurus cristatus L.
Distribution:—common from hill and submontane to montane belt.
Dactylis glomerata L. subsp. ***glomerata***
Distribution:—common from hill and submontane to subalpine belt.
Dactylis glomerata L. subsp. ***hispanica*** (Roth) Nyman
Distribution:—hill, submontane and montane belt.
Danthonia decumbens (L.) DC. subsp. ***decumbens***
Distribution:—montane belt.
Dasypyrum villosum (L.) P.Candargy
Distribution:—hill and submontane belt.
Deschampsia cespitosa (L.) P.Beauv. subsp. ***cespitosa***
Distribution:—montane belt.
****Digitaria sanguinalis*** (L.) Scop.
Distribution:—Lago di Civita!, Gagliano Aterno! (APP 34828, 74229).
Drymochloa sylvatica (Pollich) Holub
Distribution:—Mt. Sirente (Veri & Tammaro 1980), loc. Neviera! (APP 73385).
Echinaria capitata (L.) Desf.
Distribution:—hill and submontane belt.
Echinochloa crus-galli (L.) P.Beauv. subsp. ***crus-galli***
Distribution:—hill and submontane belt.
Elymus caninus (L.) L.
Distribution:—hill, submontane and montane belt.
Elymus repens (L.) Gould subsp. ***repens***
Distribution:—hill, submontane and montane belt.
Eragrostis cilianensis (All.) Vignolo ex Janch.
Distribution:—hill, submontane and montane belt.
****Eragrostis minor*** Host

Distribution:—near Secinaro!, staz. di Fagnano-Campana!, Santa Maria del Monte!, Gagliano Aterno!, Acciano! (APP 74172, 74197, 74216, 74228, 74233).

Eragrostis pilosa (L.) P.Beauv. subsp. ***pilosa***

Distribution:—hill, submontane and montane belt.

Falona echinata (L.) Dumort. (*Cynosurus echinatus* L.)

Distribution:—hill, submontane and montane belt.

E, # ***Festuca alfrediana*** Foggi & Signorini subsp. ***ferrariniana*** Foggi, Parolo & Gr.Rossi—LC

Distribution:—from montane to subalpine belt.

Festuca ambigua Le Gall

Distribution:—hill and submontane belt.

****Festuca bosniaca*** Kumm. & Sendtn. subsp. ***bosniaca***

Distribution:—from rif. Sebastiani to Cimata di Pezza! (APP 73296).

E, # ***Festuca centroapenninica*** (Markgr.-Dann.) Foggi, F.Conti & Pignatti—LC

Distribution:—near Terranera (Guarrera & Tammaro 1996), Piani di Pezza (Ciaschetti et al. 2006).

Festuca cyrnea (Litard. & St.-Yves) Signorini, Foggi & Nardi—DD

Distribution:—Piani di Pezza (Ciaschetti et al. 2006).

Festuca heterophylla Lam.

Distribution:—hill, submontane and montane belt.

E ***Festuca imperatrix*** Catonica—LC

Distribution:—Campo Felice (Catonica 2001).

Festuca inops De Not.—LC

Distribution:—hill, submontane and montane belt.

Festuca laevigata Gaudin

Distribution:—montane and alpine belt.

Festuca marginata (Hack.) K.Richt. subsp. ***marginata***—LC

Distribution:—hill, submontane and montane belt.

Festuca maritima L.

Distribution:—Serra di Celano near the village! (Conti et al. 2006; APP 10549).

Festuca myuros L. subsp. ***myuros***

Distribution:—reported for the neighbouring territory of Lucoli (De Santis & Soldati 2019) and likely also present in the Park.

Festuca rubra L. subsp. ***commutata*** (Gaudin) Markgr.-Dann.

Distribution:—montane belt.

****Festuca rupicola*** Heuff.

Distribution:—Prati del Sirente! (APP 59929, 67715, 67716). Although currently assigned to this species, ongoing studies suggest it probably represents a taxon new to science.

Festuca trachyphylla (Hack.) R.P.Murray

Distribution:—montane belt.

Festuca valesiaca Schleich. ex Gaudin

Distribution:—Velino (Tenore 1831; Filibeck et al. 2020).

E, # ***Festuca violacea*** Ser. ex Gaudin subsp. ***italica*** Foggi, Gr.Rossi &

Distribution:—spreading from the mountain belt to the peaks.

Glyceria fluitans (L.) R.Br.
Distribution:—Colle Ciaccio! (APP 72914, 72915), Piana di Ovindoli! (APP 66543).

Glyceria notata Chevall.
Distribution:—montane belt.

E, # ***Helictochloa praetutiana*** (Parl. ex Arcang.) Bartolucci, F.Conti, Peruzzi & Banfi subsp. ***praetutiana*** —LC
Distribution:—widespread in mountain and subalpine grasslands.

Helictochloa pratensis (L.) Romero Zarco subsp. *pratensis* —NC
Distribution:—Velino (Martelli 1904).

Helictochloa versicolor (Vill.) Romero Zarco subsp. *versicolor* —NC
Distribution:—Mt. Velino (Tenore 1831 sub *Avena versicolor*).

Holcus lanatus L. subsp. ***lanatus***
Distribution:—hill, submontane and montane belt.

Hordelymus europaeus (L.) Harz
Distribution:—hill, submontane and montane belt.

Hordeum murinum L. subsp. ***leporinum*** (Link) Arcang.
Distribution:—hill, submontane and montane belt.

Hordeum murinum L. subsp. ***murinum***
Distribution:—hill and submontane belt.

Hordeum secalinum Schreb.
Distribution:—Piana di Ovindoli! (APP 68474).

Koeleria macrantha (Ledeb.) Schult. subsp. *macrantha* —NC
Distribution:—Mt. Velino (Tenore 1831 sub *Koeleria cristata* , Bertoloni 1833-54 sub *Aira cristata*).

Koeleria splendens C.Presl—LC
Distribution:—montane belt.

Leucopoa dimorpha (Guss.) H.Scholz & Foggi
Distribution:—from montane to subalpine belt.

Lolium arundinaceum (Schreb.) Darbysh. subsp. ***arundinaceum***
Distribution:—montane belt.

Lolium interruptum (Desf.) Banfi, Galasso, Foggi, Kopecký & Ardenghi
Distribution:—Valle di Sevice (Lucchese & Lattanzi 1993), Piana di Ovindoli! (APP 66588, 66589).

Lolium perenne L.
Distribution:—hill, submontane and montane belt.

Lolium pratense (Huds.) Darbysh.
Distribution:—Mt. Velino (Veri & Tammaro 1980), Molina Aterno (Guarrera & Tammaro 1996).

Melica ciliata L.
Distribution:—hill, submontane and montane belt.

Melica nutans L.
Distribution:—Velino (Petriccione 1993), Valle Maiori!, Costa delle Tavole! (APP 15564, 67626).

Melica transsilvanica Schur

Distribution:—Valle di Sevice and V.ne di Teve (Lucchese & Lattanzi 1993), Pié della Selva! (APP 28302).

Melica uniflora Retz.

Distribution:—hill, submontane and montane belt.

Milium effusum L.

Distribution:—hill, submontane and montane belt.

Milium vernale M.Bieb.

Distribution:—Cimata di Pezza (Lucchese & Lattanzi 1993), between Rocca di Mezzo and Passo della Forcella (Guarrera & Tammaro 1996).

Molinia caerulea (L.) Moench

Distribution:—Mt. Revecena (Guarrera & Tammaro 1996), Altopiano delle Rocche!, Piana di Ovindoli! (APP 59621, 60343, 66545, 66616, 72906).

Nardus stricta L.

Distribution:—from montane to subalpine belt.

Oloptum miliaceum (L.) Röser & H.R.Hamas (*Piptatherum miliaceum* (L.) Coss.)

Distribution:—V.ne di Teve (Lucchese & Lattanzi 1993).

****Oloptum thomasii*** (Duby) Banfi & Galasso

Distribution:—Gole di S. Venanzio! (APP 35710).

N ****Panicum capillare*** L.—NAT

Distribution:—Molina Aterno!, Fossa! (APP 27449, 27450, 27451, 28377, 28378).

Patzkea paniculata (L.) G.H.Loos subsp. ***paniculata***

Distribution:—from montane to subalpine belt.

****Phalaris arundinacea*** L. subsp. ***arundinacea***

Distribution:—Molina Aterno at Lago Acquaviva!, F. Aterno near Fontecchio! and near Ponte di Campana! (APP 27191, 68278, 73557).

Phalaris coerulescens Desf. —NC

Distribution:—Mt. Sirente (Groves 1880).

Phleum hirsutum Honck.

Distribution:—hill, submontane and montane belt.

Phleum nodosum L.

Distribution:—Piani di Pezza (Ciaschetti et al. 2006), Campo di Rovere (Ciaschetti et al. 2024), Conche di Ovindoli! (APP 66597).

Phleum paniculatum Huds.

Distribution:—Prati del Sirente, Rovere, Passo della Forcella, Ovindoli (Guarrera & Tammaro 1996).

Phleum pratense L.

Distribution:—montane belt.

Phleum rhaeticum (Humphries) Rauschert

Distribution:—from montane to subalpine belt.

Phragmites australis (Cav.) Trin. ex Steud.

Distribution:—hill and submontane belt.

Poa alpina L.

Distribution:—from montane to subalpine belt.

Poa annua L.
Distribution:—hill, submontane and montane belt.

\# ***Poa bulbosa*** L.
Distribution:—hill, submontane and montane belt.

Poa compressa L.
Distribution:—hill, submontane and montane belt.

\# ***Poa molinerii*** Balb.
Distribution:—from montane to subalpine belt.

\# ***Poa nemoralis*** L.
Distribution:—hill, submontane and montane belt.

Poa pratensis L. subsp. ***pratensis***
Distribution:—hill, submontane and montane belt.

\# ***Poa sylvicola*** Guss.
Distribution:—Valle di Sevice (Lucchese & Lattanzi 1993).

Poa trivialis L.
Distribution:—hill, submontane and montane belt.

****Polypogon monspeliensis*** (L.) Desf.
Distribution:—conche di Ovindoli (pers. obs.). The species is usually found in coastal environments or at lower altitudes. We found a small group with atypical characteristics (lower spikelets measuring 1.7 mm and awns approximately 5 mm shorter).

****Sclerochloa dura*** (L.) P.Beauv.
Distribution:—Piana di S. Nicola! (pers. obs.).

A ***Secale cereale*** L. subsp. ***cereale***—CAS
Distribution:—Colle Orso (Viegi et al. 1990), Colle Ciaccio! (APP 72924,

Sesleria autumnalis (Scop.) F.W.Schultz—LC
Distribution:—V.ne di Teve (Lucchese & Lattanzi 1993).

\# ***Sesleria juncifolia*** Suffren—LC
Distribution:—common form hill and submontane belt to subalpine belt.

E, # ***Sesleria nitida*** Ten.—LC
Distribution:—common in the montane belt.

Sesleria uliginosa Opiz—NT
Distribution:—montane belt.

A ***Setaria italica*** (L.) P.Beauv. subsp. ***italica***—CAS
Distribution:—hill and submontane belt.

C, # ***Setaria italica*** (L.) P.Beauv. subsp. ***viridis*** (L.) Thell.
Distribution:—Valle Majelama (Lucchese & Lattanzi 1993), Fossa along F. Aterno!, Acciano!, Secinaro! (APP 27453, 27454, 74181, 74199).

N ****Setaria parviflora*** (Poir.) Kerguélen—CAS
Distribution:—Acciano! (APP 74182).

Setaria pumila (Poir.) Roem. & Schult.
Distribution:—Aielli and surroundings (Guarrera & Tammaro 1996).

****Setaria verticillata*** (L.) P.Beauv.
Distribution:—Acciano! (pers. obs.).

N ****Sporobolus indicus*** (L.) R.Br.—CAS

Distribution:—Castello d'Ocre (Banfi in verb.).

\# ***Stipa capillata*** L.

Distribution:—hill and submontane belt.

E ***Stipa dasyvaginata*** Martinovský subsp. ***apenninicola*** Martinovský & Moraldo—LC

Distribution:—common in the mountain pastures.

E *Trisetum bertolonii* Jonsell—D, LC

Distribution:—Gole di S. Venanzio (Pirone et al. 1997). The species is typical of mountainous and alpine belts. Its presence in the Gole di S.Venanzio is doubtful and probably to be excluded.

Trisetum flavescens (L.) P.Beauv. subsp. ***flavescens***

Distribution:—montane belt.

A ***Triticum aestivum*** L. subsp. ***aestivum***—CAS

Distribution:—hill, submontane and montane belt.

A ***Triticum ×requienii*** Ces., Pass. & Gibelli nothosubsp. ***requienii***—CAS

Distribution:—Rocca di Mezzo! (Conti et al. in press; APP 73401).

\# ***Triticum vagans*** (Jord. & Fourr.) Greuter

Distribution:—common from hill and submontane to montane belt.

Ventenata dubia (Leers) Coss.—NC

Distribution:—Velino (Tenore 1831).

POLYGALACEAE

E, # ***Polygala alpestris*** Rchb. subsp. ***angelisii*** (Ten.) Nyman—LC

Note:—identification at the infraspecific level is unclear and requires further

****Polygala amarella*** Crantz

Distribution:—Campo di Rovere!, Conche di Ovindoli!, Laghetto di Ovindoli! (APP 34790, 66477, 67797).

****Polygala comosa*** Schkuhr

Distribution:—Campo di Rovere!, Conche di Ovindoli!, Piana di Ovindoli!, Piani di Pezza! (APP 64921, 64926, 66475, 66738, 68241).

E, # ***Polygala flavescens*** DC. subsp. ***flavescens***—DD

Distribution:—Mt. Velino (Petriccione 1993, Conti 1998).

\# ***Polygala major*** Jacq.

Distribution:—common from hill and submontane to alpine belt.

****Polygala monspeliaca*** L.

Distribution:—Collarmele!, near Carrito! (pers. obs.).

\# ***Polygala nicaeensis*** Risso ex W.D.J.Koch subsp. ***nicaeensis*** (*Polygala nicaeensis* subsp. *mediterranea* Chodat)

Distribution:—common from hill and submontane to montane belt.

E ***Polygala nicaeensis*** Risso ex W.D.J.Koch subsp. ***peninsularis*** Arrigoni

Distribution:—pastures at the foot of the hills.

Polygala serpyllifolia Hosé—D

Distribution:—Velino (Abbate 1903 as *Polygala serpyllaceam* Weich.).

\# ***Polygala vulgaris*** L. subsp. ***vulgaris***

Distribution:—hill, submontane and montane belt.

POLYGONACEAE

Bistorta officinalis Delarbre

Distribution:—montane belt.

Bistorta vivipara (L.) Delarbre

Distribution:—Mt. Sirente (Guarrera & Tammaro 1996).

N ****Fallopia baldschuanica*** (Regel) Holub—INV

Distribution:—Pié della Selva (Fontecchio)! (APP 28306, 28307).

Fallopia convolvulus (L.) Á.Löve

Distribution:—hill, submontane and montane belt.

Fallopia dumetorum (L.) Holub

Distribution:—hill, submontane and montane belt.

Persicaria amphibia (L.) Delarbre

Distribution:—montane belt.

Persicaria decipiens (R.Br.) K.L.Wilson

Distribution:—Altopiano delle Rocche, Val d'Arano (Ciaschetti et al. 2005).

Persicaria hydropiper (L.) Delarbre

Distribution:—Gole di Celano (Frizzi et al. 1996; Guarrera & Tammaro 1996).

****Persicaria lapathifolia*** (L.) Delarbre subsp. ***lapathifolia***

Distribution:—F. Aterno near Fossa!, Tione degli Abruzzi!, Molina Aterno!, Lago di Civita!, Laghetto di Goriano Valli! (APP 26766, 28223, 28259, 28260, 28384, 28385, 34868, 74242, 74245), Rocca di Mezzo! (pers. obs.).

****Polygonum arenastrum*** Boreau

Distribution:—Recolle (Gagliano Aterno)!, Campo Felice at the base of the ski lifts! (APP 28330, 72950).

Polygonum aviculare L. subsp. ***aviculare***

Polygonum bellardii All.—NC

Distribution:—Lago di Molina and Massa d'Albe (Raffaelli 1979 from specimens by Groves and Levier in FI).

Polygonum rurivagum Jord. ex Boreau

Distribution:—hill and submontane belt.

Rumex acetosa L. subsp. ***acetosa***

Distribution:—hill, submontane and montane belt.

Rumex acetosella L. subsp. ***pyrenaicus*** (Pourr. ex Lapeyr.) Akeroyd

Note:—reports of *R. acetosella* should be referred to this taxon.

Rumex alpinus L.

Distribution:—Piani di Pezza (Avena & Blasi 1975)

Rumex arifolius All.

Distribution:—montane belt.

Rumex conglomeratus Murray

Distribution:—Aielli and surroundings (Guarrera & Tammaro 1996).

Rumex crispus L.

Distribution:—hill, submontane and montane belt.

Rumex intermedius DC.—NC

Distribution:—Velino (Tenore 1830, 1831).

\# ***Rumex nebroides*** Campd.
Distribution:—from montane to subalpine belt.
Rumex obtusifolius L. subsp. ***obtusifolius***
Distribution:—Mt. Sirente (Veri & Tammaro 1980).

A ***Rumex patientia*** L. subsp. ***patientia***—NAT
Distribution:—montane belt.

\# ***Rumex sanguineus*** L.
Distribution:—hill, submontane and montane belt.

\# ***Rumex scutatus*** L. subsp. ***scutatus***
Distribution:—hill, submontane and montane belt.
Rumex thyrsiflorus Fingerh.
Distribution:—laghetto di Val d'Arano! (Conti et al. 2025a; APP 28596), Colle Ciaccio! (APP 72922, 72923).

PORTULACACEAE

C ***Portulaca oleracea*** L.
Distribution:—hill and submontane belt.

POTAMOGETONACEAE

Potamogeton crispus L.
Distribution:—common in the hill and submontane belt.
Potamogeton lucens L.—NC
Distribution:—"ad lacum Molina" (Lastrucci et al. 2010 from a specimen collected by Groves).
Potamogeton natans L.
Distribution:—common from hill and submontane to montane belt.
Potamogeton perfoliatus L.—NC
Distribution:—Mt. Sirente along F.Aterno (Groves 1880).
****Potamogeton nodosus*** Poir.
Distribution:—Raiano at Sorgente La Solfa! (pers. obs.).
****Potamogeton trichoides*** Cham. & Schltdl.
Distribution:—Campo di Rovere! (APP 67718, 67726).

PRIMULACEAE

Androsace maxima L.
Distribution:—Velino (Fiori 1923-29), Forme! (pers. obs.).

\# ***Androsace villosa*** L. subsp. ***villosa***
Distribution:—from montane to subalpine belt.

E, # ***Androsace vitaliana*** (L.) Lapeyr. subsp. ***praetutiana*** (Buser ex Sünd.) Kress—LC
Distribution:—ridges, summit environments of Velino and Mt. Sirente.

\# ***Cyclamen hederifolium*** Aiton subsp. ***hederifolium***
Distribution:—hill, submontane and montane belt.
Cyclamen repandum Sm. subsp. ***repandum***
Distribution:—hill and submontane belt.

Lysimachia arvensis (L.) U.Manns & Anderb.
Distribution:—hill, submontane and montane belt.
Lysimachia foemina (Mill.) U.Manns & Anderb.
Distribution:—hill, submontane and montane belt.
Lysimachia linum-stellatum L.
Distribution:—hill, submontane and montane belt.
Lysimachia vulgaris L.
Distribution:—Mt. Sirente along F. Aterno! (Groves 1880), Altopiano delle Rocche (Ciaschetti et al. 2005), Molina Aterno at Lago Acquaviva! (APP 27195), Val d'Arano! (pers. obs.).

Primula auricula L.
Distribution:—montane and alpine belt.

Primula veris L. subsp. ***columnae*** (Ten.) Maire & Petitm.
Distribution:—hill, submontane and montane belt.

Primula vulgaris Huds. subsp. ***vulgaris***
Distribution:—common from hill and submontane to montane belt.

RANUNCULACEAE

Aconitum lycoctonum L. emend. Koelle
Distribution:—montane belt.

Actaea spicata L.
Distribution:—hill, submontane and montane belt.

****Adonis aestivalis*** L. subsp. ***aestivalis***
Distribution:—Laghetto di Collepietro! (APP 27338), Prati del Lago! (APP 68073), Campo dell'Olmo (Cerchio)! (APP 68379, 68380).
Adonis annua L.
Distribution:—hill, submontane and montane belt.

E, # ***Adonis distorta*** Ten.—EN
Distribution:—Velino! (Gussone 1826; Tenore 1831; Bertoloni 1833-54; Montelucci 1958, 1971; Avena & Blasi 1980; Frattaroli & Frizzi 1988; Petriccione 1993; Pace & Catonica 1998; APP 67606), Mt. Sirente! (Groves 1880; Gruppo di Lavoro per la Conservazione della Natura 1971; Veri & Tammaro 1980; Frattaroli & Frizzi 1988; Guarrera & Tammaro 1996; Pace & Catonica 1998). Species included in All. II and IV of the Habitats Directive 92/43/EEC.

Adonis flammea Jacq. subsp. ***cortiana*** C.H.Steinb.
Distribution:—hill and submontane belt.
Adonis flammea Jacq. subsp. ***flammea***
Distribution:—hill and submontane belt.
****Adonis vernalis*** L.—EN
Distribution:— Mt. Offermo, valley near S. Erasmo! (APP 70712). The discovery was made by hikers and the news was published in several local newspapers.

Anemone apennina L.
Distribution:—common from hill and submontane to montane belt.

Anemone hortensis L. subsp. ***hortensis***

Distribution:—reported for the neighbouring territory of Lucoli (De Santis & Soldati 2019) and likely also present in the Park.

Anemonoides nemorosa (L.) Holub

Distribution:—montane belt.

Anemonoides ranunculoides (L.) Holub

Distribution:—common from hill and submontane to montane belt.

****Aquilegia** cv.*

Distribution:—S. Panfilo d'Ocre!, Rocca di Mezzo! (APP 73528; pers. obs.).

Aquilegia dumeticola Jord.

Distribution:—hill, submontane and montane belt.

Ceratocephala falcata (L.) Cramer

Distribution:—hill, submontane and montane belt.

Clematis flammula L.

Distribution:—hill and submontane belt.

Clematis vitalba L.

Distribution:—hill, submontane and montane belt.

Delphinium ajacis L.

Distribution:—V.ne di Sevice, V.ne di Teve (Lucchese & Lattanzi 1993), Massa d'Albe!, Forme! (pers. obs.).

Delphinium consolida L. subsp. ***consolida***

Distribution:—hill, submontane and montane belt.

Delphinium consolida L. subsp. ***paniculatum*** (Host) N.Busch

Distribution:—hill and submontane belt.

Delphinium fissum Waldst. & Kit. subsp. ***fissum***

Distribution:—montane belt.

Delphinium halteratum Sm. subsp. ***halteratum***

Distribution:—hill and submontane belt.

Eranthis hyemalis (L.) Salisb.

Distribution:—hill, submontane and montane belt.

Ficaria verna Huds. subsp. ***calthifolia*** (Rchb.) Nyman

Distribution:—montane belt.

Ficaria verna Huds. subsp. ***fertilis*** (Lawalrée ex Laegaard) Stace

Distribution:—hill and submontane belt.

Ficaria verna Huds. subsp. ***ficariiformis*** (F.W.Schultz) B.Walln.

Distribution:—hill and submontane belt.

Helleborus foetidus L. subsp. ***foetidus***

Distribution:—common from hill and submontane to montane belt.

E ***Helleborus viridis*** L. subsp. ***abruzzicus*** (M.Thomsen, McLewin & B.Mathew) Bartolucci, F.Conti & Peruzzi—LC

Distribution:—Mt. Corona from a specimen kept at FI s. coll. (Zanotti & Cristofolini 1994 as H. multifidus), Valle Majori!, Lago di Tempra!, Mt. Briccialone!, below Forca Caruso! (APP 59869, 59870, 59871, 65105, 65106, 65107, 67189, 67412, 72944).

E ***Helleborus viridis*** L. subsp. ***bocconei*** (Ten.) Peruzzi—LC

Distribution:—Velino (Montelucci 1958), Mt. Sirente in Valle Lupara, above Piano di Canale! (Guarrera & Tammaro 1996).

\# ***Hepatica nobilis*** Schreb.
Distribution:—common from hill and submontane to montane belt.

Myosurus minimus L.
Distribution:—Campo Felice along Lake Camardosa shores! (Conti et al. 2002), Campo di Rovere! (Conti et al. 2008a).

Nigella damascena L.
Distribution:—hill, submontane and montane belt.

\# ***Pulsatilla alpina*** (L.) Delarbre subsp. ***millefoliata*** (Bertol.) D.M.Moser
Distribution:—common from montane to alpine belt.

Ranunculus acris L. subsp. ***acris***

Ranunculus acris L. subsp. *friesianus* (Jord.) Syme—D
Distribution:—Aielli, Ovindoli (Guarrera & Tammaro 1996). Its presence in the locations indicated (the only ones for the region) is probably erroneous and should be removed. The samples observed in the Guarrera herbarium are incomplete and do not allow a correct identification.

E, # ***Ranunculus apenninus*** (Chiov.) Pignatti—LC
Distribution:—in the grassy peaks of Velino and Mt. Sirente.

Ranunculus arvensis L.
Distribution:—hill, submontane and montane belt.

\# ***Ranunculus brevifolius*** Ten.
Distribution:—from subalpine to alpine belt.

\# ***Ranunculus breyninus*** Crantz
Distribution:—montane and alpine belt.

\# ***Ranunculus bulbosus*** L.
Distribution:—hill, submontane and montane belt.

****Ranunculus flammula*** L.
Distribution:—Campo di Rovere!, Val d'Arano!, Prato della Madonna!, Colle Ciaccio! (APP 28655, 34791, 34816, 34817, 59879, 60344, 60345, 67721, 68399, 72907).

\# ***Ranunculus gramineus*** L.
Distribution:—montane belt.

\# ***Ranunculus illyricus*** L.
Distribution:—hill, submontane and montane belt.

\# ***Ranunculus lanuginosus*** L.
Distribution:—hill, submontane and montane belt.

Ranunculus lateriflorus DC.
Distribution:—Piano di S. Nicola below Forca Caruso! (Conti et al. 2008a; APP 40588), Campo di Rovere! (Conti et al. 2023a; APP 67717).

Ranunculus lingua L.—NC
Distribution:—Mt. Sirente along the F. Aterno (Groves 1880).

E ***Ranunculus magellensis*** Ten.—DD
Distribution:—long-snow-covered areas of Mt. Sirente in numerous locations, and Mt. Costone (Lucchese & Lattanzi 1993).

E **Ranunculus marsicus** Guss. & Ten.—DD
Distribution:—Altopiano delle Rocche! (Conti 1998; APP 28633, 51555, 51556, 51557, 51558, 51559, 51560, 51561, 51562, 51563, 54632, 68037), Campo Felice! (De Sanctis & Conti 2000; APP 30126, 30131, 30132, 30149, 51574, 51575), Val d'Arano!, Conche di Ovindoli! (APP 28607, 66571).

Ranunculus millefoliatus Vahl
Distribution:—hill, submontane and montane belt.

Ranunculus monspeliacus L. subsp. **monspeliacus**
Distribution:—montane belt.

Ranunculus montanus Willd. aggr.
Distribution:—from montane to subalpine belt.

Ranunculus neapolitanus Ten.
Distribution:—hill and submontane belt.

Ranunculus repens L.
Distribution:—montane belt.

Ranunculus sardous Crantz
Distribution:—hill and submontane belt.

E ***Ranunculus thomasii*** Ten.—LC
Distribution:—Altopiano delle Rocche!, Campo Felice!, Campo di Rovere!, laghetto di Ovindoli!, Piano Canale! (APP 26955, 34807, 34812, 34815, 67740, 67791, 67825, 68105, 68122, 68288, 68303)

Ranunculus trichophyllus Chaix
Distribution:—hill, submontane and montane belt.

Ranunculus tuberosus Lapeyr. (*R. nemorosus* DC.)
Distribution:—hill, submontane and montane belt.

Ranunculus velutinus Ten.
Distribution:—hill, submontane and montane belt.

Thalictrum aquilegiifolium L. subsp. **aquilegiifolium**
Distribution:—montane belt.

Thalictrum flavum L.
Distribution:—Mt. Sirente along F. Aterno (Groves 1880), Prati del Sirente (Guarrera & Tammaro 1996), V.ne di Teve (Petriccione 1993 as *T. exaltatum* subsp. *mediterraneum*), Monticchio near the cemetery! (APP 56637, 56638).

Thalictrum foetidum L. subsp. **foetidum**
Distribution:—Mt. Sirente, Valle Lupara (Groves, 1880; Guarrera & Tammaro 1996; Hand 2001 from a specimen collected by Groves), Valle Pretosa!, Valle Inserrata! (APP 27643, 27658, 28549).

Thalictrum lucidum L.
Distribution:—hill and submontane belt.

Thalictrum minus L. subsp. **minus**
Distribution:—hill, submontane and montane belt.

Thalictrum simplex L. subsp. **simplex**
Distribution:—hill, submontane and montane belt.

Trollius europaeus L.
Distribution:—montane belt.

RESEDACEAE

\# *Reseda lutea* L. subsp. *lutea*

Distribution:—hill, submontane and montane belt.

Reseda luteola L.

Distribution:—hill, submontane and montane belt.

Reseda phyteuma L. subsp. *phyteuma*

Distribution:—hill and submontane belt.

RHAMNACEAE

Atadinus alpinus (L.) Raf.

Distribution:—montane belt.

\# *Atadinus fallax* (Boiss.) Hauenschild

Distribution:—montane belt.

\# *Atadinus pumilus* (Turra) Hauenschild subsp. *pumilus*

Distribution:—hill, submontane and montane belt.

**Paliurus spina-christi* Mill.

Distribution:—between Collarmele and Pescina!, Alba Fucens!, Fontecchio!, Gole di S. Venanzio! (pers. obs.).

Rhamnus alaternus L. subsp. *alaternus*

Distribution:—Gole di S. Venanzio (Pirone & Cutini 2002).

Rhamnus cathartica L.

Distribution:—hill and submontane belt.

\# *Rhamnus saxatilis* Jacq. subsp. *saxatilis*

A **Ziziphus jujuba* Mill.—CAS

Distribution:—below Fontecchio! (pers. obs.).

ROSACEAE

\# *Agrimonia eupatoria* L. subsp. *eupatoria*

Distribution:—hill, submontane and montane belt.

\# *Alchemilla alpigena* Buser ex Hegi— D

Distribution:—Velino (Petriccione 1993).

\# **Alchemilla alpina* L.

Distribution:—Valle Majori!, near Valle Lupara!, V.ne di Sevice below the fountain! (APP 15557, 15586, 68059).

**Alchemilla alpinula* S.E.Fröhner

Distribution:—Costa delle Tavole!, Serra di Celano! (APP 67632, 67657). Confirmed for the flora of Abruzzo.

Alchemilla cinerea Buser

Distribution:—Mt. Sirente, from the beech forest to Val Lupara! (Conti et al. 2011b; APP 15517).

Alchemilla colorata Buser

Distribution:—montane belt.

**Alchemilla exigua Buser*

Distribution:—between Piani di Pezza and Cimata di Pezza (APP 67907).

Alchemilla filicaulis Buser
Distribution:—Mt. Ocre, V.ne Canavine, from the upper edge of the beech forest to the basin of Settacque!, Mt. Sirente, from the beech forest to Val Lupara! (APP 15295, 15518, 15737, 15739).

\# ***Alchemilla glaucescens*** Wallr.
Distribution:—montane belt.

E ***Alchemilla marsica*** Buser—DD
Distribution:—Campo di Rovere!, Conche di Ovindoli! (APP 66512, 68013).

Alchemilla nitida Buser
Distribution:—from montane to subalpine belt.

Alchemilla obtusa Buser
Distribution:—Altopiano delle Rocche! (APP 67838, 67839). Species new for the flora of Abruzzo.

Alchemilla strigosula Buser
Distribution:—Serra di Celano! (APP 67668).

Alchemilla subcrenata Buser
Distribution:—Prati del Sirente (Guarrera & Tammaro 1996).

Alchemilla vulgaris L.
Distribution:—Conche di Ovindoli! (APP 66520). Species new for the flora of Abruzzo.

Amelanchier ovalis Medik. subsp. ***cretica*** (Willd.) Maire & Petitm.
Distribution:—hill, submontane and montane belt.

Aphanes arvensis L.
Distribution:—hill, submontane and montane belt.

\# ***Aremonia agrimonoides*** (L.) DC. subsp. ***agrimonoides***
Distribution:—common in the montane belt.

\# ***Aria chamaemespilus*** (L.) Host (*Sorbus chamaemespilus* (L.) Crantz)
Distribution:—montane belt.

\# *Aria graeca* (Spach) M.Roem. (*Sorbus graeca* (Spach) hort. Lodd. ex S.Schauer)—D
Distribution:—V.ne di Sevice (Lucchese & Lattanzi 1993), Aielli and surroundings (Guarrera & Tammaro 1996). Its presence in the central Apennines is yet to be confirmed.

\# ***Aria edulis*** (Willd.) M.Roem. (*Sorbus aria* (L.) Crantz)
Distribution:—common from hill and submontane to subalpine belt.

Aria torminalis (L.) Beck (*Sorbus torminalis* (L.) Crantz)
Distribution:—hill and submontane belt.

Cotoneaster integerrimus Medik.
Distribution:—hill, submontane and montane belt.

\# ***Cotoneaster tomentosus*** (Aiton) Lindl.
Distribution:—montane belt.

A ***Crataegus azarolus*** L.—CAS
Distribution:—li Cerri (Rocca di Cambio)! (APP 68237).

Crataegus laevigata (Poir.) DC.
Distribution:—common from hill and submontane to montane belt.

Crataegus monogyna Jacq.
Distribution:—common from hill and submontane to montane belt.

A ***Cydonia oblonga*** Mill.—NAT
Distribution:—hill, submontane and montane belt.

Dryas octopetala L. subsp. ***octopetala***
Distribution:—montane and alpine belt.

Eriolobus florentinus (Zuccagni) Stapf
Distribution:—S. Benedetto in Perillis!, Montagna di Mandra Murata (Pirone 2015; APP 46534, 46535), *Quercus cerris* wood of Goriano Sicoli! (APP 73477).

Filipendula ulmaria (L.) Maxim.
Distribution:—hill, submontane and montane belt.

Filipendula vulgaris Moench
Distribution:—montane belt.

Fragaria vesca L. subsp. ***vesca***
Distribution:—common from hill and submontane to montane belt.

****Fragaria viridis*** Weston subsp. ***viridis***
Distribution:—Prati del Sirente!, *Quercus cerris* wood of Goriano Sicoli! (APP 9365, 67166, 72998), Terranera! (pers. obs.).

Geum heterocarpum Boiss.—CR
Distribution:—Mt. Sirente, above the region Canale (Groves 1880 from a specimens collected by Profeta as *G. pyrenaicum*; Grande 1913 from a specimens collected by Profeta; Del Prete et al. 1981 from specimens in FI), above Gagliano in Castelvecchio, Mt. Briccialone! (Guarrera & Tammaro 1996 from specimens collected by Levier; Bartolucci & Conti 2013; APP 49103, 50266). Tammaro's (1998) indication for Piani di Pezza has been rejected as erroneous and should be referred to *G. urbanum* (Bartolucci & Conti 2013). The location we have confirmed is the only one known in Italy.

Geum molle Vis. & Pančić
Distribution:—montane belt.

Geum urbanum L.
Distribution:—common from hill and submontane to montane belt.

****Hedlundia mougeotii*** (Soy.-Will. & Godr.) Sennikov & Kurtto (*Sorbus mougeotii* Soy.-Will. & Godr.)
Distribution:—Mt. Ocre, V.ne Canavine, from the upper edge of the beech forest to the basin of Settacque!, Iaccio (Rocca di Cambio)! (APP 15357, 15359, 15360, 15362, 65102, 65103, 65104, 68198, 68199, 68200, 68201, 68202).

A, # ***Malus domestica*** (Suckow) Borkh.—NAT
Distribution:—hill belt.

Malus sylvestris (L.) Mill.
Distribution:—hill belt.

Potentilla apennina Ten. subsp. ***apennina***
Distribution:—montane and alpine belt.

Potentilla caulescens L. subsp. ***caulescens***
Distribution:—from montane to subalpine belt.

Potentilla crantzii (Crantz) Beck ex Fritsch subsp. ***crantzii***

Distribution:—montane and alpine belt.

Potentilla detommasii Ten.

Distribution:—montane belt.

Potentilla erecta (L.) Raeusch.

Distribution:—montane belt.

\# *Potentilla inclinata* Vill.—D

Distribution:—Velino (Petriccione 1993).

N ***Potentilla indica*** (Andrews) Th.Wolf—CAS

Distribution:—railway station of Raiano (Stinca et al. 2021b).

\# ***Potentilla micrantha*** Ramond ex DC.

Distribution:—hill, submontane and montane belt.

Potentilla pedata Willd. ex Hornem.

Distribution:—common from hill and submontane to montane belt.

\# ***Potentilla recta*** L. subsp. ***recta***

Distribution:—hill, submontane and montane belt.

Potentilla reptans L.

Distribution:—hill, submontane and montane belt.

E, # ***Potentilla rigoana*** Th.Wolf—LC

Distribution:—common in the mountain pastures.

\# ***Poterium sanguisorba*** L. subsp. ***balearicum*** (Bourg. ex Nyman) Stace

Distribution:—common from hill and submontane to montane belt.

Poterium sanguisorba L. subsp. ***sanguisorba***

Distribution:—hill, submontane and montane belt.

A, # ***Prunus amygdalus*** Batsch—NAT

Distribution:—hill, submontane and montane belt.

Prunus avium (L.) L.

Distribution:—hill, submontane and montane belt.

A ***Prunus cerasus*** L.—CAS

Distribution:—Valle del Ceraso (Viegi et al. 1990).

A ***Prunus domestica*** L.—NAT

Distribution:—hill, submontane and montane belt.

\# ***Prunus mahaleb*** L. subsp. ***mahaleb***

Distribution:—hill, submontane and montane belt.

\# ***Prunus spinosa*** L. subsp. ***spinosa***

Distribution:—common from hill and submontane to montane belt.

Pyracantha coccinea M.Roem.

Distribution:—Gole di S. Venanzio (Ciaschetti et al. 2004; Corbetta et al. 2004), Valle di Sevice (Lucchese & Lattanzi 1993).

A, # ***Pyrus communis*** L. subsp. ***communis***—CAS

Distribution:—hill, submontane and montane belt.

Pyrus communis L. subsp. ***pyraster*** (L.) Ehrh.

Distribution:—montane belt.

****Pyrus spinosa*** Forssk.

Distribution:—Campo Valentino (Molina Aterno)! (APP 27189).

Rosa agrestis Savi

Distribution:—montane belt.

Rosa arvensis Huds.

Distribution:—hill, submontane and montane belt.

****Rosa balsamica*** Besser

Distribution:—Velino (from specimens collected by M. Iocchi in URT).

\# ***Rosa canina*** L.

Distribution:—hill, submontane and montane belt.

Rosa corymbifera Borkh.

Distribution:—Rocca di Mezzo (Tenore 1830 sub *R. dumetorum*), Valle Majelama (Lucchese & Lattanzi 1993).

****Rosa dumalis*** Bechst.

Distribution:—Mt. Sirente in Valle Pretosa and adjacent valley! (APP 27635).

Rosa gallica L.—NC

Distribution:—Velino (Tenore 1830; Montelucci 1958).

Rosa glauca Pourr.

Distribution:—Velino (Tenore 1830; Montelucci 1958; Blasi et al. 1992).

Rosa micrantha Borrer ex Sm.

Distribution:—V.ne di Sevice (Lucchese & Lattanzi 1993).

Rosa montana Chaix

Distribution:—montane belt.

\# ***Rosa pendulina*** L.

Distribution:—montane belt.

Rosa pouzinii Tratt.

Distribution:—hill and submontane belt.

Rosa sempervirens L.

Distribution:—Gole di S. Venanzio (Ciaschetti et al. 2004; Corbetta et al. 2004).

\# ***Rosa spinosissima*** L.

Distribution:—Campo Felice (De Santis & Soldati 2011), Velino (Petriccione 1993).

****Rosa squarrosa*** (A.Rau) Boreau

Distribution:— Mt. Sirente in Valle Pretosa and adjacent valley! (APP 27668).

Rosa stylosa Desv.—NC, CR

Distribution:—between Rocca di Mezzo and L'Aquila (Tenore 1831).

****Rosa subcanina*** (Christ) Vuk.

Distribution:—Mt. Sirente in Valle Pretosa and adjacent valley!, loc. Macchione (Goriano Sicoli)! (APP 27657, 29958).

****Rosa subcollina*** (Christ) Vuk.

Distribution:—S. Felice d'Ocre in loc. Cardore!, near castello di Bominaco! (APP 27087, 64569).

\# ***Rosa tomentosa*** Sm.

Distribution:—Valle Majelama, Coste del Cafornia (Lucchese & Lattanzi 1993).

\# ***Rosa villosa*** L.

Distribution:—Costa Cafornia (Cutini et al. 2002).

Rubus caesius L.

Distribution:—hill, submontane and montane belt.

Rubus canescens DC.
Distribution:—hill, submontane and montane belt.
Rubus glandulosus Bellardi (*R. hirtus* Waldst. & Kit.)
Distribution:—hill, submontane and montane belt.
Rubus idaeus L. subsp. ***idaeus***
Distribution:—hill, submontane and montane belt.
Rubus saxatilis L.
Distribution:—Vallone di Sevice, Setteselve (Avena & Blasi 1975), Valle Majori!, Neviera! (APP 15569, 36612).
Rubus ulmifolius Schott
Distribution:—common species from hill and submontane to montane belt.
Sanguisorba officinalis L.
Distribution:—Gole di Celano (Frizzi et al. 1996), Piana di Ovindoli! (Conti 1998), Campo Felice (De Santis & Soldati 2019), Val d'Arano!, Conche di Ovindoli!, Altopiano delle Rocche! (APP 11266, 11267, 28593, 66781, 67843).
Sorbus aucuparia L. subsp. ***aucuparia***
Distribution:—montane belt.
Sorbus domestica L.
Distribution:—hill and submontane belt.
****Spiraea hypericifolia*** L. subsp. ***hypericifolia***—EN
Distribution:—near Forme! (APP 68390). A steppe species of great phytogeographical interest, known in Italy only in certain areas around the Fucino basin and the surroundings of Barisciano and San Demetrio ne' Vestini. It has also been reported in Umbria but has not been confirmed since the nineteenth century (Conti & Bartolucci 2023).

RUBIACEAE
Asperula arvensis L.
Distribution:—hill, submontane and montane belt.
Asperula laevigata L.
Distribution:—montane belt.
Asperula taurina L. subsp. ***taurina***
Distribution:—montane belt.
****Crucianella angustifolia*** L.
Distribution:—Forme!, Massa d'Albe! (pers. obs.).
Cruciata glabra (L.) C.Bauhin ex Opiz
Distribution:—hill, submontane and montane belt.
Cruciata laevipes Opiz
Distribution:—hill, submontane and montane belt.
****Cruciata pedemontana*** (Bellardi) Ehrend.
Distribution:—Campo Felice!, from Rovere to Rif. La Vecchia! (APP 30123, 56209, 72784), Terranera! (pers. obs.).
Cynanchica aristata (L.f.) P.Caputo & Del Guacchio subsp. ***aristata***
Distribution:—montane belt.

Cynanchica aristata (L.f.) P.Caputo & Del Guacchio subsp. **_scabra_** (Nyman) P.Caputo & Del Guacchio
Distribution:—montane belt.
Cynanchica pyrenaica (L.) P.Caputo & Del Guacchio subsp. **_cynanchica_** (L.) P.Caputo & Del Guacchio
Distribution:—montane belt.
E, # **_Cynanchica pyrenaica_** (L.) P.Caputo & Del Guacchio subsp. **_neglecta_** (Guss.) P.Caputo & Del Guacchio—LC
Distribution:—high altitudes of Velino and Mt. Sirente.
Galium album Mill. subsp. **_album_**
Distribution:—hill, submontane and montane belt.
Galium anisophyllon Vill.
Distribution:—montane and alpine belt.
Galium aparine L.
Distribution:—hill, submontane and montane belt.
Galium corrudifolium Vill.
Distribution:—hill, submontane and montane belt.
Galium debile Desv.
Distribution:—Gole di Celano (Frizzi et al. 1996), Prati del Lago!, below Forca Caruso towards Fucino! (APP 62186, 67708).
Galium divaricatum Pourr. ex Lam.
Distribution:—Pié di Sevice (Avena & Blasi 1975).
Galium lucidum All. subsp. **_lucidum_**
Distribution:—montane belt.
E, # **_Galium magellense_** Ten.—LC
Distribution:—high altitude screes of Velino and Mt. Sirente.
Galium megalospermum All.—D
Distribution:—Velino (Tenore 1831).
Galium mollugo L.
Distribution:—montane belt.
Galium odoratum (L.) Scop.
Distribution:—montane belt.
*Galium murale (L.) All.
Distribution:—Molina Aterno! (APP 73575).
*Galium palustre L. subsp. **_palustre_**
Distribution:—Alopiano delle Rocche, Piana di Ovindoli! (Ciaschetti et al. 2005; APP 66522), laghetto d’Arano!, Campo di Rovere! (APP 28622, 34715, 67224).
Galium parisiense L.
Distribution:—hill and submontane belt.
Galium spurium L.
Distribution:—montane belt.
Galium tricornutum Dandy
Distribution:—montane belt.
*Galium verticillatum Danthoine
Distribution:—Colle della Forchetta! (pers. obs.).

Galium verum L. subsp. **_verum_**
Distribution:—hill, submontane and montane belt.
Rubia peregrina L.
Distribution:—Gole di S. Venanzio! (D'Errico 1936; Pirone & Tammaro 1997; Ciaschetti et al. 2004; Corbetta et al. 2004; APP 35700). The report from Rocca di Cambio (Cutini & Blasi 2002) is unlikely and probably incorrect.

A **_*Rubia tinctorum_** L.—CAS
Distribution:—Succiano!, Fontecchio! (pers. obs.).
Sherardia arvensis L.
Distribution:—hill, submontane and montane belt.

Thliphthisa purpurea (L.) P.Caputo & Del Guacchio subsp. **_purpurea_**
Distribution:—hill, submontane and montane belt.

RUTACEAE
Ruta graveolens L.
Distribution:—hill belt.

SALICACEAE
N **_*Populus ×canadensis_** Moench nothosubsp. **_canadensis_**—CAS
Distribution:—Conche di Ovindoli near the rif. degli alpini!, Lago di Molina Aterno! (APP 66480, 66484, 73035).
Populus alba L.
*Populus nigra L. subsp. **_neapolitana_** (Ten.) Maire
Distribution:—F. Aterno near the bridge of Villa S. Angelo! and near Tione degli Abruzzi!, Lago di Civita! (APP 26806, 28244, 34862).
Populus nigra L. subsp. **_nigra_**
Distribution:—hill, submontane and montane belt.

Populus tremula L.
Distribution:—hill, submontane and montane belt.
Salix alba L.
Distribution:—hill, submontane and montane belt.
Salix amplexicaulis Bory
Distribution:—Campo Felice (De Santis & Soldati 2019).
Salix apennina A.K.Skvortsov
Distribution:—hill, submontane and montane belt.
Salix breviserrata Flod.—D
Distribution:—Piani di Pezza, Neviera (Rovelli & Conti 1995). The reports are not supported by herbarium specimens, and the species has not been found again in the locations indicated. The plant is probably not present in the Park.

Salix caprea L.
Distribution:—hill, submontane and montane belt.

Salix cinerea L.
Distribution:—Velino (Petriccione 1993; Pirone 1995), Gole di Celano (Frizzi et al. 1996; Guarrera & Tammaro 1996), Campo di Rovere (Ciaschetti et al. 2005), Conche di Ovindoli! (APP 73536), F. Aterno near Campana! (pers. obs.).

Salix eleagnos Scop.
Distribution:—hill, submontane and montane belt.
Salix purpurea L. subsp. ***purpurea***
Distribution:—hill, submontane and montane belt.
Salix retusa L.
Distribution:—Mt. Sirente (Guarrera & Tammaro 1996), Velino (Tenore 1831).
Salix triandra L. subsp. ***triandra***
Distribution:—hill and submontane belt.

SANTALACEAE
Osyris alba L.
Distribution:—hill and submontane belt.
\# ***Thesium alpinum*** L.
Distribution:—Iaccetto di Capo di Pezza (Avena & Blasi 1975).
Thesium bavarum Schrank
Distribution:—hill, submontane and montane belt.
\# ***Thesium humifusum*** DC.
Distribution:—hill, submontane and montane belt.
Thesium linophyllon L.
Distribution:—hill, submontane and montane belt.
\# ***Thesium parnassi*** A.DC.
Distribution:—from montane to subalpine belt.
Viscum album L. subsp. ***album***
Distribution:—hill, submontane and montane belt.

SAPINDACEAE
\# ***Acer campestre*** L.
Distribution:—hill, submontane and montane belt.
\# ***Acer monspessulanum*** L. subsp. ***monspessulanum***
Distribution:—hill, submontane and montane belt.
\# ***Acer opalus*** Mill. subsp. ***obtusatum*** (Waldst. & Kit. ex Willd.) Gams
Distribution:—hill, submontane and montane belt.
Acer platanoides L.
Distribution:—montane belt.
\# ***Acer pseudoplatanus*** L.
Distribution:—montane belt.
N ***Aesculus hippocastanum*** L.—NAT
Distribution:—base of Mt. Ventola, along a ditch on the edge of the SP 9 road near the cemetery of Goriano Sicoli! (Conti et al. 2025a; APP 72943).

SAXIFRAGACEAE
Saxifraga adscendens L. subsp. ***adscendens***
Distribution:—montane and alpine belt.
\# ***Saxifraga adscendens*** L. subsp. ***parnassica*** (Boiss. & Heldr.) Hayek
Distribution:—montane belt.

Saxifraga bulbifera L.
Distribution:—hill, submontane and montane belt.
Saxifraga caesia L.
Distribution:—montane and alpine belt.
\# ***Saxifraga callosa*** Sm.
Distribution:—from montane to subalpine belt.
E, # ***Saxifraga exarata*** Vill. subsp. ***ampullacea*** (Ten.) D.A.Webb—LC
Distribution:—cliffs of the summit of Velino and Mt. Sirente.
Saxifraga glabella Bertol.—DD
Distribution:—summit of Mt. Sirente (Groves 1880, Guarrera & Tammaro 1996).
\# ***Saxifraga granulata*** L. subsp. ***granulata***
Distribution:—hill, submontane and montane belt.
E, # *Saxifraga italica* D.A.Webb—D, NT
Distribution:—Velino (Petriccione 1993), summit of Mt. Sirente (Guarrera & Tammaro 1996). The existing reports are not supported by herbarium specimens.
Saxifraga marginata Sternb.—NC
Distribution:—Velino (Montelucci 1958).
\# ***Saxifraga paniculata*** Mill.
Distribution:—from hill and submontane to subalpine belt.
E, # ***Saxifraga porophylla*** Bertol. subsp. ***porophylla***—LC
Distribution:—Velino and Mt. Sirente cliffs from the mountain to the alpine belt.
\# ***Saxifraga rotundifolia*** L. subsp. ***rotundifolia***
Distribution:—hill, submontane and montane belt.
E, # ***Saxifraga speciosa*** Dörfl. & Hayek—LC
Distribution:—Velino and Mt. Sirente cliffs of the alpine belt.
Note:—the previous reports of *S. oppositifolia* should be referred to this taxon.
Saxifraga tridactylites L.
Distribution:—hill, submontane and montane belt.

SCROPHULARIACEAE
Scrophularia auriculata L. subsp. ***auriculata***
Distribution:—above Piani di Canale, Ovindoli (Guarrera & Tammaro 1996).
\# ***Scrophularia canina*** L.
Distribution:—hill, submontane and montane belt.
\# ***Scrophularia juratensis*** Schleich.
Distribution:—montane and alpine belt.
Scrophularia nodosa L.
Distribution:—montane belt.
Scrophularia peregrina L.
Distribution:—reported for the neighbouring territory of Lucoli (De Santis & Soldati 2019) and likely also present in the Park.
\# ***Scrophularia scopolii*** Hoppe ex Pers.
Distribution:—montane belt.
Scrophularia umbrosa Dumort. subsp. ***umbrosa***

Distribution:—Mt. Sirente (Groves 1880 as *S. aquatica*), F. Aterno near Fossa! and near Tione! (APP 26760, 28199, 28245).

Scrophularia vernalis L.

Distribution:—hill, submontane and montane belt.

Verbascum blattaria L.

Distribution:—hill and submontane belt.

Verbascum chaixii Vill. subsp. ***chaixii***

Distribution:—between Celano and Ovindoli (Guarrera & Tammaro 1996).

Verbascum densiflorum Bertol.

Distribution:—hill, submontane and montane belt.

Verbascum longifolium Ten.

Distribution:—montane belt.

Verbascum lychnitis L.

Distribution:—montane belt.

Verbascum macrurum Ten.

Distribution:—Coste del Cafornia (Lucchese & Lattanzi 1993).

Verbascum mallophorum Boiss. & Heldr.

Distribution:—hill, submontane and montane belt.

Verbascum nigrum L.

Distribution:—Velino (Bertoloni 1833-54), Gole di Celano (Frizzi et al. 1996, Guarrera & Tammaro 1996).

E ***Verbascum niveum*** Ten. subsp. ***garganicum*** (Ten.) Murb.—DD

Distribution:—Mt. Sirente (Murbeck 1933), garrigue along Subequana valley! (pers. obs.).

Verbascum niveum Ten. subsp. ***niveum***

Distribution:—Magliano dei Marsi (Avena & Blasi 1975)

Verbascum phoeniceum L.

Distribution:—Mt. Sirente, in front of "Il Lago" in loc. Corvaro (Conti 1993; APP 28496).

Verbascum pulverulentum Vill.

Distribution:—hill and submontane belt.

Verbascum sinuatum L.

Distribution:—hill and submontane belt.

Verbascum thapsus L. subsp. ***thapsus***

Distribution:—hill, submontane and montane belt.

SIMAROUBACEAE

N ***Ailanthus altissima*** (Mill.) Swingle—INV

Distribution:—common species from hill and submontane to montane belt.

SMILACACEAE

Smilax aspera L.

Distribution:—Gole di S. Venanzio (Corbetta et al. 2004).

SOLANACEAE

Atropa bella-donna L.
Distribution:—montane belt.
N ***Datura stramonium*** L.—NAT
Distribution:—hill and submontane belt.
Hyoscyamus niger L.
Solanum dulcamara L.
Distribution:—hill and submontane belt.
N ****Solanum lycopersicum*** L.—CAS
Distribution:—near Prati del Sirente! (pers. obs.).
Solanum nigrum L.
Distribution:—hill and submontane belt.
****Solanum villosum*** Mill.
Distribution:—Fontecchio! (APP 74218).

STAPHYLEACEAE
Staphylea pinnata L.
Distribution:—Bosco Defensa of Molina (Pirone 2015 from Cianfaglione).

THYMELAEACEAE
Daphne alpina L. subsp. ***alpina***
Distribution:— montane belt.
Daphne laureola L.
Distribution:—common species from hill and submontane to montane belt.
Daphne mezereum L.
Distribution:—common species from hill and submontane to montane belt.
Daphne oleoides Schreb. subsp. ***oleoides***
Distribution:—common species from hill and submontane to subalpine belt.
Daphne sericea Vahl
Distribution:—Raiano (Anguillara 1561), Gole di S. Venanzio!, La Liscia (Castelvecchio Subequo) (Tenore 1831; D'Errico 1936; Montelucci 1971; Pirone 1995; Pirone et al. 1997; Conti 1998; Pirone & Cutini 2002; Ciaschetti et al. 2004; Corbetta et al. 2004; APP 35701, 66902).
Thymelaea passerina (L.) Coss. & Germ.—NC
Distribution:—cliffs of Rio below Castelvecchio Subequo (Groves 1880).

TYPHACEAE
Sparganium erectum L.
Distribution:—along F. Aterno (Groves 1880), little lakes near Rocca di Cambio (Buchwald 1995).
Sparganium neglectum Beeby
Distribution:—hill, submontane and montane belt.
Sparganium oocarpum (Čelak.) Fritsch—NC
Distribution:—Lago di Molina (Lastrucci et al. 2024 from a specimen collected by Groves).
Typha angustifolia L.

Distribution:—hill and submontane belt.
Typha latifolia L.
Distribution:—hill, submontane and montane belt.

ULMACEAE
Ulmus glabra Huds.
Distribution:—hill, submontane and montane belt.
Ulmus minor Mill. subsp. ***minor***
Distribution:—hill, submontane and montane belt.

URTICACEAE
Parietaria judaica L.
Distribution:—hill, submontane and montane belt.
Parietaria officinalis L.
Distribution:—hill, submontane and montane belt.
Urtica dioica L.
Distribution:—common species from hill and submontane to subalpine belt.
Urtica membranacea Poir.
Distribution:—Mt. Sirente (Groves 1880), near laghetto Fonte dell'Acqua (Guarrera & Tammaro 1996).
Urtica pilulifera L.
Distribution:—Mt. Sirente (Groves 1880), beech forest clearings 3 km from Rocca di Mezzo (Guarrera & Tammaro 1996).
****Urtica urens*** L.
Distribution:—Acciano! (APP 74175).

VALERIANACEAE
Valeriana angustifolia Mill. (*Centranthus angustifolius* (Mill.) DC. subsp. *angustifolius*)
Distribution:—Mt. Sirente (Groves 1880; Grande 1913 from a specimen collected by Profeta), Valle Pretosa!, Valle Inserrata! (APP 27648, 27649, 27650, 27651, 28546).
****Valeriana rubra*** L. (*Centranthus ruber* (L.) DC. subsp. *ruber*)
Distribution:—hill and submontane belt.
Valeriana montana L.
Distribution:—montane and alpine belt.
Valeriana officinalis L. subsp. ***officinalis***
Distribution:—hill, submontane and montane belt.
Valeriana saliunca All.
Distribution:—summit of Mt. Velino! (Tenore 1830, 1831; Montelucci 1958; Petriccione 1993, 1994; APP 34606) and Mt. Sirente (Groves 1880; Guarrera & Tammaro 1996).
Valeriana stolonifera Czern. subsp. ***angustifolia*** Soó
Distribution:—hill, submontane and montane belt.
Valeriana tripteris L. subsp. ***tripteris***

Distribution:—hill, submontane and montane belt.

Valeriana tuberosa L.

Distribution:—*Valeriana dioica* has been reported for Mt. Sirente, Gole di Celano, Mt. Ventrino (Collarmele) (Groves 1880, Frizzi et al. 1996; Guarrera & Tammaro 1996). The revision of the specimens kept in the Guarrera herbarium allowed us to refer them to *V. tuberosa* . *Valeriana dioica* is to be excluded from Mt. Sirente. In the only other known location in Abruzzo (i.e. Campotosto), the species is to be considered extinct (Conti & Tinti 2008).

Valerianella carinata Loisel.
Distribution:—hill and submontane belt.

****Valerianella coronata*** (L.) DC.
Distribution:—near Fossa!, near Castevecchio Subequo!, near Terranera! (APP 34229, 67066, 73017), Forme! (pers. obs.).

Valerianella dentata (L.) Pollich
Distribution:—hill, submontane and montane belt.

Valerianella discoidea (L.) Loisel.
Distribution:—Mt. Ventrino (Guarrera & Tammaro 1996).

****Valerianella eriocarpa*** Desv.
Distribution:—Massa d’Albe!, Collarmele! (pers. obs.).

Valerianella locusta (L.) Laterr.
Distribution:—hill, submontane and montane belt.

****Valerianella puberula*** (Bertol. ex Guss.) DC.
Distribution:—Pié di Sevice! (APP 73427).

\# ***Valerianella pumila*** (L.) DC.
Distribution:—hill, submontane and montane belt.

Valerianella rimosa Bastard
Distribution:—hill, submontane and montane belt.

VERBENACEAE

Verbena officinalis L.
Distribution:—hill and submontane belt.

VIBURNACEAE

\# ***Adoxa moschatellina*** L. subsp. ***moschatellina***
Distribution:—hill, submontane and montane belt.

\# ***Sambucus ebulus*** L.
Distribution:—common species from hill and submontane to montane belt.

\# ***Sambucus nigra*** L.
Distribution:—common species from hill and submontane to montane belt.

\# ***Viburnum lantana*** L.
Distribution:—montane belt.

Viburnum tinus L. subsp. ***tinus***
Distribution:—Gole di S.Venanzio! (D’Errico 1936; Ciaschetti et al. 2024; Corbetta et al. 2024; APP 28504, 35704).

VIOLACEAE

Viola alba Besser subsp. ***dehnhardtii*** (Ten.) W.Becker

Distribution:—common species from hill and submontane to montane belt.

Viola arvensis Murray

Distribution:—hill and submontane belt.

Viola canina L.

Distribution:—Prati del Sirente (Tammaro et al. 1974), Campo di Rovere! (APP 34796, 46699, 68394).

E, # ***Viola eugeniae*** Parl. subsp. ***eugeniae***—LC

Distribution:—very common in the secondary and primary grasslands of Velino and Mt. Sirente.

E, # ***Viola eugeniae*** Parl. subsp. ***levieri*** (Parl.) Arcang.—LC

Distribution:—Rocca di Cambio (Fiori & Béguinot 1917), Velino (Montelucci 1958), near Massa d'Albe!, near Raiano, near Molina (Tammaro & Frizzi 1990; APP 73895), Gole di Celano (Frizzi et al. 1996; Guarrera & Tammaro 1996), Aielli and surroundings, Prati del Sirente, surroundings of Celano, Mt. Ventrino, Mt. Revecena, from S. Panfilo to Terranera (Guarrera & Tammaro 1996), Piani di Pezza (Ciaschetti et al. 2006), Mt. Offermo!, near Fossa!, between Roccapreturo and the tower! (APP 59814, 68032, 73031).

Viola kitaibeliana Schult.—EN

Distribution:—hill and submontane belt.

Viola odorata L.

Distribution:—hill, submontane and montane belt.

Viola reichenbachiana Jord. ex Boreau

Distribution:—common species from hill and submontane to montane belt.

Viola riviniana Rchb.

Distribution:—hill, submontane and montane belt.

Viola suavis M.Bieb.

Distribution:—common species from hill and submontane to montane belt.

Viola tricolor L.

Distribution:—Mt. Sirente (Groves 1880), Gole di S. Venanzio (D'Errico 1936), S. Panfilo d'Ocre!, Castelvecchio Subequo! (pers. obs.).

VITACEAE

N ***Vitis ×instabilis*** Ardenghi, Galasso, Banfi & Lastrucci—NAT

Distribution:— Gole di S. Venanzio! (Conti et al. 2008a as *V. rupestris;* APP 25290).

N ***Vitis riparia*** Michx.—NAT

Distribution:—hill and submontane belt.

Vitis vinifera L.

Distribution:—hill and submontane belt.

ZYGOPHYLLACEAE

Tribulus terrestris L.—NC

Distribution:—Mt. Sirente (Groves 1880).

References

Abbate E., 1903 - La Flora. Guida dell'Abruzzo. C.A.I. Roma. Pp 62-115

Adamovic L., 1933 - Die Pflanzengeographische Stellung und Gliederung Italiens. Fischer ed., Jena. 259 pp.: 128-137.

Aedo C., 1996 - Revision of *Geranium* subgenus *Erodioidea* (Geraniaceae). Systematic Botany Monographs, 49: 1-99.

Anguillara L., 1561 – Semplici dell’eccellente M. Luigi Anguillara, Vincenzo Valgrifi, Venezia.

Anzalone B., 1961 - Su alcune piante interessanti di Scanno e di altre località d'Abruzzo. Nuovo Giornale Botanico Italiano, n.s., 67 (3-4) (1960): 550-556.

Anzalone B., Lattanzi E., Leporatti M.L., 1992 - Il gruppo di *Ferula communis* L. (Umbelliferae) in Italia: ricerche sistematiche e corologiche. Archivio Botanico e Biogeografico Italiano, 67 (3-4) (1991): 221-236.

Avena G.C., Blasi C., 1975 - Un contingente di specie non segnalate nel Massiccio del M. Velino (Appennino Abruzzese): loro ambientazione geomorfologica e vegetazionale. Annali di Botanica (Roma), 33 (1974): 41-82.

Avena G. C., Blasi C., 1980 - Carta della vegetazione del Massiccio del Monte Velino. Appennino Abruzzese. C.N.R., Coll. Progr. Final. "Promozione della Qualità dell'ambiente", AQ/1/35. Roma. 18 pp. + map.

Ballelli S., Pedrotti F. 1979 - In Gruppo di Lavoro per la Conservazione della Natura della Società Botanica Italiana- Censimento dei biotopi di rilevante interesse vegetazionale meritevoli di conservazione in Italia. Vol. II. Tip. Succ. Savini-Mercuri, Camerino. 585 pp.

Bartolucci F., 2010 - Notule Pteridologiche Italiche. VIII: 182. *Dryopteris pallida* (Bory) C. Chr. ex Maire & Petitm. subsp. *pallida* . Annali del Museo civico di Rovereto. Sez.: Arch., St., Sc. nat., 25 (2009): 105-106.

Bartolucci F., Conti F., 2013 - Taxonomical notes on *Geum micropetalum* Gasp. and *Geum heterocarpum* Boiss. (Rosaceae) from Italy. Plant Biosystems, 147 (3): 806-811.

Bartolucci F., Conti F., Santucci B., Ciamaglione K., 2013 - Notulae alla checklist della flora vascolare italiana 15. 1990. Informatore Botanico Italiano, 45(1): 103

Bartolucci F., Stinca A., Tinti D., Conti F., 2014 - Beni Ambientali Individui ai sensi del Piano del Parco Nazionale del Gran Sasso e Monti della Laga. Relazione finale dello studio sulle emergenze floristiche del Parco.

Bartolucci F., Domina G., Adorni M., Alessandrini A., Ardenghi N.M.G., Banfi E., Baragliu G.A., Bernardo L., Bertolli A., Biondi E., Carotenuto L., Casavecchia S., Cauzzi P., Conti F., Crisanti M.A., D’Amico F.S., Di Cecco V., Di Martino L., Faggi G., Falcinelli F., Forte L., Galasso G., Gasparri R., Ghillani L., Gottschlich G., Guzzon F., Harpke D., Lastrucci L., Lattanzi E., Maiorca G., Marchetti D., Medagli P., Olivieri N., Pascale M., Passalacqua N.G., Peruzzi L., Picollo S., Prosser F., Ricciardi M., Salerno G., Stinca A., Terzi M., Viciani D., Wagensommer R.P., Nepi C., 2017 - Notulae to the Italian native vascular flora: 3. Italian Botanist, 3: 29–48. doi:10.3897/italianbotanist.3.13200

Bartolucci F, Iocchi M, De Castro O, Conti F., 2022 - *Allium ducissae* (*A.* subgen. *Polyprason*, Amaryllidaceae) a New Species from the Central Apennines (Italy). Plants, 11, 426. https://doi.org/ 10.3390/plants11030426

Bartolucci F., Peruzzi L., Galasso G., Alessandrini A., Ardenghi N.M.G., Bacchetta G., Banfi E., Barberis G., Bernardo L., Bouvet D., Bovio M., Calvia G., Castello M., Cecchi L., Del Guacchio E., Domina G., Fascetti S., Gallo L., Gottschlich G., Guarino R., Gubellini L., Hofmann N., Iberite M., Jimenez-Mejias P., Longo D., Marchetti D., Martini F., Masin R.R., Medagli P., Peccenini S., Prosser F., Roma-Marzio F., Rosati L., Santangelo A., Scoppola A., Selvaggi A., Selvi F., Soldano A., Stinca A., Wagensommer R.P., Wilhalm T., Conti F., 2024a - A second update to the checklist of the vascular flora native to Italy. Plant Biosystems, 158(2):219-296.

Bartolucci F., Domina G., Buccomino G., Ciaschetti G., Conti F., Costanza N., De Luca A., Del Guacchio E., Falcinelli F., Forte L., Galasso G., Ganz C., Iamonico D., Lonati M,. Marengo G., Mei G., Nota G., Orsenigo S., Pazienza G., Pellegrino G., Pinzani L., Stinca A., Tavilla G., Tilia A., Tomaselli V., Tondi G., Venanzoni R., Lastrucci L., 2024b - Notulae to the Italian native vascular flora: 18. Italian Botanist, 18: 97-108. https://doi.org/10.3897/italianbotanist.18.140958

Bartolucci F, Domina G, Angiolini C, Argenti C, Bacchetta G, Barberis D, Bertotto G, Bonari G, Calvia G, Candini F, Coltri F, Conti F, Del Guacchio E, Di Pietro R, Festi F, Fois M, Forte L, Galasso G, Gallo Splendore M, Koopman J, Lonati M, Mascia F, Minutillo F, Nascimbene J, Nota G, Pappagallo G, Pellegrino G, Podda L, Roffarè G, Ruggero A, Selvi F, Silletti G, Soldano A, Terranova C, Tondi G, Vallariello R, Zanatta K, Lastrucci L (2025) Notulae to the Italian native vascular flora: 20. Italian Botanist 20: 89-107. https://doi.org/10.3897/italianbotanist.20.179511

Bazzichelli G., 1972 - *Achillea barrelieri* (Ten.) Sch.-Bip. (emend.Heimerl) ssp. *barrelieri* forma *schouwii* (DC.) Bazzichelli n. comb. (Compositae): Sviluppo del gametofito femminile. Revisione sistematica e nomenclatura. Distribuzione geografica. Annali di Botanica (Roma), 29 (1967-1969): 31-85.

Bernardo L., Gargano D., Peruzzi L., 2005 - Problemi nella delimitazione delle specie in *Campanula* L. subsect. *Heterophylla* (Wit.) Fed. Inform. Bot. Ital., 36 (2) (2004): 516-520.

Bertoloni A., 1833-54 - Flora Italica 1-10. Tip. R. Masi, Bologna.

Blasi C., Gigli M.P., Stanisci A., 1992 - I cespuglieti altomontani del gruppo del M. Velino (Italia centrale). Annali di Botanica (Roma), 48 (1990), Suppl. 7: 243-261.

Brocchi G.B. 1823 – Osservazioni naturali fatte in alcune parti degli Appennini nell'Abruzzo Ulteriore. Parte III. Giornale di Letteratura, Scienze ed Arti, 29: 79-93.

Buchwald R., 1995 - Vegetazione e odonatofauna negli ambienti acquatici dell'Italia centrale. Braun-Blanquetia, 11 (1994): 1-77.

Catonica C., 2001 - A new species and a new record of *Festuca* (Poaceae) from the Gran Sasso of Italy (Central Apennines). Plant Biosystems, 135 (3): 271-284.

Chaudhri M.N., 1968 - A revision of the Paronychineae. Mededeelingen van het Botanisch Museum en Herbarium van de Rijks Universiteit te Utrecht, 285: 1-440

Ciaschetti G., 2003 - Segnalazioni Floristiche Italiane: 1062-1064. 1062. *Gentiana pneumonanthe* L. (Gentianaceae); 1063. *Serratula lycopifolia* (Vill.) A. Kerner (Asteraceae); 1064. *Sesleria caerulea* (L.) Ard. (Gramineae). Informatore Botanico Italiano, 35 (1): 101-102.

Ciaschetti G., Pirone G., 2019. *Lathyro asphodeloidis-Klaseetum lycopifoliae,* a new plant association in the alliance *Cynosurion cristati* Tüxen, 1947 in Central Apennines. Italian Botanist, 7: 35-50.

Ciaschetti G., Di Martino L., Frattaroli A.R., Pirone G., 2004 - La vegetazione a leccio (*Quercus ilex* L.) in Abruzzo (Italia centrale). Fitosociologia, 41 (1): 77-86

Ciaschetti G., Pirone G., Corbetta F., Frattaroli A. R., Di Martino L. 2005 - Il Paesaggio vegetale dell'Altopiano delle Rocche: classificazione gerarchica del territorio e serie di vegetazione. In: G. Tamburini (ed.), Altipiani. Modelli di monitoraggio e di pianificazione dei sistemi territoriali dell'Appennino centrale. Gangemi Editore, Roma: 45-84.

Ciaschetti G., Pirone G., Frattaroli A.R., Corbetta F., 2006 - La vegetazione del Piano di Pezza (Parco Naturale Regionale "Sirente-Velino" - Italia Centrale). Fitosociologia, 43 (1): 67-84.

Ciaschetti, G., Praleskouskaya, S., Venanzoni, R. 2024 - Relicts of Threatened Biodiversity: Similarities and Differences among the 7230 EU Habitat Plant Communities on Montane Plateaus of Central Apennines, Italy. Plants 2024, 13, 1282. https://doi.org/10.3390/plants13101282

Conti F., 1993 - Note floristiche per l'Italia centro-meridionale. Archivio Botanico e Biogeografico Italiano, 68 (1-2) (1992): 26-34.

Conti F., 1997 - *Minuartia glomerata* subsp. *trichocalycina* comb. & stat. nov. (Caryophyllaceae), endemic entity of Abruzzo (Central Italy). Willdenowia, 27: 73-79.

Conti F., 1998 - An annotated checklist of the flora of the Abruzzo. Bocconea, 10: 276 pp.

Conti F., 2003 - *Minuartia graminifolia* (Caryophyllaceae), a south-east European species. Bot. J. Linn. Soc., 143: 419-432.

Conti F., Bartolucci F., 2011 - Notulae alla checklist della flora vascolare italiana 11. 1782-1793. Informatore Botanico Italiano, 43 (1): 132-135.

Conti F., Bartolucci F., 2021 - *Anthyllis apennina* (Fabaceae), a new species from central Apennine (Italy). PhytoKeys,176: 111–129. https://doi.org/10.3897/phytokeys.176.62774

Conti, F.; Bartolucci, F. 2023 - Taxonomy and distribution of *Spiraea hypericifolia* in Italy and typification of the name *S. flabellata* (Rosaceae). Plants, 12, 536. https://doi.org/10.3390/plants12030536

Conti F., Di Martino L. (eds.) 2021 - Life Floranet. La salvaguardia delle piante di interesse comunitario dell'appennino centrale. Parco Nazionale della Majella, 208 pp.

Conti F., Manzi A., 1996 - Note floristiche per Abruzzo, Molise e Puglia. Archivio Geobotanico, 2 (1): 83-90.

Conti F., Manzi A., 1997 - *Serratula lycopifolia* (Vill.) A. Kern., new for the Italian flora. Flora Mediterranea, 7: 181-183

Conti F., Manzi A., 1998 - Segnalazioni floristiche per Abruzzo, Marche e Puglia. Archivio Geobotanico, 3 (1) (1997): 107-116.

Conti F., Peruzzi L., 2006 - *Pinguicula* L. (*Lentibulariaceae*) in central Italy: taxonomic study. Annales Botanici Fennici, 43: 321-337.

Conti F., Tinti D., 2008 - Il Lago di Campotosto e la sua Flora. Litografia Brandolini. Sambuceto di S. Giovanni Teatino (CH). 160 pagg.

Conti F., Manzi A., Pirone G., 1999 - Note floristiche per l'Abruzzo. Informatore Botanico Italiano, 30 (1-3) (1998): 15-22.

Conti F., Manzi A., Tinti D., 2002 - Aggiunte alla Flora d'Abruzzo. Informatore Botanico Italiano, 34 (1): 55-61.

Conti F., Bartolucci F., Catonica C., D'Orazio G., Londrillo I., Manzi A., Tinti D., 2006 - Aggiunte alla flora d'Abruzzo. II° contributo. Informatore Botanico Italiano, 38 (1): 113-116.

Conti F., Bartolucci F., Manzi A., Miglio M., Tinti D., 2008a - Aggiunte alla Flora d'Abruzzo: III contributo. Annali del Museo civico di Rovereto., Sez.: Arch., St., Sc. Nat., 23(2007): 127-140.

Conti F., Di Santo D., Giovi E., Tinti D., 2008b - *Goniolimon italicum* F. Tammaro, Pignatti & G. Frizzi. Informatore Botanico Italiano, 40, suppl. 1: 79-81

Conti F., Miglio M., Santucci B., 2011a - Notulae alla checklist della flora vascolare italiana 11. 1797-1798. Informatore Botanico Italiano, 43 (1): 136.

Conti F., Bartolucci F., Tondi G., 2011b - Notulae alla checklist della flora vascolare italiana 11. 1803. Informatore Botanico Italiano, 43 (1): 137-138.

Conti F., Bartolucci F., Tomović G., Lakušić D., 2012 - *Jacobaea vulgaris* subsp. *gotlandica* (Compositae), new for Italy and Montenegro. Botanica Serbica, 36(2): 145-147.

Conti F., Bartolucci F., Manzi A., Paolucci M., Santucci B., Petriccione B., Ciaschetti G., Stinca A., 2016 - Integrazioni alla flora vascolare dell'Italia centrale. Atti della Società Toscana di Scienze Naturali, Mem., Serie B, 122 (2015): 33-42

Conti F., Pennesi R., Uzunov D., Bracchetti L., Bartolucci F., 2018a - A new species of *Oxytropis* (Fabaceae) from Central Apennines (Italy). Phytotaxa, 336 (1): 69-81.

Conti F., Falcinelli F., Palermo D., Paolucci M., Paris P., Domina G., Manzi A., Gallo L., Tondi G., Pennesi R., Donnini D., Bartolucci F., 2018b - Integrazioni alla flora vascolare dell'Italia centrale. Secondo contributo. Natural History Sciences, 5 (1): 59-70.

Conti F., Falcinelli F., Giacanelli V., Paolucci M., Pirone G., Proietti E., Stinca A., Bartolucci F., 2019a - New floristic data of vascular plants from central and southern Italy. Flora Mediterranea, 29: 215-222.

Conti F., Bracchetti L., Uzunov D., Bartolucci F., 2019b - A new subspecies of *Corydalis densiflora* (Papaveraceae) from the Apennines (Italy). Willdenowia, 49(1): 53–64.

Conti F., Bartolucci F., Bacchetta G., Pennesi R., Lakušić D., Niketić M., 2021 - A taxonomic revision of the *Siler montanum* group (Apiaceae) in Italy and the Balkan Peninsula. – Willdenowia, 51: 321 – 347.

Conti F., Cangelmi G., Da Valle J., De Santis E., Giacanelli V., Gubellini L., Hofmann N., Masin R.R., Miglio M., Palermo D., Santucci B., Bartolucci F., 2023a - Additions to the vascular flora of Italy. — Flora Mediterranea, 33: 177-191.

Conti F., Falcinelli F., Giacanelli V., Santucci B., Miglio M., Manzi A., Bartolucci F, 2023b - New floristic data of vascular plants from central Italy. Natural History Sciences 10(1): 51–56.

Conti F., Falcinelli F., Bracchetti L., De Santis E., Guiggi A., Manzi A., Palermo D., Munoz-Rodriguez P., Wood J.R.I., Bartolucci F., 2025a - New additions to the vascular flora of central and southern Italy. Flora Mediterranea, 35: 5-14.

Conti F., Bracchetti L., Dorfner M., Schopf R., Benda N., Oberprieler C., 2025b - Contribution to the Knowledge of *Leontodon* Sect. *Asterothrix* (Cass.) Ball in Italy and on the Balkan Peninsula. Biology 14, 1263. https://doi.org/10.3390/biology14091263

Corbetta F., Brucculieri R., Ciaschetti G., Frattaroli A.R., Pirone G., 2004 - Le serie di vegetazione nella media valle dell'Aterno (Abruzzo). Colloques Phytosociologiques, 28 (1998): 747-762.

Costalonga S., 2004 - Segnalazioni Floristiche Italiane: 1131. *Carex vulpina* L. (Cyperaceae). Informatore Botanico Italiano, 36 (1): 91.

Cutini M., Blasi C., 2002 - Contributo alla definizione sintassonomica e sindinamica dei mantelli di vegetazione della fascia collinare-submontana dell'Appennino centrale (Italia centrale). Fitosociologia, 39 (1), suppl. 2: 97-120.

Cutini M., Stanisci A., Pirone G., 2002 - L'alleanza *Berberidion vulgaris* in Appennino centrale (Italia centrale). Fitosociologia, 39 (2): 31-50.

De Angelis G., Scacchi R., 1990 - Segnalazioni Floristiche Italiane: 537-539. 537. *Epipactis persica* (Soò) Nannfeldt (Orchidaceae); 538. *Dactylorhiza x altobracensis* (Coste) Soò (Orchidaceae); 539. *Orchis spitzelii* Sauter ex Koch (Orchidaceae). Informatore Botanico Italiano, 20 (2-3) (1988): 658-660.

D'Errico P., 1936 - Elementi mediterranei nella vegetazione della conca aquilana. Alpe (Firenze) 23: 288-292.

Del Carratore F., Garbari F., 2003 - Il Gen. *Salvia* Sect. *Plethiosphace* (Lamiaceae) in Italia. Archivio Geobotanico, 7 (1) (2001): 41-62.

Del Prete C., Donini A.M., Garbari F., 1981 - Quisquiliae Floristicae Apenninae: 1-5. Atti della Società Toscana di Scienze Naturali, Pisa Mem., Ser. B, 87 (1980): 71-84.

De Sanctis A., Conti F., 2000 - National Parks in central Italy: from dream to sad reality. Plant talk, 21: 14-15.

De Santis E., Soldati R., 2011 - Guida fotografica alla flora di Campo Felice. Giardino Botanico Appenninico di Campo Felice e Pro Loco Lucoli, L'Aquila.

De Santis E., Soldati R., 2019 - Lucoli e i suoi fiori. La flora spontanea del territorio. C.M. Graf, L'Aquila.

Ferrarini E., Cecchi O., 2001 - Nuove specie del genere *Silene* (Caryophyllaceae) delle Alpi Apuane, dell'Appennino centrale (Italia) e della Francia meridionale. Webbia, 56 (2): 241-263.

Filibeck G., Cancellieri L., Bartolucci F., Becker U., Conti F., Maestri S., Mürz M., Schommer E., Sperandii M.G. & Becker T., 2020 - *Festuca valesiaca* Schleich. ex Gaudin newly discovered in the Central Apennines (Italy): a further example of steppe relict in the Abruzzo "dry valleys", Plant Biosystems, 154: 593-600, DOI: 10.1080/11263504.2019.1651784

Fiori A., 1923-1929 - Nuova Flora Analitica d'Italia 1-2. Firenze

Fiori A., 1943 - Flora Italica Criptogama. Pars V: Pteridophyta. Firenze

Fiori A., Béguinot A., 1917 - *Schedae ad Floram Italicam Exsiccatam. Series III. Centuriae* XXIII-XXIV. Tip. Fra.lli Gallina, fascicolo 13: 95-173. Padova.

Fiori A., Béguinot A., Pampanini R., 1907 - *Schedae ad Floram Italicam Exsiccatam. Centuriae* VI-VII. Nuovo Giorn. Bot. Ital., n.s., 14 (2): 69-116.

Foggi B., Ricceri C., 1993 - On the revaluation of *Silene staminea* Bertol. (Caryophyllaceae). Webbia, 47 (2): 193-201.

Frattaroli A.R., 1988 - La vegetazione della dolina "Fossa Raganesca" (Appennino centrale - Italia). Documents Phytosociologiques, n.s., 11 (1987): 491-503

Frattaroli A.R., Frizzi G., 1988 - Le piante endemiche dell'Appennino centrale: 3-4. 3. *Adonis distorta* Ten. (Ranunculaceae); 4. *Astragalus aquilanus* Anz. (Leguminosae). Micologia e Vegetazione Mediterranea, 3 (1): 23-30.

Frizzi G., Tammaro F., Guarrera P., 1996 - Studio floristico delle Gole di Celano (Abruzzo - Italia) e principali tipologie vegetazionali. Micologia e Vegetazione Mediterranea, 11 (1): 33-52.

Fumanti B., Lippolis P., Narducci G., 1986 - Segnalazioni Floristiche Italiane: 308-310. 308. *Epipogium aphyllum* Swartz (Orchidaceae); 309. *Ophrys lutea* (Gouan) Cav. (Orchidaceae); 310. *Orchis palustris* Jacq. (Orchidaceae). Informatore Botanico Italiano, 17 (1-2-3) (1985): 118-120.

Furnkranz D., 1964 - *Taraxacum apenninum* -ein altes Elements Mediterraner Gebirge. Österr. Bot. Z., 111: 231-239.

Furrer E., 1928 - Die Höhenstufen des Zentralapennin. Vierteljiahrsschrift Naturfosch. Ges. Zürich, 73: 642-663.

Galasso G, Domina G, Ardenghi NMG, Aristarchi C, Bacchetta G, Bartolucci F, Bonari G, Bouvet D, Brundu G, Buono S, Caldarella O, Calvia G, Cano-Ortiz A, Corti E, D'Amico FS, D'Antraccoli M, Di Turi A, Dutto M, Fanfarillo E, Ferretti G, Fiaschi T, Ganz C, Guarino R, Iberite M, Laface VLA, La Rosa A, Lastrucci L, Latini M, Lazzaro L, Lonati M, Lozano V, Luchino F, Magrini S, Mainetti A, Manca M, Mugnai M, Musarella CM, Nicolella G, Olivieri N, Orrù I, Pazienza G, Peruzzi L, Podda L, Prosser F, Ravetto Enri S, Restivo S, Roma-Marzio F, Ruggero A, Scoppola A, Selvi F, Spampinato G, Stinca A, Terzi M, Tiburtini M, Tornatore E, Vetromile R, Nepi C, 2019 - Notulae to the Italian alien vascular flora: 7. Italian Botanist 7: 157–182. https://doi.org/10.3897/italianbotanist.7.36386

Galasso G., Conti F., Peruzzi L., Alessandrini A., Ardenghi N.M.G., Bacchetta G., Banfi E., Barberis G., Bernardo L., Bouvet D., Bovio M., Castello M., Cecchi L., Del Guacchio E., Domina G., Fascetti S., Gallo L., Guarino R., Gubellini L., Guiggi A., Hofmann N., Iberite M., Jiménez-Mejías P., Longo D., Marchetti D., Martini F., Masin R.R., Medagli P., Musarella C.M., Peccenini S., Podda L., Prosser F., Roma-Marzio F., Rosati L., Santangelo A., Scoppola A., Selvaggi A., Selvi F., Soldano A., Stinca A., Wagensommer R.P., Wilhalm T., Bartolucci F. (2024): A second update to the checklist of the vascular flora alien to Italy, Plant Biosystems, 158(2): 297-340, DOI:10.1080/11263504.2024.2320129

Gallo L., 2012 - *Sempervivum ×luisae* Gallo (Crassulaceae) hybr. nov., endemico dell'Italia centrale. Ann. Mus. civ. Rovereto. Sez.: Arch., St., Sc. nat., 27: 287-296.

Gallo L., Conti F., 2015 - On the true identity of the plants recently referred to Sedum nevadense (Crassulaceae) in Abruzzo (Italy). Phytotaxa, 239(1): 43-54. http://dx.doi.org/10.11646/phytotaxa.239.1.4

Grande L., 1913 - Note di Floristica Napoletana. VIII-XL. Bulletino dell' Orto Botanico della Regia Università di Napoli, 3: 193-218.

Grande L., 1924 - Note di Floristica . Nuovo Giornale Botanico Italiano, 31: 105-160.

Grande L., 1925 - Note di Floristica. Nuovo Giornale Botanico Italiano, 32: 62-101.

Groves E., 1880 - Flora del Sirente. Nuovo Giornale Botanico Italiano, 12: 51-68.

Gruppo di Lavoro per la Conservazione della Natura della Società Botanica Italiana, 1971 - Censimento dei biotopi di rilevante interesse vegetazionale meritevoli di conservazione in Italia. Vol. I. Tip. Succ. Savini-Mercuri, Camerino.

Guarrera P.M., Tammaro F., 1996 - La Flora del M. Sirente e zone limitrofe (Appennino Abruzzese). Annali di Botanica (Roma), 52 (1994), Suppl. 11 (2): 267-381.

Guarrera P.M., Mastracci M., Tammaro F., 1996 - Su alcune piante nuove, riconfermate o notevoli rinvenute in Abruzzo. Informatore Botanico Italiano, 27 (2-3) (1995): 241-244.

Gussone G., 1826 - *Plantae rariores quas in itinere per oras jonii ac adriatici maris et per regiones Samnii et Aprutii collegit G. Gussone* . Ex Regia Typographia. Neapoli.

Hand R., 2001 - Revision der in Europa vorkommenden Arten von *Thalictrum* subsectio *Thalictrum* (Ranunculaceae). Botanik und Naturschutz in Hessen, Beiheft, 9. 358 pp.

Iberite M., Anzalone B., 2001 - *Sempervivum riccii* Iberite et Anzal., sp. nov. (Crassulaceae). Webbia, 56 (1): 165-173.

Iberite M., Pellicioni I., Bartolucci F., Conti F., 2008 - Notulae alla checklist della flora vascolare italiana 5. 1429. Informatore Botanico Italiano, 40 (1): 100-101.

Jeanmonod D., Bocquet G., 1983 - Propositions pour un traitement biosystématique du *Silene nutans* L. (Caryophyllaceae). Candollea, 38: 267-295.

Kalteisen M., Reinhard H.R., 1987 - Das Areal von *Ophrys promontorii* O. & E. Danesch. Mitteilungsblatt, Arbeitskreis Heimische Orchideen Baden-Württemberg; Beitrage zur Erhaltung und Erforschung Heimische Orchideen, 19 (4): 801-821.

Lastoria M., 1989 - Flora d'Abruzzo, 1. Deltagrafica, Teramo. 1-383.

Lastoria M., 2000 - Flora d'Abruzzo, 2. Deltagrafica, Teramo. 1-416.

Lastrucci L., Frignani F., Kaplan Z., 2010 - *Potamogeton schweinfurthii* and similar broad.leaved species in Italy. Webbia, 65(1): 147-160.

Lastrucci L., Gambirasio V., Prosser F., Viciani D., 2024 - First record of *Sparganium oocarpum* in Italy and new regional distribution data for *Sparganium erectum* species complex. Plant Biosystems, 158(4): 595–600.

Lucchese F., Lattanzi E., 1993 - Nuovo contributo alla Flora del Massiccio del Monte Velino (Appennino Abruzzese). Annali di Botanica (Roma), 49 (1991): 137-199.

Marchetti D., 2004 - Le Pteridofite d'Italia. Annali del Museo civico di Rovereto, Sez.: Arch., St., Sc. Nat., 19 (2003): 71-231.

Marcucci R., Tornadore N., 1997 - Cytological and taxonomical notes on *Allium arvense* Guss. (Alliaceae) in Italy. Webbia, 51 (2): 189-199.

Mariotti M.G., 1989 - Notes on some Italian species of Astrantia L. (Umbelliferae). Webbia 43(1), 1–17. https://doi.org/10.1080/00837792.1989.10670444

Martelli U., 1904 - Una passeggiata sul Monte Velino e Montagne della Duchessa. Bullettino della Società Botanica italiana, 11: 110-115.

Minutillo F., Tondi G., Conti F., 2010 - *Sedum nevadense* (Crassulaceae), new for the italian flora. Flora Mediterranea 19: 115-117.

Montelucci G., 1958 - Appunti sulla vegetazione del Monte Velino (Appennino Abruzzese). Nuovo Giornale Botanico Italiano, n.s., 65 (1-2): 237-334.

Montelucci G., 1962 - Itinerario geobotanico da Tivoli all'Aquila. Nuovo Giornale Botanico Italiano, n.s., 68 (3-4) (1961): 335-375.

Montelucci G., 1971 - Lineamenti floristici dell'Appennino Abruzzese. Lavori della Società Italiana di Biogeografia, n.s., 2: 13-67.

Murbeck S., 1933 - Monographie der Gattung *Verbascum* . Lunds Univ. Arsskrift 20:1-630

Orsenigo S., Montagnani C., Fenu G., Gargano D., Peruzzi L., Abeli T., Alessandrini A., Bacchetta G., Bartolucci F., Bovio M., Brullo C., Brullo S., Carta A., Castello M., Cogoni D., Conti F., Domina G., Foggi B., Gennai M., Gigante D., Iberite M., Lasen C., Magrini S., Perrino E., Prosser F., Santangelo A., Selvaggi A., Stinca A., Vagge I., Villani M., Wagensommer R.P., Wilhalm T., Tartaglini N., Duprè E., Blasi C., Rossi G., 2018 - Red Listing plants under full national responsibility: extinction risk and threats in the vascular flora endemic to Italy. Biological Conservation, 224:213-222.

Pace L., Catonica C., 1998 - Origine ed attualità del museo giardino alpino di Campo Imperatore. In: Burri E. (ed.), Aree protette in Abruzzo. Contributi alla conoscenza naturalistica ed ambientale: 140-163. Università dell'Aquila - Dip. Scienze Ambientali - Reg. Abruzzo. Carsa Ediz., Pescara.

Parlatore F., 1848-96 - Flora italiana, ossia descrizione delle piante che crescono spontanee o vegetano come tali in Italia e nelle isole aggiacenti, disposte secondo il metodo naturale. Volumi 1-11. Le Monnier, Firenze

Passalacqua N.G., Bernardo L., 2004 - The genus *Paeonia* L. in Italy: taxonomic survey and revision. Webbia, 59 (2): 215-268.

Peruzzi L., Bartolucci F., 2006 - *Gagea luberonensis* J.-M. Tison (Liliaceae) new for the italian flora. Webbia, 61 (1): 1-12.

Peruzzi L., Conti F., Bartolucci F., 2014 - An inventory of vascular plants endemic to Italy. Phytotaxa, 168 (1): 1-75.

Petriccione B., 1993 - Flora e Vegetazione del Massiccio del Monte Velino (Appennino Centrale), comprendente il territorio della Riserva Naturale Orientata «Monte Velino» e della foresta demaniale «Montagna della Duchessa» (con carta della vegetazione in scala 1:10.000). Minist. Agric. e Foreste, C.F.S., Collana Verde, 92. Tipo-Lito La Grotteria. Roma. 267 pp.

Petriccione B., 1994 - Flora and Vegetation mapping of Velino Massif (Abruzzo-Italy): a data source for a scientific management of a natural reserve. Fitosociologia, 26: 189-199.

Petriccione B., 2005 - Short-term changes in key plant communities of Central Apennines (Italy). Acta Botanica Gallica, 152 (4): 545-561.

Pezzetta A. 2016 – Le Orchidaceae della Provincia dell’Aquila, 85–104. Annales, Ser. hist. nat., · 26 ·(1): 85-104.

Pichi Sermolli R.E.G., 1955 - *Woodsia* R. Br. nell'Appennino. Webbia, 10 (2): 447-460.

Pignatti S., 1982 - Flora d'Italia. 3 voll. Edagricole. Bologna. Vol. 1, 790 pp.; Vol. 2, 732 pp.; Vol. 3, 780 pp.

Pirone G., 1987 - Il patrimonio vegetale della Provincia di Pescara. Amministr. Provinc. di Pescara. Pescara. 174 pp.

Pirone G., 1995 - Alberi, Arbusti e Liane d'Abruzzo. Cogecstre Edizioni, Penne. 543 pp.

Pirone G., 1996 - Una nuova associazione vegetale di gariga a *Phlomis fruticosa* L. nella Marsica (Abruzzo, Italia). Micologia e Vegetazione Mediterranea, 10 (2) (1995): 147-158.

Pirone G., 1997 - Il paesaggio vegetale di Rivisondoli: aspetti della flora e della vegetazione. Edigrafital. Teramo.

Pirone G., 2015 - Alberi, Arbusti e Liane d'Abruzzo, II ed. Cogecstre Edizioni, Penne. 624 pp.

Pirone G., Conti F., 1990 - Gole domestiche. D'Abruzzo, 3 (1): 12-17. Pescara.

Pirone G., Cutini M., 2002 - *Juniperus oxycedrus* L. subsp. *oxycedrus* and *Paliurus spina-christi* Miller scrubs in the intermontane areas of the Abruzzo region (Central Apennine, Central Italy). Fitosociologia, 39 (1): 81-96.

Pirone G., Tammaro F., 1997 - The hilly calciophilous garigues in Abruzzo (Central Apennines - Italy). Fitosociologia, 32 (1996): 73-90.

Pirone G., Corbetta F., Frattaroli A.R., Tammaro F., 1997 - Studi sulla Valle Peligna (Italia centrale, Abruzzo): la copertura vegetale. Quaderni di Provincia Oggi, 23/1: 81-119.

Pirone G., Frattaroli F., Ciaschetti G. 2018 - Contribution to knowledge of Apennine colline-submontane garigues on terrigenous rock types. Plant Sociology 55(1): 53-64.

Pomponi G., Frizzi G., Tammaro F., Bullini L., 1988 - Ricerche elettroforetiche in popolazioni di *Aubrieta columnae* Guss. nell'Italia centro-meridionale. Giorn. Bot. Ital., 122 (1-2), suppl. 1: 47.

Raffaelli M., Baldoin L., 1997 - Il complesso di *Biscutella laevigata* L. (Cruciferae) in Italia. Webbia, 52 (1): 87-128.

Ricci I., 1961 - Sul *Sempervivum italicum* n. sp. e sulle specie affini. Revisione critica. Ann. Bot. (Roma), 27 (1): 1-11.

Roma-Marzio F., Bartolucci F., Conti F., Di Martino L., Cecchi L., 2016 - Nuove Segnalazioni Floristiche Italiane 1. Flora vascolare (001-005). Notiziario della Società Botanica Italiana, 0: 85−86.

Rossi G., Montagnani C., Gargano D., Peruzzi L., Abeli T., Ravera S., Cogoni A., Fenu G., Magrini S., Gennai M., et al. (eds.). 2013. Lista Rossa della Flora Italiana. 1. Policy Species e altre specie minacciate. Comitato Italiano IUCN e Ministero dell’Ambiente e della Tutela del Territorio e del Mare. http://www.iucn.it/pdf/Comitato_IUCN_Lista_Rossa_della_flora_italiana_policy_species.pdf.

Rossi G., Orsenigo S., Gargano D., Montagnani C., Fenu G., Peruzzi L., Abeli T., Alessandrini A., Bacchetta G., Bartolucci F., et al. (eds.). 2020. Lista Rossa della Flora Italiana. 2. Specie endemiche e altre specie minacciate. Comitato Italiano IUCN e Ministero dell’Ambiente e della Tutela del Territorio e del Mare.

Rossi W., Capineri R., Teppner H., Klein E., 1987 - *Nigritella widderi* (Orchidaceae - Orchideae) in the Apennines (with 10 figures). Phyton (Horn), 27 (1): 129-138.

Rovelli E., Conti F., 1995 - Note floristiche per l'Appennino Centrale. Archivio Geobotanico, 1 (2): 185-188.

Segota V., Gligora Udovič M., Levkov Z., Bogdanović S., Rimac A., Alegro A., Šušnjara M., Doboš M., Temunović M., Žutinić P., Budinski I., Conti F., Dorić V., Kulaš A., Bučar M., Cindrić M., Engelen A., Ilić B., Jeričević M., Maslać Mikulec M., Šabanović E., Vuković N., Borovečki-Voska Lj., Ćato S., Ćetković I., Hudina T., Huška D., Jantol N., Jeričević N., Limić I., Loos U., Klarić M., Kovačević M., Rogošić M., Škunca L., Tkalčec Z., Jurina D., Stenger-Kovács C. (2025):ž Novi nalazi rijetkih i nebilježenih vaskularnih biljaka, mahovina, lišaja, gljiva i algi u Hrvatskoj i susjednim zemljama – 2. Glas. Hrvat. bot. druš. 13(1): 86-122.

Soest J.L. van, 1966 - New *Taraxacum* species from Europe [I, II, III, IV]. Proc. Kon. Ned. Akad. Wetensch., Biol. Chem. Geol. Phys. Med. Sci. 79: 171-190.

Stanisci A., 1997 - Gli arbusteti altomontani dell'Appennino Centrale e Meridionale. Fitosociologia, 34: 3-46.

Steinberg C., 1953 - Contributo allo studio floristico e fitogeografico degli alti pascoli della Montagna della Duchessa (Appennino abruzzese). Nuovo Giornale Botanico Italiano, n.s., 59 (2-4) (1952): 201-251.

Stinca A., Bartolucci F., Conti F., Di Cecco V. 2021a – Schede delle piante di progetto. In: Conti F., Di Martino L. (eds.) Life Floranet. Edizioni Parco Nazionale della Maiella. Pp. 167-199.

Stinca A., Musarella C.M., Rosati L., Laface V.L.A., Licht W., Fanfarillo E., Wagensommer R.P., Galasso G., Fascetti S., Esposito A., et al. , 2021b - Italian Vascular Flora: New Findings, Updates and Exploration of Floristic Similarities between Regions. Diversity, 13(11): 600. https://doi.org/10.3390/d13110600

Tammaro F., 1971 - Su alcune entità di M. Sirente (Appennino Abruzzese) di particolare interesse fitogeografico. Lav. Soc. Ital. Biogeogr., n.s., 2: 89-105.

Tammaro F., 1982 - Segnalazioni Floristiche Italiane: 99-106. 99. *Arisarum vulgare* Targ.-Tozz. (Araceae); 100. *Gladiolus dubius* Guss. (Iridaceae); 101. *Limonium oleifolium* Miller subsp. *oleifolium* (Plumbaginaceae); 102. *Polycarpon alsinifolium* (Biv.) DC. (Caryophyllaceae); 103. *Athamantha sicula* L. (Umbelliferae); 104. *Alkanna tinctoria* Tausch (boraginaceae); 105. *Onobrychis arenaria* (Kit.) DC. subsp. *arenaria* (Leguminosae); 106. *Cynoglossum cheirifolium* L. (Boraginaceae). Inform. Bot. Ital., 13 (1) (1981): 51-52.

Tammaro F., 1984 - Segnalazioni Floristiche Italiane: 247-254. 247. *Vitex agnus-castus* L. (Verbenaceae); 248. *Succisa pratensis* Moench (Dipsacaceae); 249. *Quercus crenata* Lam. (Fagaceae); 250. *Potamogeton berchtoldii* Fieber (Potamogetonaceae); 251. *Myosotis caespitosa* C.F. Schultz (Boraginaceae); 252. *Trifolium* dubium Sibth. (Leguminosae); 253. *Apium inundatum* (L.) Reichenb. fil. (Umbelliferae); 254. *Oenothera chicaginensis* De Vries ex Renner (Onagraceae). Informatore Botanico Italiano, 15 (1) (1983): 86-89.

Tammaro F., 1988 - La distribuzione del genere *Carex* L. (Cyperaceae) in Abruzzo. Inform. Bot. Ital., 19 (3) (1987): 287-304.

Tammaro F., 1990 - Re-identification and characterization of Ligusticum cuneifolium Guss. Umbelliferae (gr. L. lucidum Miller), a neglected endemic entity from Central Italy. Ann. Bot. (Roma), 47 (1989): 215-225.

Tammaro F., 1998 - Il paesaggio vegetale d'Abruzzo. Aree protette, biotopi ed itinerari botanici: dalle zone costiere ai massicci montuosi. Cogecstre Edizioni, Penne. 670 pp.

Tammaro F., Frizzi G., 1984 - Segnalazioni Floristiche Italiane: 164-170. 164. *Epilobium obscurum* Schreber (Onagraceae); 165. *Romulea bulbocodium* (L.) Seb. et Mauri (Iridaceae); 166. *Artemisia variabilis* Ten. (Compositae); 167. *Ononis rotundifolia* L. (Leguminosae); 168. *Romulea columnae* Seb. et Mauri (Iridaceae); 169. *Hieracium staticifolium* All. (Compositae); 170. *Aster novi-belgii* L. (Compositae). Informatore Botanico Italiano, 14 (2-3) (1982): 286-288.

Tammaro F., Frizzi G., 1990 - *Viola eugeniae* Parl. subsp. *levieri* (Parl.) A. Schmidt, misconosciuto endemismo della flora italiana. Giorn. Bot. Ital., 124 (1): 112.

Tammaro F., Pace L., 1994 - Considerazioni floristiche sulla Conca del Fucino. In: VV.AA., Il lago Fucino e il suo emissario. Carsa Edizioni, Pescara: 78-95.

Tammaro F., Pogliani M., 1977 - *Andrachne telephioides* L. nella Valle dell'Aterno, nuovo reperto per la Flora Abruzzese. Webbia, 32 (1): 135-145.

Tammaro F., Visca C., 1987 - Segnalazioni Floristiche Italiane: 465-477. 465. *Dianthus vulturius* Guss. et Ten. (Caryophyllaceae); 466. *Oxytropis caputoi* Moraldo et La Valva (Leguminosae); 467. *Orlaya daucorlaya* Murb. (Umbelliferae); 468. *Serratula tinctoria* L. subsp. *tinctoria* var. *pinnata* Kit. (Compositae); 469. *Colchicum neapolitanum* (Ten.) Ten. (Liliaceae); 470. *Groenlandia densa* (L.) Fourr. (Potamogetonaceae); 471. *Oenanthe fistulosa* L. (Umbelliferae); 472. *Ranunculus serpens* Schrank (Ranunculaceae); 473. *Caltha palustris* L. (Ranunculaceae); 474. *Consolida pubescens* (DC.) Soò (Ranunculaceae); 475. *Cotinus coggygria* Scop. (Anacardiaceae); 476. *Asteriscus aquaticus* (L.) Less. (Compositae); 477. *Leontodon autumnalis* L. subsp. *autumnalis* (Compositae). Informatore Botanico Italiano, 19 (2): 181-184.

Tammaro F., Veri L., Frizzi G., 1974 - Indagine botanica su alcuni pascoli montani abruzzesi. Rivista Abruzzese, 27 (4): 32 pp.

Tammaro F., Veri L., Chichiriccò G., 1980 - Segnalazioni Floristiche Italiane: 28-35. 28. *Elaeoselinum asclepium* (L.) Bertol. subsp. *asclepium* (Umbelliferae); 29. *Myrrhoides nodosa* (L.) Cannon (Umbelliferae); 30. *Iberis pruitii* Tineo var. *tenoreana* (DC.) Fiori (Cruciferae); 31. *Astragalus aquilanus* Anzalone (Leguminosae); 32. *Scrophularia umbrosa* Dumort. (Scrophulariaceae); 33. *Silene noctiflora* L. (Caryophyllaceae); 34. *Anchusa undulata* L. subsp. *hybrida* (Ten.) Coutinho (Boraginaceae); 35. *Asphodeline liburnica* (Scop.) Rchb. (Liliaceae). Informatore Botanico Italiano, 11 (2) (1979): 174-176.

Tammaro F., Pignatti S., Frizzi G., 1982 - *Goniolimon italicum* (Plumbaginaceae), una nuova specie rinvenuta nei pressi di L'Aquila (Appennino Centrale). Webbia, 36 (1): 39-46.

Tammaro F., Sabatini L., Mastracci M., 1988 - Cartografia floristica di entità della Flora d'Abruzzo: le Ombrellifere. Boll. Ass. Ital. Cart., 72-73-74: 709-725.

Tenore M., 1830 - Succinta relazione del viaggio fatto in Abruzzo ed in alcune parti dello Stato Pontificio dal Cavalier Tenore nell'Està del 1829. Stamperia della Società Filomatica: [1]-90 [91].

Tenore M., 1831 - *Sylloge Plantarum Vascularium Florae Neapolitanae*. Neapoli ex Typographia Fibreni.

Tenore M., 1842 - *Ad Florae Neapolitanae Syllogem, appendix quinta*. Neapoli, Typ. Tizzano.

Vannicelli Casoni L., Di Francesco M., De Pasquale L., Di Pietro R., 1998 - Indagine interdisciplinare sulla conservazione dei monumenti. Una proposta metodologica per la tutela e la valorizzazione di alcuni insigni monumenti siti nella provincia dell'Aquila. In: Burri E. (ed.), Aree protette in Abruzzo. Contributi alla conoscenza naturalistica ed ambientale: 140-163. Università dell'Aquila - Dip. Scienze Ambientali - Reg. Abruzzo. Carsa Ediz., Pescara.

Veri L., Tammaro F., 1980 - Aspetti vegetazionali del Monte Sirente (Appennino Abruzzese). C.N.R., Coll. Progr. Final. "Promozione della Qualità dell'ambiente", AQ/1/83. Roma. 22 pp. + map.

Viegi L., Cela Renzoni G., D'Eugenio M.L., Rizzo A.M., 1990 - Flora esotica d'Italia: le specie presenti in Abruzzo e in Molise (revisione bibliografica e d'erbario). Archivio Botanico Italiano, 66 (1-2): 1-128.

Zanotti A.L., Cristofolini G., 1994 - Taxonomy and Chorology of *Helleborus* L. sect. *Helleborastrum* Spach in Italy. Webbia, 49 (1): 1-24.
